# Revealing the Impact of Mitochondrial Fitness During Early Neural Development Using Human Brain Organoids

**DOI:** 10.3389/fnmol.2022.840265

**Published:** 2022-04-29

**Authors:** Alejandra I. Romero-Morales, Vivian Gama

**Affiliations:** ^1^Department of Cell and Developmental Biology, Vanderbilt University, Nashville, TN, United States; ^2^Vanderbilt Center for Stem Cell Biology, Vanderbilt University, Nashville, TN, United States; ^3^Vanderbilt Brain Institute, Vanderbilt University, Nashville, TN, United States

**Keywords:** stem cells, glycolysis, oxidative phosphorylation, mitochondria, neural precursor cells, neural rosettes, brain organoids

## Abstract

Mitochondrial homeostasis -including function, morphology, and inter-organelle communication- provides guidance to the intrinsic developmental programs of corticogenesis, while also being responsive to environmental and intercellular signals. Two- and three-dimensional platforms have become useful tools to interrogate the capacity of cells to generate neuronal and glia progeny in a background of metabolic dysregulation, but the mechanistic underpinnings underlying the role of mitochondria during human neurogenesis remain unexplored. Here we provide a concise overview of cortical development and the use of pluripotent stem cell models that have contributed to our understanding of mitochondrial and metabolic regulation of early human brain development. We finally discuss the effects of mitochondrial fitness dysregulation seen under stress conditions such as metabolic dysregulation, absence of developmental apoptosis, and hypoxia; and the avenues of research that can be explored with the use of brain organoids.

## Introduction

*From the brain, and from the brain only, arise our pleasures, joys, laughter and jests, as well as our sorrows, pains, griefs, and tears. Through it, in particular, we think, see, hear, and distinguish the ugly from the beautiful, the bad from the good, the pleasant from the unpleasant, in some cases using custom as a test, in others perceiving them from their utility.*

   *-Hippocrates (c. 460 B.C. - c. 370 B.C.) (LOEB Classical Library, 2021)*

Studies on human brain function and development have been a topic of controversy and ethical concerns throughout scientific history (Di Pietro et al., 2012; Bredenoord et al., 2017; Farahany et al., 2018; National Academies of Sciences Engineering and Medicine, 2021). It is undeniable that access to human-derived material has contributed greatly to the advance of therapies and drugs and expanded our understanding of the processes that have been studied in other biological models. The comparison between different organisms is critical to assess the specie-specific differences that have arisen from evolution and the similarities that have been conserved and can be advantageous for the use of non-human models in translational science.

The human brain has unique characteristics that separate *Homo sapiens* from even the closest primate relatives. Cortical expansion, upper neuronal layer enlargement, increased neuronal diversity and function, complex connectivity and circuitry, are unique features of the human brain (Fuster, 2002; Kaas, 2008; Forbes and Grafman, 2010; Molnár et al., 2019; Dehay and Kennedy, 2020; Heide et al., 2020). The study of how these differences evolved has proven challenging as access to developing human tissue has been limited due to ethical considerations (Presidential Commission for the Study of Bioethical, 2015; Greely et al., 2016; Farahany et al., 2018). Additionally, the understanding of the molecular basis of various human neurological disorders has been hindered by the complexity of genetic alterations (Geschwind and Flint, 2015).

Although simple in comparison to the human central nervous system (CNS), non-human models have shown to reproduce the developmental stages, cellular composition, cytoarchitecture, and activity seen in the human brain (Arlotta and Paşca, 2019). The advent of human cellular models, like pluripotent stem cell (PSCs)-derived systems, and the intersection with research in non-human models have propelled human brain development research.

Mitochondrial diseases, which are traditionally linked to disruption in OXPHOS, are usually associated with neurological phenotypes such as developmental delay, atrophy, and epileptic encephalopathy (Ortiz-González, 2021; Povea-Cabello et al., 2021). Due to advances in exome sequencing, the range of causal mutations for mitochondrial diseases has expanded to include not only metabolic genes but proteins that affect mitochondrial shape, cristae stability, recycling, motility, and interactions with other organelles (Baum and Gama, 2021). Thus, the capacity of the mitochondria to adapt and maintain its homeostasis its key for the correct execution of the intrinsic developmental programs of neural and corticogenesis, while also being responsive to environmental and intercellular signals.

Remodeling of the mitochondrial network as human pluripotent stem cells (hPSCs) commit to a neuronal cell fate is necessary for their survival and function (Chan, 2012; Schwarz, 2013; Khacho et al., 2016; Khacho and Slack, 2018; Iwata et al., 2020). Likewise, neural processes such as development, migration, maturation, and plasticity; demand high levels of energy (Klein Gunnewiek et al., 2020). Mitochondrial fragmentation is a hallmark of glycolytic cell types such as stem cells (Teslaa and Teitell, 2015; Chen and Chan, 2017; Zhang H. et al., 2018; Rastogi et al., 2019), and the ability to transition to a more complex and elongated network that facilitates aerobic respiration is crucial for the survival of the newborn neurons (Chan, 2012; Schwarz, 2013; Zheng et al., 2016).

in the first part of this review we provide an overview of cortical development and the use of PSC models. In the second part, we discuss our current understanding of mitochondrial and metabolic regulation during early human brain development and the effects of mitochondrial fitness dysregulation seen under stress conditions such as metabolic dysregulation, absence of developmental apoptosis, and hypoxia.

### Human Brain Development: Finely Orchestrated Events of Commitment, Migration, and Expansion

The human CNS is composed of approximately 86 billion neurons, with a roughly equal number of glial cells (Herculano-Houzel et al., 2015). Ninety-nine percent of all neurons are located inside the cranium. The cerebral cortex is composed by ∼20% of all neurons, although it represents ∼50% of the CNS volume (Molnár and Pollen, 2014; Herculano-Houzel et al., 2015, 2016; Sousa et al., 2017).

The human brain has specie-specific characteristics that highlight the need for more representative modeling of development. For example, the developing brain has expanded proliferative zones (e.g., outer subventricular zone, SVZ) with diverse subtypes of neural stem and progenitor cells [e.g., outer radial glia (oRGCs)] that facilitate the expansion of the neocortex (Bystron et al., 2008; Sousa et al., 2017; Molnár et al., 2019). The timing and duration of neurogenesis is also a factor to consider when examining the differences between species. Extended human neurogenesis results in an increased number of progenitor cells that give rise to larger neocortical structures with increased number of upper-layer neurons (Smart et al., 2002; Hutsler et al., 2005; Taverna et al., 2014). In addition to the ventricular zone (VZ) and the cortical plate (discussed in detail below), the human brain contains an additional layer, called the outer SVZ. The outer SVZ is characteristic for primate brains and it contains oRGCs or basal radial glia cells. In rodent brains, these cells are not present, or present in only very small numbers (Wang L. et al., 2011; Wang X. et al., 2011; Kalebic and Huttner, 2020). But, in primates, they act as a transit amplifying population during neurogenesis (Fietz et al., 2010; Hansen et al., 2010; Sauerland et al., 2018) contributing to the expansion of the cortex. Developmental studies in rodents segregate the birth of excitatory neurons and interneurons to the progenitors in the cortex and ganglionic eminence, respectively, yet lineage tracing of primary human neural progenitors show that individual cortical progenitors have the capacity to generate both excitatory and cortical interneurons (Delgado et al., 2022).

Human brain development is a prolonged and intricate process that starts around 2 weeks post conception and continues until early adulthood. The initial stages of this process rely primarily on the genetic control and the correct activation of neural programs, although, environmental factors can also affect the efficiency of the process.

#### Initial Development: Neural Tube Formation

The genesis of the nervous system initiates about 2 weeks post conception. At this stage, the developing embryo is organized as a three-layered spherical structure. Cells in the ectoderm, one of the three germ layers, thicken to form the neural plate. The lateral edges of the neural plate will give rise to the neural fold that will join at the midline forming the neural tube. The closure of these tube occurs from the center to the cranial and caudal ends. Developmental defects at this point can cause anencephalia or spina bifida. The formation of the neural tube leads to the formation of the CNS by the cells located in the inner part of the tube, while the outer cells will give rise to the peripheral nervous system (O’Rahilly and Müller, 2005; Bayer and Altman, 2007).

Once the neural tube closes, around 4 weeks post-conception, the cranial end will expand to become a three-vesicle structure: the prosencephalon, the mesencephalon, and the rhombencephalon. By week 5 post-conception, the prosencephalon will give rise to the telencephalon, which will correspond with the forebrain and includes the cerebral hemispheres; and the diencephalon that will develop into the thalamus and hypothalamus (O’Rahilly and Müller, 2008). The mesencephalon will give rise to the mature midbrain; and the rhombencephalon will generate the metencephalon which in time will derived the pons and the cerebellum, and the myelencephalon that will develop into the medulla (O’Rahilly and Müller, 2005, 2010; Bayer and Altman, 2007).

Morphogen signaling during this period is crucial for the establishment of the development axis. The notochord, a structure derived from the axial mesoderm immediately ventral to the ectoderm (Grow, 2018; Di Gregorio, 2020), and the somites, transient paired structures derived from mesenchymal paraxial mesoderm that flank the neural tube (Cook et al., 2017), define the dorso-ventral axis of the embryo by releasing different signaling molecules (Seal and Monsoro-Burq, 2020).

##### Fibroblast Growth Factor

FGF8 is produced by the paraxial mesoderm and it’s downregulated before neural differentiation (Bertrand et al., 2000; Muñoz-Sanjuán and Brivanlou, 2002). This downregulation is necessary for the expression of early neural transcription factors such as NEUROM, PAX3, HES4, TFAP2A, and MSX1 in the neural tube (Bang et al., 1997; Diez del Corral et al., 2002; Monsoro-Burq et al., 2005; Garnett et al., 2012).

##### Retinoic Acid

Retinoic acid (RA) signaling is generated by the paraxial mesoderm for the induction of neural differentiation and patterning by downregulating fibroblast growth factor (FGF) production (Franco et al., 1999; Colas and Schoenwolf, 2001; Diez del Corral and Storey, 2004). RA generated in the SVZ of the basal ganglia is required for GABAergic differentiation (Chatzi et al., 2011), whereas the RA generated in the meninges regulates cortical neuron generation (Siegenthaler et al., 2009). Moreover, classic studies in *Xenopus* implicated WNT (blend of the names Wingless and Int-1), RA and FGF as dorsalizing factors (Ruiz I Altaba and Jessell, 1991; Sharpe, 1991; Altmann and Brivanlou, 2001).

##### Sonic Hedgehog

Sonic hedgehog (SHH) is produced by the notochord for the ventralization of neural cell types in a concentration dependent manner (Jessell and Dodd, 1990; Echelard et al., 1993; Liem et al., 2000). Mouse embryos lacking SHH fail to form ventral telencephalons and show a marked reduction on the expression of ventral markers (Rallu et al., 2002), and its ectopic expression is sufficient to induce the expression of ventral telencephalic *in vitro* and *in vivo* (Gaiano et al., 1999).

##### WNT

The activation of WNT signaling, especially from Wnt3a, is necessary for the induction of posterior patterning (McGrew et al., 1995; Kiecker and Niehrs, 2001). WNT signaling repression is critical for the generation of the anterior neural fate (Yamaguchi, 2001).

##### Bone Morphogenetic Protein

Bone morphogenetic protein (BMP) are produced by the non-neural ectoderm and the ventral mesoderm. High BMP signaling in the ventral ectoderm promotes the epidermal fate and represses the neural fate. BMP antagonists, such as Noggin, Chordin, Cerberus and Follistatin, are secreted by the Spemann-Mangold organizer (Spemann and Mangold, 1924, 2001; Muñoz-Sanjuán and Brivanlou, 2002), generating a low-to-high gradient of BMP signaling from the midline toward the lateral zones that allows for the neural specification of the dorsal ectoderm (Lee et al., 1998; Barth et al., 1999; Lee and Jessell, 1999; Liem et al., 2000; Nguyen et al., 2000; Ybot-Gonzalez et al., 2007).

#### Neural Progenitor Cell Expansion and Radial Glia Cell Proliferation

**FIGURE 1:**
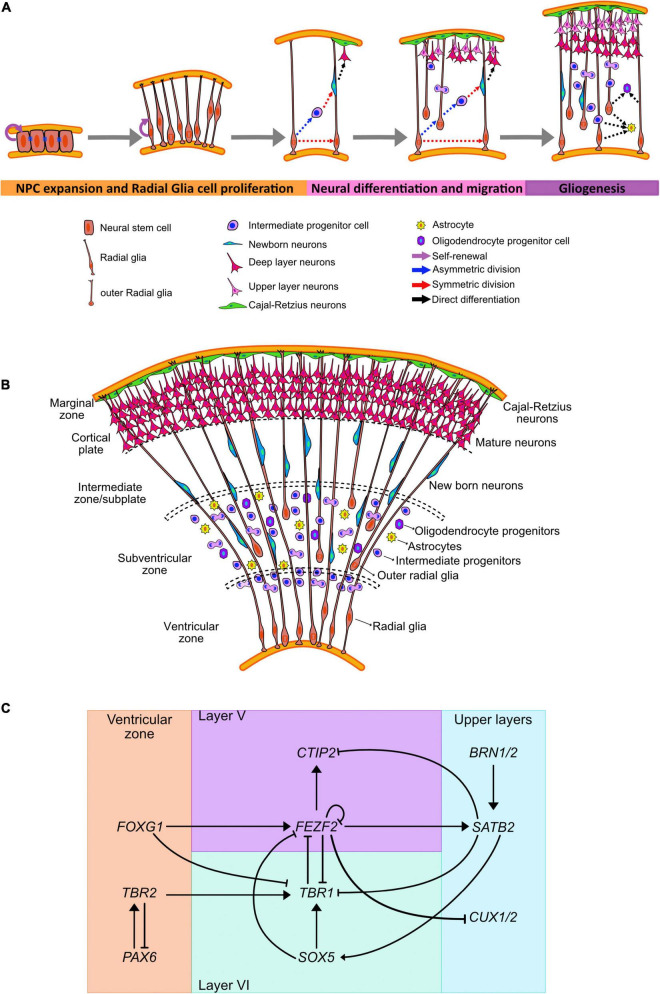
Human neocortical development. **(A)** Schematic illustration of neurogenesis in the human cortex. **(B)** Cortical expansion in humans allows for the formation of different areas where progenitor lineages migrate, proliferate, and differentiate. **(C)** Transcriptional regulators and genes governing cell fate acquisition and specification during neurogenesis.

Once the closure of the neural tube is complete, the cells lining the lumen of the tube will develop into the ventricles. This single cell layer of neuroepithelia is a pseudostratified epithelium composed by neuroepithelial progenitor cells (Götz and Huttner, 2005; Figure 1A). These are highly polarized along the apical-basal axis (Chenn et al., 1998). Transmembrane proteins such as prominin-1 are found in the apical plasma membrane, while tight and adherens junctions are present at the apical end of the lateral plasma membrane (Aaku-Saraste et al., 1996; Chenn et al., 1998; Zhadanov et al., 1999; Manabe et al., 2002). Receptors for basal lamina components, such as integrins, are located in the basal plasma membrane that is in contact with the basal lamina (Wodarz and Huttner, 2003).

This epithelium appears as stratified due to the cell nuclei migrating up and down the apical–basal axis during the cell cycle in a process known as interkinetic nuclear migration (His, 1889; Schaper, 1897; Sauer, 1935; Frade, 2002; Bayer and Altman, 2007; Rodrigues et al., 2019). In this process the nuclei migrate to the apical side during mitosis and remains basally during the S phase. This movement exposes the nuclei to diffused morphogens, such as Notch receptor ligand Delta, and influence the fate of the daughter cells (Chenn and McConnell, 1995; Chenn et al., 1998; Murciano et al., 2002; Del Bene et al., 2008; Spear and Erickson, 2012; Azizi et al., 2020).

The neuroepithelial progenitor cells divide in either a symmetric or asymmetric manner (Huttner and Brand, 1997; Götz and Huttner, 2005). Symmetric cell division generate two daughter cells that remain mitotically active and can expand the population of progenitor cells (Kornack and Rakic, 1995; Mione et al., 1997). In contrast, in an asymmetric division at least one of the daughter cells exit the cell cycle and differentiates into a specialized cell (Reid et al., 1997; Qian et al., 1998; Fishell and Kriegstein, 2003; Wodarz and Huttner, 2003).

Rounds of cell division from the neuroepithelial cells form several layers surrounding the lumen (Figures 1A,B). The inner-most apical layer becomes populated with the progenitor cells and due to the proximity with the ventricles is known as the VZ. As asymmetric division mark the beginning of neurogenesis, neuroepithelial cells have been shown to downregulate the expression of tight junction and certain apical plasma membrane proteins (Aaku-Saraste et al., 1996, 1997).

Neuroepithelial cells give rise to a progenitor cell type that is more cell fate-restricted, the radial glial cells (RGCs) (Magini, 1888; Koelliker, 1896; Bentivoglio and Mazzarello, 1999). These cells can be observed in the developing embryo as early as week 6 post conception (Choi, 1981). RGCs are characterized by bipolar processes extending to reach the pial and ventricular surfaces, while their cell bodies remain in the VZ (Rakic, 1972, 1978; Levitt and Rakic, 1980; Haubensak et al., 2004). These processes help guide the radial migration of newborn neurons from the VZ. Moreover, these cells share some astrocytic characteristics such as glycogen granules and the expression of the intermediate filament protein, glial fibrillary acidic protein (GFAP) (Levitt and Rakic, 1980; Choi, 1981) and VIMENTIN, as well as brain-lipid-binding protein (BLBP) (Hartfuss et al., 2001). RGCs also maintain the expression of the neuroepithelial marker NESTIN (Pixley and de Vellis, 1984; Chanas-Sacre et al., 2000; Campbell and Götz, 2002), apical-basal polarity with the presence of adherence junctions (Aaku-Saraste et al., 1996; Wodarz and Huttner, 2003), basal lamina contact (Halfter et al., 2002), and interkinetic nuclear migration. Regionalization of the brain is also an ongoing process at this stage. Dorsal RGCs express the neural progenitor marker Paired box protein 6 (PAX6) (Götz et al., 1998) which commit them into a cortical fate; while the ventral RGCs express the cellular retinol-binding protein (RBP-1) (Toresson et al., 1999) and will commit to form the basal ganglia.

Prior to the peak in neurogenesis, RGCs divide symmetrically to amplify the progenitor cell population (Figure 1A). However, during the peak phase of neurogenesis, RGCs primarily divide asymmetrically to both self-renew and give rise to outer RGCs (oRGCs), intermediate progenitors (IPCs), astrocytes, or neurons (Malatesta et al., 2000, 2003; Alvarez-Buylla et al., 2001; Miyata et al., 2001; Noctor et al., 2001; Anthony et al., 2004; Kriegstein and Alvarez-Buylla, 2009). The cell fate specification during the asymmetrical neurogenesis has been associated with the subcellular distribution of the mammalian partition defective protein 3 (mPAR3) by differentially regulating Notch signaling activity in the two daughter cells (Bultje et al., 2009).

Outer radial glia and intermediate progenitors will establish the SVZ around week 9 post conception (Zecevic et al., 2005). oRGs retain the basal processes but lack the apical junctions (Miyata et al., 2001), and undergo a distinct migratory behavior or mitotic somal translocation before undergoing cell division (Fietz et al., 2010; Hansen et al., 2010; Wang X. et al., 2011). These cells can also undergo proliferative and asymmetric cell divisions and require Notch signaling to induce neuronal differentiation (Kowalczyk et al., 2009; Hansen et al., 2010). IPCs are transient amplifying cells with limited proliferative divisions, which are predominantly symmetrical to produce two neurons (Noctor et al., 2001; Haubensak et al., 2004; Wu et al., 2005; Sessa et al., 2008, 2010; Kowalczyk et al., 2009). These cells have multipolar morphology and are not anchored to either the apical or basal cortical surface (Noctor et al., 2001; Haubensak et al., 2004). IPCs contribute to radial expansion and folding of the human brain (Englund, 2005; Baala et al., 2007; Miller et al., 2019) and have been associated to the generation of the upper cortical layers (Martínez-Cerdeño et al., 2006; Arnold et al., 2008; Lv et al., 2019).

The different types of progenitors can be identified not only by their morphology and presence of polarizing membrane proteins, but by the expression of certain fate markers (Figure 1C). Radial glia express PAX6, a homeodomain transcription factor, in contrast IPCs upregulate T-Box Brain Protein 2 (TBR2), a T-domain transcription factor, and downregulate PAX6 (Englund, 2005). oRGCs and SVZ progenitors also express the non-coding RNA subventricular-expressed transcript 1 (SVET1) (Tarabykin et al., 2001), as well as the transcription factor Cut Like Homeobox2 (CUX2) (Nieto et al., 2004; Zimmer et al., 2004). Moreover, oRGs preferentially express genes related to extracellular matrix formation, migration, and stemness (Pollen et al., 2015; Nowakowski et al., 2017; Fan et al., 2020).

#### Corticogenesis: Neural Differentiation and Migration

The generation of cortical neurons, or corticogenesis, can be distinguished by the enlargement of the SVZ that has an inner (ISVZ) and outer region (OSVZ), and it is separated by a thin fiber layer (Zecevic et al., 2005). Cells undergoing mitosis can be observed in all levels of the SVZ, in contrast with the VZ where mitotic cells are only found in the ventricular surface (Smart et al., 2002; Figures 1A,B).

Early born neurons migrate away from the ventricular surface, segregating themselves from the progenitors and forming the pre-plate. The first wave of neurons is composed by specialized pioneering cells or Cajal-Retzius neurons. These neurons organize adjacent to the pial surface forming the marginal zone, that in the adult human brain correspond with Layer I (Marin-Padilla, 1978; Raedler and Raedler, 1978) and that act as the stop sign for neuronal migration (Tissir and Goffinet, 2003). These cells secrete REELIN, a large extracellular matrix glycoprotein. REELIN regulate processes of neuronal migration and positioning in the developing brain by controlling cell–cell interactions specifically by binding to the transmembrane receptors very low–density lipoprotein receptor (Vldlr), and apolipoprotein E receptor 2 (ApoER2) present on migrating neurons (D’Arcangelo et al., 1995; Hiesberger et al., 1999). The binding of REELIN to the previously mentioned receptors induces the phosphorylation of Disabled 1 (Dab1), a cytosolic protein that activates tyrosine kinases (Trommsdorff et al., 1999; Kim et al., 2002) that in time modulates the phosphorylation of Tau affecting the assembly and stability of the neuronal cytoskeleton (Hiesberger et al., 1999; Rice and Curran, 2001). REELIN also interacts with α3β1 integrin, which regulates neuron–glia interactions by promoting the detachment of the migratory neuron from the radial glia via Dab1 and is required to achieve proper laminar organization (Dulabon et al., 2000).

As new neurons are born and start migrating into the pre-plate, this area is divided into the marginal zone, which is pushed outwards, and the subplate (Molnár et al., 2019). The subplate is a voluminous yet transient compartment in the cerebral wall composed of post-migratory and migratory neurons, glia and axons; as well as of abundant extracellular matrix (Kostovic and Rakic, 1990; Hoerder-Suabedissen and Molnár, 2015). This structure is key for the correct formation and subsequent function of the brain as it is involved in the formation of neural circuits (Kostovic and Rakic, 1990).

Between the marginal zone and the subplate, newborn neurons migrate and organize forming the cortical plate around post-conception weeks 8–9 (Clancy et al., 2001). In this area, the nascent neurons stop migrating and differentiate into their final laminar identity in an inside-out fashion (Greig et al., 2013), with newer born neurons positioning closer to the marginal zone (Molyneaux et al., 2007; Leone et al., 2008; Marín-Padilla, 2014; Shibata et al., 2015). Hence, neurons of the deepest layers (VI and V) are generated at the earliest stages, followed by neurons of layers IV, III, and II. For the neuronal maturation process, final positioning of the neurons is required; and can last until early adulthood in humans (Huttenlocher, 1979; Petanjek et al., 2008, 2011).

Cortical neurons are generated primarily (∼80%) by IPCs, while the other 10–20% can be traced back to RGCs (Kowalczyk et al., 2009; Vasistha et al., 2015). Moreover, cortical progenitors undergo fate restriction as they produce new neurons. Late cortical progenitors are not capable to generate earlier neuronal fates even when exposed to environments that mimic the early stages of corticogenesis (Walsh and Cepko, 1993; Frantz and McConnell, 1996; Desai and McConnell, 2000). This fate restriction has been associated with changes in the length of the cell cycle and the number of divisions before terminal differentiation (Calegari et al., 2005; Shen et al., 2006; Pilaz et al., 2009).

Fate acquisition has also been linked with the expression of subtype-specific transcription factors expressed in progenitors before neurogenesis; suggesting an early commitment to a particular cortical layer (Greig et al., 2013; Figure 1C). The transition from the pioneering Cajal-Retzius cells into the generation of deep-layer neurons has been shown to be mediated by the repression of the transcription factor Forkhead Box G1 (FOXG1) in neural progenitors (Hanashima et al., 2004).

Deep-layer progenitors express the transcription factors FEZF2 (Fez family zinc finger 2), OTX1 (Orthodenticle Homeobox 1), and EMX2 (Empty Spiracles Homeobox 2) (Frantz et al., 1994; Leingärtner et al., 2003; Inoue et al., 2004; Chen et al., 2005; McKenna et al., 2011; Lodato et al., 2014) that have been associated with neurons present in layers VI and V. OTX1, for example, is expressed in VZ precursors that will give rise to cortical layers VI and V, but it is downregulates in progenitor cells that are committed to upper layer neurogenesis (Frantz et al., 1994; Weimann et al., 1999).

The generation of layer VI neurons, around post-conception weeks 11–12 (Clancy et al., 2001), is mediated by the expression of the transcription factor T-Box Brain Protein 1 (TBR1) (Hevner et al., 2001; Bedogni et al., 2010). TBR1 downregulates the expression of FEZF2 (Han et al., 2011) and CTIP2 (COUP-TF-Interacting Protein 2) (McKenna et al., 2011) by binding to the FEZF2 gene and inhibiting its transcription. FEZF2 acts upstream of CTIP2 to control the differentiation of layer V neurons (Arlotta et al., 2005; Chen et al., 2005) by reducing TBR1 expression (McKenna et al., 2011). TBR1 expression is also downregulated directly by FOXG1 and indirectly by the FOXG1 mediated upregulation of FEZF2 (Toma et al., 2014). Moreover, SOX5 (Sex Determining Region Y-Box 5) directly represses FEZF2 by binding to a required enhancer element for its expression (Kwan et al., 2008; Lai et al., 2008; Shim et al., 2012) and induces the activation and maintenance of TBR1 (Bedogni et al., 2010).

As deep layer progenitors express OTX1 before neurogenesis, upper layer progenitors express the non-coding RNA SVET1. The expression of this marker is exclusive of layer II–IV and its activation is dependent on the correct expression of PAX6 (Tarabykin et al., 2001). Expression of SVET1 has been also identified in IPC-derived upper cortical layer neurons (Molyneaux et al., 2007).

Transition to the production of upper layer neurons is mediated by the interaction of FEZF2, CTIP2, TBR1, and SATB2 (Special AT-Rich Sequence-Binding Protein 2). TBR1 is a downstream target of SATB2, and its repression is necessary for the differentiation of layer IV neurons (Srinivasan et al., 2012). Moreover, downregulation of FEZF2 by negative feedback (Toma et al., 2014) is necessary for the acquisition of SATB2 expression (Chen et al., 2008), and in time, SATB2 binds to and represses the expression of CTIP2 (Alcamo et al., 2008; Britanova et al., 2008).

Co-expression of Brain-Specific Homeobox/POU Domain Protein 1 (BRN1) and Brain-Specific Homeobox/POU Domain Protein 2 (BRN2/POU3F2) in most layer II–V cortical neurons is preceded by its expression in VZ progenitors and they are required for the control of radial migration in neurons residing in those cortical layers (McEvilly et al., 2002; Dominguez et al., 2013). Moreover, disruption in the expression of these transcription factors causes defective migration of neurons due to the inability of the neurons to express Dab1 (Sugitani et al., 2002).

Expression of the transcription factor CUX2 in progenitors has been associated with the generation of cortical neurons from layers II/III (Nieto et al., 2004; Zimmer et al., 2004; Molyneaux et al., 2009) at post-conception week 12 (Clancy et al., 2001). This predisposition to generate upper layer neurons is observed in the progenitor cells in the VZ that undergo proliferative cell division during the phase of deep-layer neuronal generation. These cells then switch to neurogenic differentiation to generate superficial-layer neurons (Franco et al., 2012). CUX1 and CUX2 are also part of the regulatory network modulated by FEZF2, where its inactivation allows for the progression to upper layer specification (Lodato et al., 2014).

#### Gliogenesis

During cortical neurogenesis, the promoters for astrocytic fate, such as GFAP, are heavily methylated and inaccessible to be activated by STAT3 (Signal Transducer And Activator Of Transcription 3) (Takizawa et al., 2001). Expression of Neurogin 1 (NGN1) competes with STAT3 for the EP300-SMAD activator complexes and suppress the JAK/STAT and BMP signaling pathway (Sun et al., 2001). At the end of neurogenesis, levels of NGN1 drop and the GFAP promoter is demethylated to induce astrocytic fate and promote the differentiation of radial glia cells into glial progenitors (Takizawa et al., 2001). This demethylation of the astrocyte-specific genes is mediate by the Notch signaling pathway activation of the nuclear factor IA (NFIA) (Deneen et al., 2006; Namihira et al., 2009). NFIA is also directly regulated by SOX9 (Sex Determining Region Y-Box 9), with the SOX9/NFIA complex directly regulating genes implicated in astrocyte precursor migration and metabolism (Kang et al., 2012).

Astrocytes arise from radial glia in the VZ and PAX6+/HOPX+ oRGs in the outer SVZ (Cameron and Rakic, 1991; Noctor et al., 2008; Kriegstein and Alvarez-Buylla, 2009; Ge et al., 2012; Zweifel et al., 2018; Rash et al., 2019) around post-conception week 12 (Empie et al., 2015). Local proliferation of differentiated astrocytes that undergo symmetric division generate mature astrocytes (García-Marqués and López-Mascaraque, 2013) that are able to form endfeet with blood vessels and are perform functions such as glutamate uptake (Ge et al., 2012).

Another class of glial cells, oligodendrocytes, originate from precursors in the proliferative dorsal and ventral zones of the developing brain (Richardson et al., 2006). These cells migrate to the developing white matter, divide, and terminally differentiate. The activation of the transcription factors OLIG1 (Oligodendrocyte Transcription Factor 1) and OLIG2 (Oligodendrocyte Transcription Factor 2) are indispensable for the oligodendroglial fate acquisition. OLIG1 directly binds to DLX1/2 (Distal-Less Homeobox 1/2) enhancers to repress neurogenic fate and induce the expression of OLIG2 (Petryniak et al., 2007; Silbereis et al., 2014). ASCL1 (Achaete-Scute Family BHLH Transcription Factor 1) is also downregulated in the transition from neural progenitor cells (NPCs) to oligodendrocyte progenitor cell, although its expression in these cells is biphasic as it needs to be upregulated again for oligodendrocyte terminal differentiation (Battiste et al., 2007; Dimou et al., 2008; Sugimori et al., 2008). Expression of several SOX (Sex Determining Region Y-Box) members is key for the specification and differentiation of oligodendrocytes. SOX5 and SOX6 are expressed in oligodendrocyte progenitor cells and influence migration patterns, while SOX10 induces terminal differentiation and myelin gene expression (Stolt et al., 2002, 2006).

Sonic Hedgehog signaling, through the activation of OLIG2, is necessary for oligodendrocyte development (Agius et al., 2004); yet their maturation is SHH independent (Orentas et al., 1999; Soula et al., 2001). Expression of the EGF receptor has also been associated with the generation of OPCs (Huang et al., 2020). EGFR-expressing oRGs may act as intermediate progenitors for oligodendrogenesis and potentially amplify the OPC progenitor pool (Huang et al., 2020). BMP and WNT expression have been shown to oppose oligodendrocyte cell fate and directing the progenitors into an astrocyte fate (Mekki-Dauriac et al., 2002; Samanta and Kessler, 2004; Shimizu et al., 2005).

## Human Pluripotent Stem Cell Derived Models for the Study of Brain Development

Animal models (Jain et al., 2016, 2019; Ferrari et al., 2017) and brain tissue from biopsies have provided critical insight into mitochondrial disease. However, our understanding of the etiology and pathology of complex mitochondrial diseases would benefit from the use of human-derived platforms such as induced PSC-derived models described in this section. Human induced pluripotent stem cells (iPSCs) have been generated from patients with mutations in mitochondrially encoded ATP Synthase Membrane Subunit 6 (MT-ATP6) (Ma et al., 2015; Galera-Monge et al., 2016; Lorenz et al., 2017; Grace et al., 2019), mitochondrially encoded NADH:Ubiquinone Oxidoreductase Core Subunit 3 (MT-ND3) subunit (Hattori et al., 2016), and the nuclear-encoded gene Surfeit locus protein 1 (SURF1) (Inak et al., 2021). These iPSC-model systems are useful tools for drug discovery (Inak et al., 2017; Lorenz et al., 2017)as well as testing platforms for potential metabolic rescue treatments (Ma et al., 2015).

**FIGURE 2:**
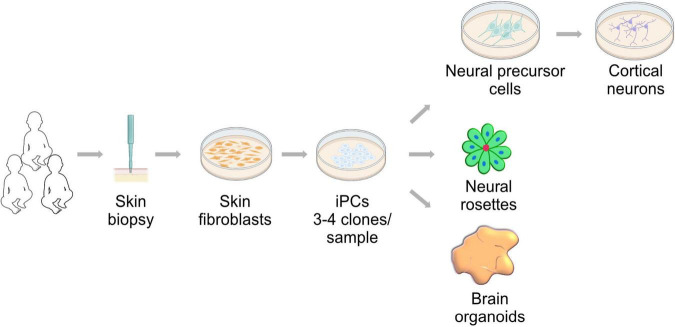
Neural development research approach utilizing induced pluripotent stem cells (iPSCs). Skin fibroblast can be derived from patients and controls by a minimally invasive biopsy. These fibroblasts can be reprogrammed using the Yamanaka factors into induced pluripotent stem cells. iPSCs can generate neural cells in two- and three-dimensional cultures. These approaches are powerful tools to study of neural development in health and mitochondrial disease conditions.

The study of human embryonic development described in the previous section, specifically organogenesis and cell fate specification, has been greatly improved by the isolation and maintenance of PSCs (Evans and Kaufman, 1981; Thomson et al., 1995, 1998; Thomson and Marshall, 1998). The access to somatic cells and the ability to reprogram them into induced PSCs (Takahashi and Yamanaka, 2006; Takahashi et al., 2007) has enabled modeling developmental diseases that are considered rare or difficult to phenocopy in model organisms, including mitochondrial diseases (Saha and Jaenisch, 2009). This, coupled with the ability to generate differentiated cells such as neurons (Zhang et al., 2001; Vierbuchen et al., 2010), has expanded the technical approaches available to study human brain development in vitro (Figure 2). Here we provide an overview of current iPSC-derived systems and the developmental insight we can gain from their use. These models could expand the repertoire available to understand the contribution of mitochondrial biology and function to human brain development and could expand our view of mitochondrial diseases.

### Human Pluripotent Stem Cell-Derived Neurons

The generation of neural cells was based on observations of the grafting effects of mouse teratocarcinoma lines in early embryos and in vitro (Jones-Villeneuve et al., 1983). Cell aggregation in addition to treatment with RA promoted the differentiation of mouse embryonic stem cells (mESCs) into cells expressing neuronal genes and capable of generating action potentials (Bain et al., 1995). The use of morphogens for the maintenance and generation of neuroepithelial precursors was highlighted by the use of basic fibroblast growth factor (bFGF or FGF2) (Okabe et al., 1996; Mujtaba et al., 1999). Removal of this compound promoted the differentiation of functional neuronal cells that were able to be cultured long term (Okabe et al., 1996). Furthermore, these ESCs-derived neural precursors were shown to be able to graft to embryonic ventricles and migrate without positional cues into the host brain to contribute to the three lineages of the nervous system (Brüstle et al., 1997).

The first account of human neural precursor cells differentiation from hESC was reported by the Thomson and Ben-Hur laboratories in 2001. After aggregation in embryoid bodies, neural tube-like structures were generated in the presence of FGF2 (Reubinoff et al., 2001; Zhang et al., 2001). Removal of this morphogen promoted differentiation into the neural lineages in vitro, while transplantation into the neonatal mouse brain allowed for their incorporation to different brain regions and further maturation into neurons and astrocytes.

An improved protocol that did not rely on embryoid bodies was generated by the Studer lab in 2009. Rapid and highly efficient neural conversion under adherent culture conditions was obtained by the synergistic use of two SMAD signaling pathway inhibitors: Noggin and SB431542 (Chambers et al., 2009). Noggin is a bone morphogenic protein (BMP) inhibitor identified initially in *Xenopus laevis* with neural-inducing properties following a dorsal fate pattern (Smith and Harland, 1992; Valenzuela et al., 1995; Gerrard et al., 2005; Lee et al., 2007). The drug SB431542 is an antagonist that inhibits the Nodal/Activin/Transforming growth factor β (TGFβ) pathways by blocking phosphorylation of the ALK4, ALK5 and ALK7 receptors. Activin and Nodal are responsible of mesodermal and endodermal differentiation during gastrulation, the inhibition of these pathways allows for an ectodermal and neural fate induction both *in vivo* and *in vitro* (Schier, 2003; Smith et al., 2008).

Neural progenitor cell fate is determined by the expression of neuroectoderm markers such as Sex Determining Region Y-Box 1 (SOX1), PAX6 and Neuroepithelial stem cell protein (NESTIN). The intermediate filament NESTIN is expressed in both neuroepithelial cells and RGCs with different morphologies depending on the cell type and size (Götz and Barde, 2005). Expression of SOX1 can be observed upon neuroectoderm formation. Subsequent expression of PAX6 is noted as early as the first somite formation and the closing of the neural fold in the cranial region (E8.0 in mouse and stage 10 in human). PAX6 is also expressed in RGCs (Callaerts et al., 1997; Englund, 2005; Thakurela et al., 2016). The switch between SOX1 and PAX6 during neural fate commitment is required for the correct differentiation into committed cell types. Continuous expression of SOX1 inhibits the expression of PAX6 and other RGC markers. PAX6 expression induces cell migration and differentiation into neurons (Heins et al., 2002; Cartier et al., 2006; Suter et al., 2009).

Further differentiation to different neuronal cell types from different regions of the CNS have been achieved (Pankratz et al., 2007; Zeng et al., 2010). Previous studies using mice and other model organisms expanded the knowledge on morphogens and growth factors required for neural development (Gaspard et al., 2008). NPCs with anterior patterning fate have been used to generate dopaminergic neurons after exposure to FGF8 for anterior–posterior axis induction and signal SHH as a ventralizing signal (Perrier et al., 2004). Spinal motoneurons can be generated by the presence of SHH and the retinoic acid to promote the posterior axis fate (Li et al., 2005; Lee et al., 2007). Functional cortical glutamatergic neurons and telencephalic GABAergic neurons can also be generated by the manipulation of the endogenous WNT signaling in NPCs (Li et al., 2009). Cerebral cortex development can also be recapitulated by the sequential generation of pyramidal neurons that express the markers of the different cortical layers (Shi et al., 2012; Espuny-Camacho et al., 2013).

As an alternative to hPSC-derived neurons, direct differentiation from somatic cells into functional NPCs and neurons have been achieved, using either genetic or chemical manipulation of the embryonic pathways that promote transdifferentiation. The transcription factors BRN2, ASCL1, Myelin Transcription Factor 1-Like Protein (MYTL1), and Neurogenic differentiation 1 (NEUROD1); as well as the microRNAs miR-9* and miR-124, were initially used to generate different types of neurons from fibroblasts (Ambasudhan et al., 2011; Caiazzo et al., 2011; Pang et al., 2011; Pfisterer et al., 2011a,b; Son et al., 2011; Yoo et al., 2011). Single transcription factors, such as Sex Determining Region Y-Box 2 (SOX2) (Ring et al., 2012) and NEUROGENIN-2 (NGN2) (Zhang et al., 2013) have been used to generate functional excitatory cortical neurons.

### Human Pluripotent Stem Cell-Derived Neural Rosettes

The earliest *in vitro* recapitulation of nervous system development are neural rosettes (NR). These structures correspond to the third week of gestation (Chandrasekaran et al., 2017) and recapitulate the initial stages of CNS development. NRs are composed of long, radially organized, columnar neuroepithelial cells surrounding a central cavity or lumen (Zhang et al., 2001; Elkabetz et al., 2008; Chandrasekaran et al., 2017; Hříbková et al., 2018). NRs present apical-basal polarity, with the localization of apical markers (e.g., ZO1, *N*-Cadherin, β-Catenin) and interkinetic nuclear migration. Additionally, cells in NRs are positive for neuroepithelial markers such as PAX6, SOX1, NESTIN, Mushahi-1 (MSI1) and polysialylated neuronal cell adhesion molecule (PSA-NCAM). These cells also show self-renewal capacity, engraftment capacity, and can differentiate into different region-specific neural and glial types in response to developmental cues (Zhang et al., 2001; Perrier et al., 2004; Li et al., 2005; Elkabetz et al., 2008; Koch et al., 2009; Grabiec et al., 2016; Knight et al., 2018).

Neural rosettes-neural stem cells (NR-NSCs) can be isolated, expanded, and regionally specified without losing the rosette properties. Maintenance of the multipotency capacity has been shown to depend on the Notch signaling pathway, as low plating densities or the pharmacological inhibition of the pathway, increases the neuronal differentiation and causes a reduction in the rosette formation efficiency. Activation of the SHH has also been shown to be a key pathway in the maintenance of multipotency of NR-NSCs. Inhibition of both pathways cause rapid loss of rosette organization and reduction in the differentiation capacity (Elkabetz et al., 2008).

Although the default fate pathway for NR cells is the anterior CNS pattern by the expression of FOXG1 (Tao and Lai, 1992; Koch et al., 2009), re-specification toward caudal fates can be accomplished: midbrain and hindbrain neurons can be obtained by FGF8/SHH treatments, and spinal motor neurons can be generated by incubation in RA/SHH (Perrier et al., 2004; Elkabetz et al., 2008). Furthermore, NRs with ZO1 expression and interkinetic nuclear migration can also be spontaneously generated from NPC monolayer differentiation via dual SMAD inhibition (Chambers et al., 2009).

Neural rosette formation consists of five morphogenetic changes: intercalation of two or more cell rows, cellular constriction or shrinkage, polarization, elongation, and lumen formation. The formation of the lumen is mediated by apoptosis, inhibition of cell death via caspase inhibition disrupts lumen formation and delay neurogenesis. Ca2+ regulation is also critical in the formation of the NR by regulating multiple cytoskeletal proteins during the first three morphogenesis events. Calcium dysregulation also affects the localization of the polarizing proteins ZO1, PARD3 and β-catenin, which in turn impairs lumen formation. Disruption of the cytoskeleton (specifically actin, myosin II and tubulin) promotes premature neurogenesis and astrogenesis (Hříbková et al., 2018).

FGF and BMP signaling are also necessary for the correct formation of the NRs. FGF2 and its receptor FGFR1 present an apical polarization in the lumen of the NR. FGF2 overexpression or inhibition disrupts the formation of the NRs and affects ZO1 expression and localization. The malformation of the NRs can be mediated by the displacement of ZO1 from the apical membrane which in time disrupts the membrane anchorage of FGFR1, causing the disruption on FGF2 signaling gradient, reduced cell proliferation, cell cycle exit and the premature differentiation of the NPCs (Grabiec et al., 2016). BMP signaling has been associated to the complex cluster formation of the NR. Inhibition of the pathway causes disrupted rosette morphology and alters the expression of the NSC markers PAX6, SOX2, and SOX1 (Fedorova et al., 2019).

### Human Pluripotent Stem Cell-Derived Brain Organoids

The brain organoid field started with the groundbreaking observations of the Yoshiki Sasai group. These initial studies demonstrated the ability of mouse ESCs to directly differentiate into telencephalic precursors in a serum-free suspension culture (SFEB culture). Treatments with WNT and Nodal antagonist resulted in a high yield of PAX6+ cells that could be further differentiated into ventral or dorsal fate after WNT3a or SHH, respectively (Watanabe et al., 2005). Optimization of the SFEB culture media by the inclusion of a BMP inhibitor and the introduction of Y-27632, the selective Rho-associated kinase (ROCK) inhibitor, allowed to translate these findings to human derived systems (Watanabe et al., 2007).

The derivation of optimized mouse and human culture media allowed for the generation of three-dimensional aggregates that recapitulate embryonic corticogenesis and regional specification (Eiraku et al., 2008). Polarized cortical neuroepithelia resembling neural rosettes positive for the expected markers were observed in the floating aggregates. Moreover, cortical specification was obtained, mimicking early corticogenesis with the segregation of discrete layers containing radial glia, neuronal progenitors, and early neurons (Eiraku et al., 2008; Mariani et al., 2012; Kadoshima et al., 2013). Additionally, gene expression profile in these aggregates correlate with the embryonic telencephalon, specifically to the transcriptional program active at 8–10 weeks after conception (Mariani et al., 2012).

Cerebral organoids can be generated via undirected or directed differentiation. The undirected differentiation technique was described by the Knoblich group in 2013 (Lancaster et al., 2013). Single cell hPSCs were aggregated in a serum free media and then embedded in an extracellular matrix (ECM). The presence of ECM supports the formation of neuroepithelial buds that expand into cortical structures under constant agitation (Lancaster et al., 2013; Lancaster and Knoblich, 2014; Renner et al., 2017). The lack of external signaling to induce patterning allows for the formation of organoids with various brain region identities and non-neural derivatives (Camp et al., 2015; Quadrato et al., 2017). Although variable, undirected organoids allow for a high degree of diversity in the cultures that serve as a platform to explore the CNS diversity and the effects of diseases onto different cell types (Lancaster et al., 2017; Paşca, 2018; Kanton et al., 2019; Velasco et al., 2019).

Directed differentiation uses small molecules to induce regional specification (Kadoshima et al., 2013). The absence of ECM, as well as static conditions, allows for the formation of spherical cultures that can be further differentiated into dorsal and ventral aggregates (Paşca et al., 2015; Birey et al., 2017; Sloan et al., 2018), enriched with astrocytes (Sloan et al., 2017) or oligodendrocytes (Marton et al., 2019). Long term culture of these spheroids allows for the continuous maturation and differentiation of the structures allowing for the exploration of brain development in mid-fetal stages (Paşca et al., 2015; Sloan et al., 2017; Yoon et al., 2019; Gordon et al., 2021). Regional patterning can also be achieved using shaking conditions such as miniaturized bioreactors to generate forebrain, midbrain and hypothalamus organoids (Qian et al., 2016, 2018).

#### Challenges and Limitations of Induced Pluripotent Stem Cells Models

Although a remarkable system to study neural development, iPSC-derived models present a set of limitations: cellular variability, lack of maturation, and limited reproduction of the brain cellular complexity (Dolmetsch and Geschwind, 2011; Volpato and Webber, 2020; Yamanaka, 2020; McTague et al., 2021; Qian and Tcw, 2021). These limitations must be considered when translating findings to human development.

The generation of NPCs relied on the differentiation of the hPSCs into neuroectodermal fate (Dhara and Stice, 2008; Chambers et al., 2009), which continues into a preferential acquisition of dorsal forebrain identity (Watanabe et al., 2005; Nat et al., 2007; Zhang M. et al., 2018). Supplementation of exogenous SHH is necessary for a ventral fate acquisition (Ribes and Briscoe, 2009; Liu et al., 2013; Maroof et al., 2013; Nicholas et al., 2013; Tao and Zhang, 2016), as the default dorsal identity is partially due to NPC expression of WNT ligands (Li et al., 2009). Moreover, variability in the capacity of the cells to generate NPCs based on its embryonic or induced origin has been reported (Koyanagi-Aoi et al., 2013), but a more in depth proteomic and genomic characterization has not been done.

Similarities of iPSC-derived cortical neurons to primary cortical neurons have been established through single cell analysis but, layer-specific subtype characterization remains challenging (Handel et al., 2016). NPC cultures are heterogeneous, with mixed populations of neural stem and progenitor cells during the first stages of differentiation. At later stages, combinations of neuron and astrocyte populations can be obtained in an stochastic manner (Hoffman et al., 2017, 2019). This can affect not only the functionality of each subtype but their maturation stage (Brennand et al., 2015). A cleaner, more mature neuronal population can be obtained using direct differentiation methods (Mertens et al., 2018; Meijer et al., 2019; Rhee et al., 2019), yet transfection and selection stress can influence the quality of the cell types that are generated.

Generation of astrocytes and oligodendrocytes is also challenging. Glial cell types appear later in development and require changes in the developmental cues. Current protocols, especially for oligodendrocyte generation, require the use of multiple small molecules and morphogens as well as month-long maintenance to generate cell populations suitable for experimentation (Douvaras et al., 2014; Douvaras and Fossati, 2015; Tcw et al., 2017).

Due to the lack of vascularization, brain organoids display limited growth (Lancaster et al., 2013). Brain organoids generate neurons positive for the six different cortical layers, but layer organization as well as axonal projection patterns lack the expansion seen in similar developmental stages. Moreover, certain structures that are clearly delimitated in the developing brain -such as the subplate and the cortical plate- are difficult to differentiate (Qian et al., 2019). Likewise, intrinsic generation of non-neural cell types such as microglia, vascular cells, and immune cells; requires co-culturing conditions.

Another limitation of the brain organoid system is the inability to recapitulate developmental gradients. Fusion of regional aggregates has been useful to study neural migratory patterns and complex interregional interactions (Bagley et al., 2017; Birey et al., 2017; Xiang et al., 2017, 2019; Miura et al., 2020), but this approach requires the individual generation of the brain regions. The main hurdle to overcome is the correct localization and concentration of morphogens. A pioneering study by the Studer laboratory generated a SHH ventralizing gradient by utilizing a two-step EB formation process (Cederquist et al., 2019). First, a doxycycline-inducible hPSC line capable of expression of the morphogen was used. Sequentially, additional hPSCs were seeded around the SHH signaling EB, creating an organizing center. This approach successfully produces telencephalic organoids with a dorsal-ventral axis. Organoid-on-chip approaches are also being explored. Morphogen-soaked beads positioned near developing cerebral organoids have shown the effects of WNT and BMP4 gradients (Ben-Reuven and Reiner, 2020). WNT inhibition and BMP4 induction generated changes in the transcriptional profile of the areas proximal and distal to the bead, in a concentration dependent manner. These results suggest that recapitulating developmental morphogen gradients may require a combined approach between engineering and developmental biology. The brain organoids system recapitulates some aspects of human CNS development and complementation with other model systems and approaches can expand their capability and potential.

## Mitochondrial Homeostasis and Neural Development

Mitochondria are ubiquitous and essential organelles for cell survival. Known primarily for their capacity to generate energy via oxidative phosphorylation (OXPHOS) and their role in cell death, mitochondria also act as a signaling hub and coordination center for a myriad of cellular processes including metabolite synthesis and calcium buffering. Mitochondrial network remodeling, through fusion and fission, is necessary for discarding damaged or not functional mitochondria (mitophagy), to adapt to new energetic requirements, and to redistribute the organelle throughout the cytoplasm (motility) (Anesti and Scorrano, 2006; Chan, 2012; Cogliati et al., 2013; Schwarz, 2013; Barnhart, 2016; Giacomello et al., 2020). Studies on the involvement of mitochondrial function and dynamics in neurogenesis have been limited by the availability of model systems. The advent of human iPSC-derived systems has opened the possibility to examine the contribution of mitochondrial dynamics, morphology, and function, to cortical development.

### Mitochondria Form and Function

#### Bioenergetics

The energy producing machinery of the mitochondria is located in the cristae, invaginations of the inner mitochondrial membrane that increase the surface area (Cogliati et al., 2016). The proteins responsible for OXPHOS comprise four different complexes that assemble further into supercomplexes (Schägger and Pfeiffer, 2001; Dudkina et al., 2005; Acín-Pérez et al., 2008). These complexes couple the oxidation of reducing molecules, such as NADH and FADH2, to the translocation of protons across the inner membrane. This influx of protons generates a proton electrochemical gradient that is used by the ATP synthase to generate ATP from ADP.

All five complexes are localized along the cristae membrane with an overlapping distribution (Wilkens et al., 2013). The formation of supercomplexes optimizes electron transport and proton shuttling through the inner membrane (Acín-Pérez et al., 2008; Letts and Sazanov, 2017). ATP synthase is localized on the edges of the cristae forming dimers, with the other complexes located along both sides (Davies et al., 2011). This configuration seems to be fundamental for the creation of the proton gradient that accumulates in the concave side of the cristae (Strauss et al., 2008; Rieger et al., 2014).

The correct formation and maturation of the cristae is considered a hallmark of cellular maturation and differentiation. PSCs have fragmented mitochondria with immature cristae (Rafalski et al., 2012) and while oxidative phosphorylation still occurs, they rely preferentially on glycolysis for energy production (Piccoli et al., 2005; Chung et al., 2010; Prigione et al., 2010; Zhang et al., 2011). The differentiation of stem cells into neural stem cells is coupled to metabolic shifts that are essential. Inability to transition from glycolysis to OXPHOS during neural induction causes cell death in ESCs and iPSCs (Zheng et al., 2016; Beckervordersandforth et al., 2017), and inhibition of mitochondrial function blocks neural differentiation and promotes pluripotency (Pereira et al., 2013).

The glycolysis-OXPHOS modulation impacts neurogenesis at different stages. Disruption of OXPHOS-related genes in *Drosophila* inhibits the cell cycle exit and promotes proliferation (Homem et al., 2014; van den Ameele and Brand, 2019). Dysregulation of the mitochondrial complex I function has severe impacts in the capacity of NPCs to proliferate, differentiate into mature neural and oligodendrocytic lineages (Cabello-Rivera et al., 2019). Proliferating intermediate progenitor cells rely on OXPHOS as its main source of energy by downregulation of key glycolytic enzymes and upregulation of enzymes from the TCA cycle and the mitochondrial supercomplexes (Beckervordersandforth et al., 2017).

Although reduced, glycolysis is vital for the NPC population during development. For example, methylglyoxal, a byproduct of glycolysis, influences NPC self-renewal by binding to GAPDH and modulating the translational control of Notch1 (Rodrigues et al., 2020). Other pathways such as glutaminolysis, process by which glutamine is converted into TCA cycle metabolites, have been also associated with cortical expansion and neurogenesis (Rosenberg et al., 2002; Lindhurst et al., 2006; Journiac et al., 2020; Namba et al., 2020).

The mitochondria are also signaling organelles central to the production of TCA cycle metabolites, such as citrate and oxaloacetate, that can then generate macromolecules including lipids and nucleotides (DeBerardinis and Chandel, 2020; Martínez-Reyes and Chandel, 2020; Chakrabarty and Chandel, 2021) and downstream contribute to histone changes required for neurogenesis (Yang et al., 2019; Shan et al., 2020). Fatty acid metabolism is another aspect related to mitochondrial function that affects neurogenesis. Gain-of-function mutations in the enzyme fatty acid synthase causes reduced proliferation of NPCs in human-derived cerebral organoids (Bowers et al., 2020). Mitochondrial fatty acid β-oxidation (FAO) is also implicated in embryonic neurogenesis. FAO downregulation in mouse cortex decrease self-renewal and apoptosis of NPCs and suggest a key role for FAO in the NPC to IPC transition (Xie et al., 2016). Increase in FAO due to mitochondrial damage can also impair progenitor maturation in mouse neuroblasts (Audano et al., 2019) and striatal progenitors (Warren et al., 2017).

The mitochondria also mediate the release of signaling molecules from the mitochondria (e.g., acetyl-coA, cytochrome c, and free calcium) that control cell fate and function (Martínez-Reyes and Chandel, 2020). Endogenous generation of reactive oxygen species (ROS) in the mitochondria has been shown to promote neurogenesis via NRF2 and Notch pathway inhibition (Khacho et al., 2016), whereas ROS regulation in the developing forebrain via the mitochondrial uncoupling protein 2 (UCP2) is required to induce differentiation of the progenitor pool (Ji et al., 2017). REDOX (reduction-oxidation) balance has been implicated in different aspects of differentiation and neuronal fate acquisition, particularly via Sirt1 and chromatin remodeling (Prozorovski et al., 2008; Tiberi et al., 2012; Bonnefont et al., 2019).

These aspects of mitochondrial signaling beyond ATP production are certainly crucial during the cellular transitions that underlie human brain development. Brain organoids provide a useful tool to gain a mechanistic understanding of the involvement of these unique mitochondrial signaling pathways in human neurogenesis.

#### Mitochondrial Dynamics and Remodeling

The active remodeling of the mitochondrial network is crucial for the homeostatic and metabolic adaptation of the cell. Mitochondria are highly dynamic organelles that undergo fission and fusion event according to the cellular and environmental necessities. These highly conserved processes are regulated by large dynamin-related GTPases (Chen and Chan, 2004; Praefcke and McMahon, 2004; Hoppins et al., 2007; Chan, 2012).

Mitochondrial fission or fragmentation is executed by the highly conserved protein Dynamin-related protein 1 (DRP1) (Otsuga et al., 1998; Smirnova et al., 1998; Labrousse et al., 1999). Activation of DRP1, by multiple post-translational modifications, is required for its function at the outer mitochondrial membrane (Taguchi et al., 2007; Han et al., 2008; Chang and Blackstone, 2010; Chou et al., 2012; Prieto et al., 2016). At this site, DRP1 binds to adaptors located at the outer mitochondrial membrane. Mitochondrial fission 1 (FIS1), mitochondrial fission factor (MFF), and mitochondrial dynamics protein 49/51 (MID49/MID51) have been shown to be involved in DRP1 mediated fission (Mozdy et al., 2000; Tieu et al., 2002; James et al., 2003; Griffin et al., 2005; Gandre-Babbe and Van Der Bliek, 2008; Otera et al., 2010; Palmer et al., 2013; Shen et al., 2014; Liu and Chan, 2015; Osellame et al., 2016). DRP1 self-assembles in rings around the mitochondrial membranes where undergoes conformational changes mediated by GTP hydrolysis and constricts the organelle until it divides (Ingerman et al., 2005; Mears et al., 2011).

Mitochondrial fusion occurs when the outer and inner mitochondrial membrane merge with the corresponding membranes on another mitochondrion. Both fusion events are coordinated and occur simultaneously, resulting in the mixing of the mitochondrial contents in the matrix, membranes and intermembrane space and the homogenization of the mitochondrial DNA (mtDNA) and the formation and assembly of the electron transport chain (ETC) (Chan, 2012). Although coordinated, the fusion of the membranes is directed by distinct mechanisms. Mitofusin 1 (MFN1) and Mitofusin 2 (MFN2) are the proteins responsible for the outer membrane fusion, while optic atrophy 1 (OPA1) mediates inner membrane fusion (Alexander et al., 2000; Delettre et al., 2000; Santel and Fuller, 2001; Rojo et al., 2002; Chen et al., 2003). MFN1 and 2 can homo- or hetero-dimerize with mitofusins in the adjacent mitochondria. OPA1 have two proteolytically cleaved proteins: long OPA1 (OPA1-L) and short OPA1 (OPA1-S). OPA1-L is anchored in the inner mitochondria membrane and coordinated the fusion process by forming homodimers with the opposite target membrane. OPA1-S has been associated with the stabilization of the mitochondrial cristae, maintenance of the mtDNA content and energetic competence (Mishra et al., 2014; Del Dotto et al., 2017).

Neurodevelopmental studies have shown mitochondrial dynamics are essential in neurogenesis. Mouse models deficient in DRP1 show smaller brain size and reduced developmental apoptosis in the neural tube (Wakabayashi et al., 2009; Lewis et al., 2018); as well as high mortality of newborn deep layer neurons (Ishihara et al., 2009). Although rare, mutations in DRP1 have been identified in patients with severe neurodevelopmental delays (Baum and Gama, 2021). Mutations in the adaptor proteins MFF and MID49 have also been associated with developmental delay, myopathies, and neuropathies (Shamseldin et al., 2012; Koch et al., 2016; Bartsakoulia et al., 2018; Baum and Gama, 2021). Mutations in MFNs and OPA1 have also identified as the causal mechanism behind neurodegenerative diseases such as Charcot-Marie-Tooth syndrome, autosomal dominant optic atrophy, spastic paraplegia, syndromic Parkinson and dementia (Züchner et al., 2004; Alavi et al., 2007; Verny et al., 2008; Yu-Wai-Man et al., 2010; Carelli et al., 2015).

Studies in mouse models have shown significant cristae structural changes at the end of neural tube closure, as cells progress into neural progenitor stage. This modification in the mitochondria morphology correlates with the transition from a highly glycolytic metabolism to an OXPHOS dependent one (Fame et al., 2019). This switch in the mitochondrial structure, and the subsequent energetic requirements, are dependent on the downregulation of MYC, which has been shown to be a key regulator of ribosomal biogenesis, protein synthesis, and cellular proliferation at this stage (Chau et al., 2018). In vitro differentiation of mouse cortical neurons causes changes in mitochondrial mass due to increase mitochondrial biogenesis mediated by upregulation of mitochondrial transcription factor A (TFAM) and peroxisomal proliferating activating receptor γ coactivator-1α (PGC-1α) (Agostini et al., 2016). Glycolysis-to-OXPHOS transition was also observed during neuronal differentiation.

Disruption of mitochondrial fission and fusion proteins has been shown to result in both neurodevelopmental and neurodegenerative disease, both age-associated and progressive (Khacho and Slack, 2018). Mitochondrial dysfunction and aberrant mitochondria morphology are hallmarks of impaired brain development in both animal and human derived models (Waterham et al., 2007; Ishihara et al., 2009; Wakabayashi et al., 2009; Fang et al., 2016; Spiegel et al., 2016).

The regulation of mitochondrial dynamics and its physiological relevance in the context of tissue architecture is still an unexplored landscape (Liesa and Shirihai, 2013; Noguchi and Kasahara, 2017). Studies of the mitochondrial morphology in adult mouse brain have shown that different cellular stages of differentiation possess a distinct mitochondrial morphology. Adult hippocampal radial glia-like NSCs have mixed globular and tubular shape mitochondria, while IPCs have more thin and more elongated network and adult neurons featured a wider and highly elongated morphology (Beckervordersandforth et al., 2017). Neurons also have higher mitochondrial volume, which can be correlated with an increase in bioenergetics mediated by the ETC and OXPHOS activity (Bélanger et al., 2011).

In the developing mouse brain, Khacho et al. (2016) described the morphology of the mitochondria at different cortical compartments. Neural stem cells in the VZ, positive for the marker SOX2, presented an elongated mitochondrial network; while IPC, stained with the marker TBR2, in the SVZ have a fragmented morphology. Newly committed neurons expressing TUJ1 (βIII-tubulin), regained an elongated mitochondria structure in the cortical plate. Deletion of OPA1 and MFN1/2, GTPases that mediate mitochondrial membrane fusion, impaired neural stem cell self-renewal and promote early differentiation as a result of the sustained mitochondrial fragmentation. Promotion of highly fused mitochondria by manipulation of DRP-1, the main effector of mitochondrial fission, had the opposite effect by increasing the ability of neural stem cells to self-renew.

In a follow up study, Iwata et al. (2020) analyzed the changes of the mitochondrial network through the early stages of neurogenesis in 2D cultures of mouse and human neural cells. From their results, PAX6+ RGC displayed fused mitochondria and TBR2+ IPCs had an intermediate mitochondrial size. In their case, βIII-tubulin expressing neurons had a fragmented mitochondrion. Interestingly, they showed that the cell fate specification occurs during a restricted time window during the postmitotic period where daughter cells inheriting fragmented mitochondria differentiate and daughter cells inheriting a fused network will retain the capacity to self-renew.

Although we are beginning to elucidate the role of mitochondria morphology and homeostasis during neuronal specification, little is known in human models about the role that these organelles play during gliogenesis. Astrocytes rely on glycolysis more than OXPHOS for their energy production, specially by the utilization of fatty-acids as source of fuel (Eraso-Pichot et al., 2018; Fecher et al., 2019; White et al., 2020). Moreover, developing astrocytes contain a highly interconnected functional network of mitochondria and upregulation of the mitochondrial network is crucial for coordinating post-natal astrocyte maturation and synaptogenesis (Zehnder et al., 2021).

Oligodendrocytes, on the other hand, rely on OXPHOS during the progenitor stage and switch to glycolysis when mature (Rinholm et al., 2011; Fünfschilling et al., 2012; Amaral et al., 2016; Rao et al., 2017; Fecher et al., 2019). Mitochondrial fragmentation is key in mature oligodendrocytes as myelin sheets contained smaller mitochondria if compared to the network surrounding the nucleus (Rinholm et al., 2016; Battefeld et al., 2019). Yet, the effects of the mitochondrial dynamics during their development and specification remains unknown.

### Mitochondria Morphology Changes Through Neural Differentiation and Maturation

Due to the highly dynamic nature of mitochondria, analysis of its morphology has been challenging in *in vivo* and 3-D settings. Most of the existing classification have resorted to manual and morphological classification (e.g., fused vs. fragmented) of the mitochondrial networks, utilizing qualitative or semi-quantitative approaches (Rafelski, 2013; Prieto et al., 2016; Noguchi and Kasahara, 2017; Faitg et al., 2021; Fogo et al., 2021). Advances in imaging techniques coupled with computational approaches have improved the capacity to unbiasedly and consistently assess the morphology of these organelles (Leonard et al., 2015; Zahedi et al., 2018). Recently, machine learning algorithms (Kan, 2017) together with genetic perturbations of key mitochondrial players, have been proposed as a potential alternative to not only evaluate the phenotypical aspects of the mitochondria but to assess the physiological relevance of those changes (Fogo et al., 2021).

A comprehensive study of the mitochondrial network of different cell types during early neurogenesis is required. In accordance with previous findings in the mouse brain and *in vitro* neurons (Khacho et al., 2016; Iwata et al., 2020), NPCs that are positive for the transcription factors PAX6 and SOX2 show an elongated mitochondrial network. In contrast, committed neurons positive for the cytoskeletal marker βIII-tubulin have a fragmented network. While this characterization is semi-quantitative, recent tools using machine learning have been developed that can be coupled to refine the analysis of the mitochondrial morphologies in different cell types (Berg et al., 2019). This analysis could make it possible to compare the changes observed in mouse brains and 2-D hPSC-derived neurons to a 3-D model of development and shed light into the differences and similarities of the different research models. This new method of mitochondrial scoring and quantification can be extended to other neural organoid protocols. For example, dorsal and ventral spheroids (Birey et al., 2017) or thalamic organoids (Xiang et al., 2019) can be used to explore the effects of mitochondrial and mitochondrial associated mutations in the migration of axons. This would be of particular interest in disease models where can be axonal migration can be affected or where proper formation of axonal tract play a crucial role in the pathophysiology of the disease (Giandomenico et al., 2019; Kitahara et al., 2020). These techniques can be also used for the characterization of the mitochondrial morphology and the network regulation in other tissues during development and disease. Organoids that mimic highly metabolic tissues such as cardiac muscle, kidney and liver could be used to understand the effects of the mitochondrial dynamics under homeostatic and allostatic conditions.

## Mitochondrial Fitness in Neurogenesis and Disease

Mitochondrial function is central to the homeostasis of highly metabolic tissues. The brain is responsible of consuming nearly 20% of the oxygen and calories from the body, while representing about 2% of its total weight (Raichle and Gusnard, 2002; Picard and McEwen, 2014). Although mitochondrial dysfunction cause by mutations in mitochondrial or metabolic genes results in severe multisystemic disease, the brain is more vulnerable to these defects in mitochondrial function. Thus, mitochondrial health sustains the functional and structural plasticity of the CNS.

The exact mechanisms underlying the regulation of mitochondrial dynamics during human neural development have remained widely unexplored, as most studies have been done in yeast, cultured mammalian cells, and mice (Liesa and Shirihai, 2013; Noguchi and Kasahara, 2017). As mentioned previously the known differences between human and mouse brains (Arbour et al., 2008; Pressler and Auvin, 2013; Khacho et al., 2016; Khacho and Slack, 2018) make studies in human models imperative. Whether mitochondrial function in bioenergetics, calcium handling, ROSs production, and other signaling events, differ among human neural populations and what is the contribution of mitochondrial fitness during the neuronal specification, migration, synaptic transmission, and cognition, could be revealed using human models (Ioannou et al., 2019).

**FIGURE 3:**
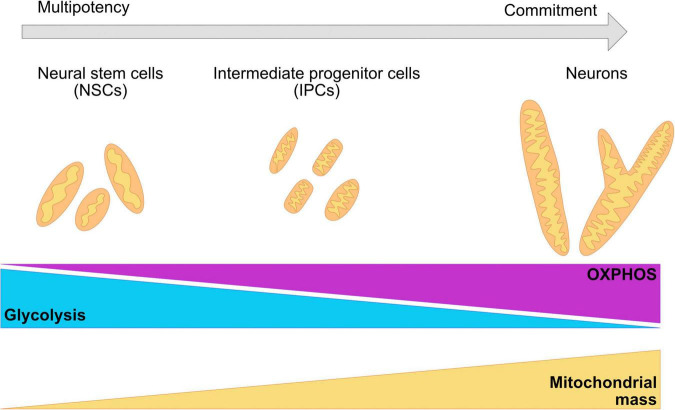
Changes in the morphology of the mitochondrial network are required for the commitment of neuronal fate. During neurogenesis, the mitochondrial network undergoes crucial remodeling to adapt to the bioenergetic necessities of the cell, as well as the requirements of the environment. NSC have been shown to present a mildly elongated mitochondrial network with a mix of globular and tubular mitochondrion. IPCs cells are characterized for fragmented, thin, and elongated networks. Committed neurons have a wider and elongated mitochondrial network. A metabolic switch from glycolysis to OXPHOS is necessary for the acquisition of the neuronal fate and it is associated with remodeling of the mitochondrial cristae, as well as with the increase of the number of mitochondria and mitochondrial mass.

Remodeling of the mitochondrial network as cells commit to a neuronal cell fate is crucial for survival and function (Figure 3; Chan, 2012; Schwarz, 2013; Khacho et al., 2016; Khacho and Slack, 2018; Iwata et al., 2020). Landmark studies demonstrate that modulation of mitochondrial dynamics during a post mitotic period can change the number of NPCs or neurons that are being produced in both mouse brains and hESC derived neurons (Hara et al., 2014; Iwata et al., 2020). In murine models, the mitochondrial network transitions from elongated structures in neural stem cells to fragmented mitochondria in intermediate progenitor cells and back to elongated structures in mature neurons (Khacho and Slack, 2018; Iwata et al., 2020). It is currently not clear whether these dynamic changes in mitochondrial shape are maintained in the human brain and their involvement in maintaining the metabolic profile of the neurons at different stages of differentiation and maturation.

Revealing the molecular underpinnings of mitochondrial form and function during the early stages of neurogenesis is fundamental to developing therapies that may control human disease. Coupling human brain organoids to super resolution microscopy, optogenetic approaches, gene editing and other technical approaches could uncover the role of mitochondria in the regulation of neurogenesis, synaptic transmission, brain function, and cognition. In the next sections, we provide an overview of recent advances in understanding the intricate relationship between mitochondrial function and brain development that have result from using human iPSC brain models.

The ability of studying mitochondrial fitness during early development could allow for the understanding of other neurodevelopmental disease caused by environmental factors and maternal health. The organoid system could be used to determine the effects of metabolic stress and nutrient imbalance in the developing brain as maternal metabolic diseases has been shown to correlate with increased risk of neurodevelopmental and psychiatric diseases in both human and animal studies (Shook et al., 2020; Edlow, 2021). Also, the capacity to recapitulate the formation of the neural tube as neural rosettes or as neuruloids -self-organizing structures containing neural progenitors, neural crest, sensory placode and epidermis- (Haremaki et al., 2019) could allow for the exploration of the molecular and cellular mechanisms behind complex CNS birth abnormalities.

### Mutations Associated With Neurodevelopmental Diseases Disrupt Mitochondrial Morphology and Function in Cerebral Organoids

Inborn errors of metabolism are rare genetic disorders resulting from defects in metabolic pathways (Das et al., 2010; Agana et al., 2018). Mitochondrial diseases are the most common group of inherited metabolic disorders and are among the most common forms of inherited neurological disorders (Gorman et al., 2016). These illnesses involve multiple organ systems and have limited therapeutic options (Parikh et al., 2017; Grier et al., 2018; Schaefer et al., 2019).

Leigh syndrome (LS) is one of these rare inherited neurometabolic diseases. Mutations in more than 75 genes associated with ATP production have been identified as causal, both in nuclear and mitochondrial DNA. It affects mostly infants within their first year of life and has a poor prognosis and a low survival expectancy (Finsterer, 2008; Lake et al., 2016). It is characterized by abnormal motor findings, epileptic seizures, increased lactate in the blood and cerebrospinal fluid, failure to thrive, and focal, bilaterally symmetrical necrotic lesions in the brain (Sofou et al., 2014, 2018). As it is a highly heterogeneous disease, the establishment of animal and in vitro models has been challenging and limited to only select mutations.

Animal models have been used to test therapeutic approaches, with mixed results. Gene editing using adeno-associated virus in *Ndufs4*–/– mice has shown partial rescue of the phenotype (Di Meo et al., 2017). Supplementation of nicotinamide riboside to *Sco2*–/– mice showed improvement of the respiratory chain defect and increased exercise tolerance due to improved mitochondrial biogenesis (Cerutti et al., 2014). Hypoxia and low oxygen availability in the brain have also been shown to increase the life span and improve neurological findings in *Ndufs4*–/– mice (Jain et al., 2016, 2019; Ferrari et al., 2017).

Reprogramming of patient fibroblast harboring nuclear and mitochondrial mutations (Galera-Monge et al., 2016; Zurita-Díaz et al., 2016; Grace et al., 2019; Romero-Morales et al., 2020; Inak et al., 2021; Meshrkey et al., 2021) has been used to generate specialized cells for the study of the impact of LS-associated mutations in highly metabolic tissues. Human iPSC models have been proposed as platforms to test new therapeutic approaches such as somatic nuclear transfer (Ma et al., 2015). Direct reprogramming of fibroblasts into neurons has been used to overcome the effects of heteroplasmy during reprogramming and as an alternative for targeted high-throughput drug screening and advancing precision medicine (Villanueva-Paz et al., 2019; Villalón-García et al., 2020).

These approaches have proven useful to investigate the effects of LS causing mutations in early neural development (Romero-Morales et al., 2020; Inak et al., 2021). Inak et al. (2021) generated iPSCs with mutations in the complex IV assembly gene Surfeit locus protein 1 (*SURF1*) and CRISPR/Cas9 corrected lines. As early as NPCs, deficiencies in the ability to switch to an OXPHOS energetic program and failure to differentiate into neurons were observed in both monolayer and brain organoid cultures. Aberrant cytoarchitecture was also observed in the LS mutant organoids. Importantly, rescue with *SURF1* gene augmentation was able to rescue the observed phenotype, as well as pharmacological induction of PGC1A via bezafibrate, an antilipemic agent that lowers cholesterol and triglycerides (Behar et al., 2000). The second study used three commercially available cell lines derived from patients with LS (Romero-Morales et al., 2020). By whole exome sequencing and mitochondrial sequencing, the putative mutations were identified in the nuclear genes pyruvate dehydrogenase (PDH) in two of the cell lines, and dihydrolipoyl dehydrogenase (DLD); as well as the mitochondrially encoded ATP synthase membrane subunit 6 (MT-ATP6). Although not major differences were observed at the NPCs level, three-dimensional cultures showed significant deficiencies in neuronal commitment and self-organization. The double mutant cell line MT-AP6/PDH showed aberrant organization of the neuroepithelial bud formation as early as day 10 in the brain organoid protocol. These deficiencies were more apparent at day 30 where the loss of the stereotypical luminal and VZs was lost. Moreover, qPCR analysis of the MT-AP6/PDH mutant organoids show and increase expression of the intermediate progenitor cell marker TBR2. Analysis of the mitochondria in the SOX2+ cells in day 30 brain organoids showed aggregated mitochondrial network in the double mutant in comparison with the elongated mitochondria observed in the control. Metabolic deficiencies were also identified at this stage suggesting that the mutant cells rely on aerobic glycolysis or Warburg effect for survival. The inability to switch to a OXPHOS energetic program may also contribute to the effects seeing later in day 100 organoids where the neuronal population in all LS-patient derived organoids were significantly reduced.

The profound dysregulation of corticogenesis in the LS-patient derived organoids may suggest that there is an underestimation of the prevalence of the disease in the population (Feeney et al., 2019). Prenatal genetic evaluation is now being performed for cases where there is a known risk for mitochondrial mutations (White et al., 1999; Craven et al., 2017; Schubert and Vilarinho, 2020). This testing can be performed as early as 10–12 weeks post conception and is usually requested if there is a history of a previously affected child or first-degree relative (Nesbitt et al., 2014). Interpretation of these tests is challenging in the case of mitochondrial mutations due to heteroplasmy; the biopsied tissues may exhibit a different mutational burden compared with other fetal tissues (Harding et al., 1992; Ferlin et al., 1997; Steffann et al., 2007; Nesbitt et al., 2014).

The use of iPSCs and iPSC-derived models can become a platform for research and therapeutic testing as diagnosing and treating this disease remains a challenge due to its heterogeneity and complexity. Work in mice has shown that chronic hypoxia can prevent and even reverse some of the neurological findings in LS (Jain et al., 2019), but these has not been tested in human models yet. The use brain organoids may be an appropriate tool to test whether the reduction of oxygen tension can rescue the LS phenotype and to test for other therapeutic approaches that could alleviate the disease onset and progression. Specifically for the organoids system, there is the capacity to produce individual regions of the brain and investigate the effects of the LS causing mutations in the neuronal subpopulation. Moreover, assembloids (Paşca, 2018; Sloan et al., 2018; Miura et al., 2020) can be generated to investigate the interactions between these regions; specifically the metabolically demanding interneuronal migration that occurs from the ventral forebrain to the dorsal forebrain. Errors in myelination and formation of the axonal bundles can also be an avenue of interest as oligodendrocyte loss has also been observed in LS (Anzil et al., 1981; Pliss et al., 2004; Marton et al., 2019). Also, long term maintenance (>100 days) of brain organoids can be used to understand the role of astrocytes in the pathophysiology of LS.

### Mitochondrial Apoptosis and Neural Cell Specification

Mitochondria are at the crossroads of cell death and metabolism (Rastogi et al., 2019). The BCL-2 family of proteins regulates cell death at the mitochondria (Knudson et al., 1995; Hsu et al., 1997; Kluck et al., 1997, 1999; Vander Heiden et al., 1997; Inohara et al., 1998; Jürgensmeier et al., 1998; Green, 2000; Wei et al., 2000; Ke et al., 2018) and has been implicated in maintaining mitochondrial homeostasis in the absence of a cell death signal (Li et al., 2008, 2013; Rasmussen et al., 2018, 2020; Salisbury-Ruf et al., 2018; Joshi et al., 2020). Programmed cell death (apoptosis) is an integral part of brain development and maturation (Kuan et al., 2000). Immature human brains have nearly 50% more neurons than adults, as many neurons die by apoptosis during normal brain morphogenesis and neuronal histogenesis. Apoptosis is assumed to prevent overgrowth of the neuroepithelium by controlling neural cell numbers in the developing brain (Kuan et al., 2000; Akhtar et al., 2004; Nonomura et al., 2013; Yamaguchi and Miura, 2015). Reduced cell death in mice bearing mutations in pro-apoptotic molecules (e.g., caspase 3, caspase 9) result in severe brain malformations including indented neuroepithelium, compressed brain ventricles, and neural tube closure defects (Nonomura et al., 2013). Additionally, in early brain development, massive cell death is restricted to specific areas, suggesting that local apoptosis might affect the gross organization of the developing organ (Kuan et al., 2000; Akhtar et al., 2004).

Previous studies showed that BAX/BAK deficient cells have some defects in mitochondrial morphology (Karbowski et al., 2006). BAX also is known to colocalize with the primary executioner of mitochondrial fission Dynamin related protein-1 (DRP-1) during early stages of apoptosis (Karbowski et al., 2002). Thus, BAX/BAK are also potential regulators of mitochondrial homeostasis during development. BAX/BAK CRISPR knock-out iPSCs were recently used to study the function of the BCL-2 family in early neurogenesis (Joshi et al., 2020). Day 30 brain organoids generated from the control lines showed the expected markers and architecture for this developmental timepoint. hNPCS stained with PAX6, SOX2 and NESTIN formed the VZ/SVZ around the lumen-like structure. oRGs that were TBR2 positive decorated the edge of the VZ/SVZ, and cortical plate cells positive for TBR1 and CTIP2 were observed Joshi et al. (2020). In contrast, BAX/BAK DKO brain organoids showed a profound loss of cortical organizations and a reduction in the cells positive for the aforementioned markers (Joshi et al., 2020). Analysis of the areas of the organoids that were positive for the NPC marker SOX2, showed aggregation of the mitochondrial network in the DKOs. Control organoids showed a conserved organization of the VZ/SVZ and the expected elongated network for SOX2+ NPCs. This loss of neural markers in conjunction with the disruption of the mitochondrial network in the DKO may suggest that BAX and BAK are fundamental factors maintaining mitochondrial morphology, and their absence generate a downstream effect in the specification of the cell fate. Additional studies are needed to determine the exact mechanisms by which homeostatic regulation of apoptosis controls neurogenesis.

BAX/BAK KO iPSCs allow for the possibility to use this genetic background for the study of deleterious mutations than otherwise would cause cell death. It could also serve as a platform for the study of developmental apoptosis, and the signaling mechanisms that dying cells are involved. Apoptosis is a key regulator for the epithelial-mesenchymal transition (EMT) necessary for the acquisition of cardiac lineage (Fort et al., 2021). EMT is also crucial for the migration of the neural crest cells from the neural tube to the surrounding tissue for the formation of peripheral neurons, melanocytes, craniofacial bones and muscle, among other structures (Hay, 2005).

Particularly in the brain, developmental cell death is crucial for the during development and maturation of the neuronal connections. In the specific case of neuronal pruning, studies suggest that dysregulation of the mitochondrial membrane potential and the production of ROSs are signals for axonal retraction and degradation (Baranov et al., 2019; Lieberman et al., 2019). Yet, it is not known how the mitochondrial dynamics affect these events. Moreover, as apoptotic cells are key for the colonization of the brain by microglia (Arnò et al., 2014; Casano et al., 2016), co-culturing experiments between BAX/BAK KO organoids with microglia can help elucidate the mechanism of microglia migration and proliferation in the developing brain.

### Mitochondria Dysregulation and Hypoxic Injury

Extreme prematurity and complications prior to or during birth can contribute to hypoxic episodes and subsequent brain injury in neonates (Lima et al., 2018). As survival increases, so does the life-long neurological disabilities associated with CNS injury. Mitochondrial energy production is impaired when the tissue oxygen tension is reduced (Santore et al., 2002; Lages et al., 2015). After reoxygenation, mitochondrial respiration transiently resumes but it is later suppressed (Xie and Wolin, 1996; Weidemann and Johnson, 2008; Goda and Kanai, 2012). Mitochondrial hyperpolarization results in the increased production of ROS leading to accumulation of mutations (Grivennikova et al., 2010), decreased metabolic output, and increased susceptibility to cell death (Kudin et al., 2004).

Studies using rat brains showed that the mitochondrial morphology in neurons shifts from elongated to more granular/fragmented appearance after the hypoxic insult (Puka-Sundvall et al., 2000; Hallin et al., 2006). Mitochondrial migration toward the nucleus, as well as accumulation in the perinuclear region, has also been described and associated with the onset of cell death (Northington et al., 2001; Hallin et al., 2006).

Pathological mitochondrial fragmentation has been reported in *in vivo* and *in vitro* models. Loss of the mitochondrial membrane potential leads to the cleavage OPA1 – responsible for inner mitochondrial membrane fusion. Oxygen deprivation increases the amount of a short non-functional form of OPA1, resulting in rapid induction of fission and ultimately cell death (Ehses et al., 2009; Head et al., 2009; Baburamani et al., 2015; Sanderson et al., 2015; Kumar et al., 2016). Interestingly, downregulation of DRP1 can prevent the permeabilization of the mitochondrial membrane and the progression to cell death (Grohm et al., 2012) by potentially preventing cristae remodeling (Ramonet et al., 2013). However, it is not known if these morphological changes, their regulation, and their effects on mitochondrial function are involved during neonatal hypoxia. Prolonged depression in mitochondrial metabolism (Brekke et al., 2017), as well as rapid induction of mitochondrial fragmentation (Demarest et al., 2016), validate the emerging evidence of the mitochondria as central regulators of the hypoxic injury cascade.

Results from animal models have shown that gray and white matter are the most vulnerable areas of the brain to neonatal hypoxia ischemic injury. The cerebral cortex is predominantly affected (Volpe, 2012) and the localization and extent of the insult have been shown to correlate with specific neurodevelopmental symptoms later in life (Gonzalez and Miller, 2006; Steinman et al., 2009). Considering that the spatiotemporal regulation of neuronal proliferation, migration and differentiation varies among mammals, a human derived model is needed to understand species specific variation.

The cerebral cortex is highly sensitive to hypoxic insult. Cell death in this region presents a laminar distribution, especially involving layers III and V (Volpe, 2012). Work in early stages of brain organoid formation have shown that hypoxic stress causes growth arrest, massive cell death (Daviaud et al., 2019) and reduced expression of the cortical markers TBR1, CTIP2, SATB2, and the astrocyte marker GFAP (Boisvert et al., 2019). In a model of encephalopathy of prematurity, human cortical spheroids resembling the cerebral cortex at midgestation were exposed to 48h of low oxygen tension (<1%) (Paşca et al., 2019). Hypoxia induced a reduction in TBR2+ intermediate progenitor cells and an increase in CTIP2+ cells, suggesting premature neural differentiation (Paşca et al., 2019).

As the mitochondrial network undergoes remodeling in the different cellular niches of the developing brain (Khacho et al., 2016, 2019; Khacho and Slack, 2018), it would be interesting to analyze if the that hypoxic insult affects each neural population differently due to dysregulation of mitochondrial dynamics. Hypoxic insult may affect the mitochondrial network plasticity due to dysregulation of the mitochondrial dynamic’s machinery. Rates of fission may increase as OPA-1 undergoes cleavage into short non-functional form (Ehses et al., 2009). The high sensitivity to hypoxia of intermediate progenitor cells may be due to changes in mitochondrial dynamics and morphology. As the intermediate progenitor cells have been proposed to undergo cell death during hypoxic injury, characterization in this context has been challenging. Brain organoid models could also be used to examine other determinants of mitochondrial homeostasis including mitochondrial biogenesis and mitophagy as well as to analyze the link between oxygen deprivation and mitochondrial disfunction. Mitochondrial disorders can intensify the effects of hypoxia by exacerbating potential deleterious reactions (Volpe, 2012) or diminishing prosurvival mechanisms (Ten and Starkov, 2012). Cell lines generated from mitochondrial diseases could be used to determine how mitochondrial impairment affects the response to hypoxia, as failure of the metabolic machinery has been shown to contribute to brain injury in mouse models (Niatsetskaya et al., 2012). Paradoxically, hypoxia has been shown to reverse some of the effects of mitochondrial disorders such as Leigh syndrome (Jain et al., 2019).

## Conclusion

The underlying mechanisms responsible for the extraordinarily complex cognitive capacity of the human brain are beginning to be elucidated. A central principle governing brain development is the precise spatiotemporally coordinated birth of, and interactions between, a vast number and types of neurons. Recent studies point to a new mechanism – one hinted at previously, but currently poorly understood. A spectrum of mitochondrial fitness properties could provide internal support to the intrinsic developmental programs of various neuronal types, while also being responsive to environmental and intercellular signals. In humans, defects in mitochondrial homeostasis are linked to conditions such as Leigh syndrome (a neurometabolic disorder), MELAS syndrome (mitochondrial encephalopathy, lactic acidosis, and stroke-like episodes) (a neurodegenerative disorder), and Autism Spectrum Disorder (a neurodevelopmental disease). But, the mechanisms by which mitochondrial morphology and function influence human brain development are largely unexplored.

A massive challenge in the past has been the lack of appropriate model systems that can reproducibly recapitulate the heterogeneous nature as well as the highly complex properties of the human brain. As reviewed here, brain organoids reproduce several key features of human cortical development, including progenitor zone organization, neurogenesis, migration, and synaptic activity. Studies using human brain organoids will continue to provide insight into the mitochondrial mechanisms underlying neurogenesis as well as provide new angles to understand mitochondrial disorders.

## Author Contributions

AR-M performed the initial literature review. AR-M and VG wrote the manuscript. Both authors contributed to the article and approved the submitted version.

## Conflict of Interest

The authors declare that the research was conducted in the absence of any commercial or financial relationships that could be construed as a potential conflict of interest.

## Publisher’s Note

All claims expressed in this article are solely those of the authors and do not necessarily represent those of their affiliated organizations, or those of the publisher, the editors and the reviewers. Any product that may be evaluated in this article, or claim that may be made by its manufacturer, is not guaranteed or endorsed by the publisher.

## References

[B1] Aaku-Saraste E. Hellwig A. Huttner W. B. (1996). Loss of occludin and functional tight junctions, but not ZO-1, during neural tube closure – remodeling of the neuroepithelium prior to neurogenesis. *Dev. Biol.* 180 664–679. 10.1006/dbio.1996.0336 8954735

[B2] Aaku-Saraste E. Oback B. Hellwig A. Huttner W. B. (1997). Neuroepithelial cells downregulate their plasma membrane polarity prior to neural tube closure and neurogenesis. *Mech. Dev.* 69 71–81. 10.1016/S0925-4773(97)00156-1 9486532

[B3] Acín-Pérez R. Fernández-Silva P. Peleato M. L. Pérez-Martos A. Enriquez J. A. (2008). Respiratory active mitochondrial supercomplexes. *Mol. Cell* 32 529–539. 10.1016/j.molcel.2008.10.021 19026783

[B4] Agana M. Frueh J. Kamboj M. Patel D. R. Kanungo S. (2018). Common metabolic disorder (inborn errors of metabolism) concerns in primary care practice. *Ann. Transl. Med.* 6 469–469. 10.21037/atm.2018.12.34 30740400 PMC6331353

[B5] Agius E. Soukkarieh C. Danesin C. Kan P. Takebayashi H. Soula C. (2004). Converse control of oligodendrocyte and astrocyte lineage development by Sonic hedgehog in the chick spinal cord. *Dev. Biol.* 270 308–321. 10.1016/j.ydbio.2004.02.015 15183716

[B6] Agostini M. Romeo F. Inoue S. Niklison-Chirou M. V. Elia A. J. Dinsdale D. (2016). Metabolic reprogramming during neuronal differentiation. *Cell Death Differ.* 23 1502–1514. 10.1038/cdd.2016.36 27058317 PMC5072427

[B7] Akhtar R. S. Ness J. M. Roth K. A. (2004). Bcl-2 family regulation of neuronal development and neurodegeneration. *Biochim. Biophys. Acta Mol. Cell Res.* 1644 189–203. 10.1016/j.bbamcr.2003.10.013 14996503

[B8] Alavi M. V. Bette S. Schimpf S. Schuettauf F. Schraermeyer U. Wehrl H. F. (2007). A splice site mutation in the murine Opa1 gene features pathology of autosomal dominant optic atrophy. *Brain* 130 1029–1042. 10.1093/brain/awm005 17314202

[B9] Alcamo E. A. Chirivella L. Dautzenberg M. Dobreva G. Fariñas I. Grosschedl R. (2008). Satb2 regulates callosal projection neuron identity in the developing cerebral cortex. *Neuron* 57 364–377. 10.1016/j.neuron.2007.12.012 18255030

[B10] Alexander C. Votruba M. Pesch U. E. A. Thiselton D. L. Mayer S. Moore A. (2000). OPA1, encoding a dynamin-related GTPase, is mutated in autosomal dominant optic atrophy linked to chromosome 3q28. *Nat. Genet.* 26 211–215. 10.1038/79944 11017080

[B11] Altmann C. R. Brivanlou A. H. (2001). “Neural patterning in the vertebrate embryo,” in *International Review of Cytology*, ed. Jeon K. W. (Cambridge, MA: Academic Press), 447–482. 10.1016/S0074-7696(01)03013-3 11131523

[B12] Alvarez-Buylla A. García-Verdugo J. M. Tramontin A. D. (2001). A unified hypothesis on the lineage of neural stem cells. *Nat. Rev. Neurosci.* 2 287–293. 10.1038/35067582 11283751

[B13] Amaral A. I. Hadera M. G. Tavares J. M. Kotter M. R. N. Sonnewald U. (2016). Characterization of glucose-related metabolic pathways in differentiated rat oligodendrocyte lineage cells. *Glia* 64 21–34. 10.1002/glia.22900 26352325 PMC4832329

[B14] Ambasudhan R. Talantova M. Coleman R. Yuan X. Zhu S. Lipton S. A. (2011). Direct reprogramming of adult human fibroblasts to functional neurons under defined conditions. *Cell Stem Cell* 9 113–118. 10.1016/j.stem.2011.07.002 21802386 PMC4567246

[B15] Anesti V. Scorrano L. (2006). The relationship between mitochondrial shape and function and the cytoskeleton. *Biochim. Biophys. Acta Bioenerg.* 1757 692–699. 10.1016/j.bbabio.2006.04.013 16729962

[B16] Anthony T. E. Klein C. Fishell G. Heintz N. (2004). Radial glia serve as neuronal progenitors in all regions of the central nervous system. *Neuron* 41 881–890. 10.1016/S0896-6273(04)00140-0 15046721

[B17] Anzil A. P. Weindl A. Struppler A. (1981). Ultrastructure of a cerebral white matter lesion in a 41-year-old man with Leigh’s encephalomyelopathy (LEM). *Acta Neuropathol. Suppl.* 7 233–238. 10.1007/978-3-642-81553-9_69 6939243

[B18] Arbour N. Vanderluit J. L. Le Grand J. N. Jahani-Asl A. Ruzhynsky V. A. Cheung E. C. C. (2008). Mcl-1 is a key regulator of apoptosis during CNS development and after DNA damage. *J. Neurosci.* 28 6068–6078. 10.1523/JNEUROSCI.4940-07.2008 18550749 PMC2681190

[B19] Arlotta P. Paşca S. P. (2019). Cell diversity in the human cerebral cortex: from the embryo to brain organoids. *Curr. Opin. Neurobiol.* 56 194–198. 10.1016/j.conb.2019.03.001 31051421

[B20] Arlotta P. Molyneaux B. J. Chen J. Inoue J. Kominami R. MacKlis J. D. (2005). Neuronal subtype-specific genes that control corticospinal motor neuron development in vivo. *Neuron* 45 207–221. 10.1016/j.neuron.2004.12.036 15664173

[B21] Arnò B. Grassivaro F. Rossi C. Bergamaschi A. Castiglioni V. Furlan R. (2014). Neural progenitor cells orchestrate microglia migration and positioning into the developing cortex. *Nat. Commun.* 5 1–13. 10.1038/ncomms6611 25425146

[B22] Arnold S. J. Huang G.-J. Cheung A. F. P. Era T. Nishikawa S.-I. Bikoff E. K. (2008). The T-box transcription factor Eomes/Tbr2 regulates neurogenesis in the cortical subventricular zone. *Genes Dev.* 22 2479–2484. 10.1101/gad.475408 18794345 PMC2546697

[B23] Audano M. Pedretti S. Crestani M. Caruso D. De Fabiani E. Mitro N. (2019). Mitochondrial dysfunction increases fatty acid β−oxidation and translates into impaired neuroblast maturation. *FEBS Lett.* 593 3173–3189. 10.1002/1873-3468.13584 31432511

[B24] Azizi A. Herrmann A. Wan Y. Buse S. J. R. P. Keller P. J. Goldstein R. E. (2020). Nuclear crowding and nonlinear diffusion during interkinetic nuclear migration in the zebrafish retina. *Elife* 9:e58635. 10.7554/eLife.58635 33021471 PMC7538155

[B25] Baala L. Briault S. Etchevers H. C. Laumonnier F. Natiq A. Amiel J. (2007). Homozygous silencing of T-box transcription factor EOMES leads to microcephaly with polymicrogyria and corpus callosum agenesis. *Nat. Genet.* 39 454–456. 10.1038/ng1993 17353897

[B26] Baburamani A. A. Hurling C. Stolp H. Sobotka K. Gressens P. Hagberg H. (2015). Mitochondrial Optic Atrophy (OPA) 1 processing is altered in response to neonatal hypoxic-ischemic brain injury. *Int. J. Mol. Sci.* 16 22509–22526. 10.3390/ijms160922509 26393574 PMC4613321

[B27] Bagley J. A. Reumann D. Bian S. Lévi-Strauss J. Knoblich J. A. (2017). Fused cerebral organoids model interactions between brain regions. *Nat. Methods* 14 743–751. 10.1038/nmeth.4304 28504681 PMC5540177

[B28] Bain G. Kitchens D. Yao M. Huettner J. E. Gottlieb D. I. (1995). Embryonic stem cells express neuronal properties in vitro. *Dev. Biol.* 168 342–357. 10.1006/dbio.1995.1085 7729574

[B29] Bang A. G. Papalopulu N. Kintner C. Goulding M. D. (1997). Expression of Pax-3 is initiated in the early neural plate by posteriorizing signals produced by the organizer and by posterior non-axial mesoderm. *Development* 124 2075–2085. 10.1242/dev.124.10.2075 9169853

[B30] Baranov S. V. Baranova O. V. Yablonska S. Suofu Y. Vazquez A. L. Kozai T. D. Y. (2019). Mitochondria modulate programmed neuritic retraction. *Proc. Natl. Acad. Sci. U.S.A.* 116 650–659. 10.1073/pnas.1811021116 30584104 PMC6329959

[B31] Barnhart E. L. (2016). Mechanics of mitochondrial motility in neurons. *Curr. Opin. Cell Biol.* 38 90–99. 10.1016/j.ceb.2016.02.022 26986984

[B32] Barth K. A. Kishimoto Y. Rohr K. B. Seydler C. Schulte-Merker S. Wilson S. W. (1999). Bmp activity establishes a gradient of positional information throughout the entire neural plate. *Development* 126 4977–4987. 10.1242/dev.126.22.4977 10529416

[B33] Bartsakoulia M. Pyle A. Troncoso-Chandía D. Vial-Brizzi J. Paz-Fiblas M. V. Duff J. (2018). A novel mechanism causing imbalance of mitochondrial fusion and fission in human myopathies. *Hum. Mol. Genet.* 27 1186–1195. 10.1093/hmg/ddy033 29361167 PMC6159537

[B34] Battefeld A. Popovic M. A. de Vries S. I. Kole M. H. P. (2019). High-frequency microdomain Ca 2+ transients and waves during early myelin internode remodeling. *Cell Rep.* 26 182–191.e5. 10.1016/j.celrep.2018.12.039 30605675 PMC6316190

[B35] Battiste J. Helms A. W. Kim E. J. Savage T. K. Lagace D. C. Mandyam C. D. (2007). Ascl 1 defines sequentially generated lineage-resricted neuronal and oligodendrocyte precursor cells in the spinal cord. *Development* 134 285–293. 10.1242/dev.02727 17166924

[B36] Baum T. Gama V. (2021). Dynamic properties of mitochondria during human corticogenesis. *Development* 148:dev194183. 10.1242/dev.194183 33608250 PMC7903999

[B37] Bayer S. A. Altman J. (2007). *The Human Brain During the Early First Trimester.* Boca Raton, FL: CRC Press, 10.1201/9781420003284

[B38] Beckervordersandforth R. Ebert B. Schäffner I. Moss J. Fiebig C. Shin J. (2017). Role of mitochondrial metabolism in the control of early lineage progression and aging phenotypes in adult hippocampal neurogenesis. *Neuron* 93 560–573.e6. 10.1016/j.neuron.2016.12.017 28111078 PMC5300896

[B39] Bedogni F. Hodge R. D. Elsen G. E. Nelson B. R. Daza R. A. M. Beyer R. P. (2010). Tbr1 regulates regional and laminar identity of postmitotic neurons in developing neocortex. *Proc. Natl. Acad. Sci. U.S.A.* 107 13129–13134. 10.1073/pnas.1002285107 20615956 PMC2919950

[B40] Behar S. Brunner D. Kaplinsky E. Mandelzweig L. Benderly M. (2000). Secondary prevention by raising HDL cholesterol and reducing triglycerides in patients with coronary artery disease: the bezafibrate infarction prevention (BIP) study. *Circulation* 102 21–27. 10.1161/01.CIR.102.1.21 10880410

[B41] Bélanger M. Allaman I. Magistretti P. J. (2011). Brain energy metabolism: focus on astrocyte-neuron metabolic cooperation. *Cell Metab.* 14 724–738. 10.1016/j.cmet.2011.08.016 22152301

[B42] Ben-Reuven L. Reiner O. (2020). Toward spatial identities in human brain organoids-on-chip induced by morphogen-soaked beads. *Bioengineering* 7 1–17. 10.3390/bioengineering7040164 33352983 PMC7766968

[B43] Bentivoglio M. Mazzarello P. (1999). The history of radial glia. *Brain Res. Bull.* 49 305–315. 10.1016/S0361-9230(99)00065-9 10452351

[B44] Berg S. Kutra D. Kroeger T. Straehle C. N. Kausler B. X. Haubold C. (2019). ilastik: interactive machine learning for (bio)image analysis. *Nat. Methods* 16 1226–1232. 10.1038/s41592-019-0582-9 31570887

[B45] Bertrand N. Medevielle F. Pituello F. (2000). FGF signalling controls the timing of Pax6 activation in the neural tube. *Development* 127 4837–4843. 10.1242/dev.127.22.4837 11044398

[B46] Birey F. Andersen J. Makinson C. D. Islam S. Wei W. Huber N. (2017). Assembly of functionally integrated human forebrain spheroids. *Nature* 545 54–59. 10.1038/nature22330 28445465 PMC5805137

[B47] Boisvert E. M. Means R. E. Michaud M. Madri J. A. Katz S. G. (2019). Minocycline mitigates the effect of neonatal hypoxic insult on human brain organoids. *Cell Death Dis.* 10:325. 10.1038/s41419-019-1553-x 30975982 PMC6459920

[B48] Bonnefont J. Tiberi L. van den Ameele J. Potier D. Gaber Z. B. Lin X. (2019). Cortical neurogenesis requires bcl6-mediated transcriptional repression of multiple self-renewal-promoting extrinsic pathways. *Neuron* 103 1096–1108.e4. 10.1016/j.neuron.2019.06.027 31353074 PMC6859502

[B49] Bowers M. Liang T. Gonzalez-Bohorquez D. Zocher S. Jaeger B. N. Kovacs W. J. (2020). FASN-dependent lipid metabolism links neurogenic stem/progenitor cell activity to learning and memory deficits. *Cell Stem Cell* 27 98–109.e11. 10.1016/j.stem.2020.04.002 32386572

[B50] Bredenoord A. L. Clevers H. Knoblich J. A. (2017). Human tissues in a dish: the research and ethical implications of organoid technology. *Science* 355:eaaf9414. 10.1126/science.aaf9414 28104841

[B51] Brekke E. Berger H. R. Widerøe M. Sonnewald U. Morken T. S. (2017). Glucose and intermediary metabolism and astrocyte–neuron interactions following neonatal hypoxia–ischemia in rat. *Neurochem. Res.* 42 115–132. 10.1007/s11064-016-2149-9 28019006

[B52] Brennand K. Savas J. N. Kim Y. Tran N. Simone A. Hashimoto-Torii K. (2015). Phenotypic differences in hiPSC NPCs derived from patients with schizophrenia. *Mol. Psychiatry* 20 361–368. 10.1038/mp.2014.22 24686136 PMC4182344

[B53] Britanova O. de Juan Romero C. Cheung A. Kwan K. Y. Schwark M. Gyorgy A. (2008). Satb2 is a postmitotic determinant for upper-layer neuron specification in the neocortex. *Neuron* 57 378–392. 10.1016/j.neuron.2007.12.028 18255031

[B54] Brüstle O. Spiro A. C. Karram K. Choudhary K. Okabe S. Mckay R. D. G. (1997). In vitro-generated neural precursors participate in mammalian brain development. *Proc. Natl. Acad. Sci. U.S.A.* 94 14809–14814. 10.1073/pnas.94.26.14809 9405695 PMC25119

[B55] Bultje R. S. Castaneda-Castellanos D. R. Jan L. Y. Jan Y. N. Kriegstein A. R. Shi S. H. (2009). Mammalian Par3 regulates progenitor cell asymmetric division via notch signaling in the developing neocortex. *Neuron* 63 189–202. 10.1016/j.neuron.2009.07.004 19640478 PMC2736606

[B56] Bystron I. Blakemore C. Rakic P. (2008). Development of the human cerebral cortex: boulder committee revisited. *Nat. Rev. Neurosci.* 9 110–122. 10.1038/nrn2252 18209730

[B57] Cabello-Rivera D. Sarmiento-Soto H. López-Barneo J. Muñoz-Cabello A. M. (2019). Mitochondrial complex i function is essential for neural stem/progenitor cells proliferation and differentiation. *Front. Neurosci.* 13:664. 10.3389/fnins.2019.00664 31297047 PMC6607990

[B58] Caiazzo M. Dell’Anno M. T. Dvoretskova E. Lazarevic D. Taverna S. Leo D. (2011). Direct generation of functional dopaminergic neurons from mouse and human fibroblasts. *Nature* 476 224–227. 10.1038/nature10284 21725324

[B59] Calegari F. Haubensak W. Haffher C. Huttner W. B. (2005). Selective lengthening of the cell cycle in the neurogenic subpopulation of neural progenitor cells during mouse brain development. *J. Neurosci.* 25 6533–6538. 10.1523/JNEUROSCI.0778-05.2005 16014714 PMC6725437

[B60] Callaerts P. Halder G. Gehring W. J. (1997). PAX-6 in development and evolution. *Annu. Rev. Neurosci.* 20 483–532. 10.1146/annurev.neuro.20.1.483 9056723

[B61] Cameron R. S. Rakic P. (1991). Glial cell lineage in the cerebral cortex: a review and synthesis. *Glia* 4 124–137. 10.1002/glia.440040204 1827774

[B62] Camp J. G. Badsha F. Florio M. Kanton S. Gerber T. Wilsch-Bräuninger M. (2015). Human cerebral organoids recapitulate gene expression programs of fetal neocortex development. *Proc. Natl. Acad. Sci. U.S.A.* 112:201520760. 10.1073/pnas.1520760112 26644564 PMC4697386

[B63] Campbell K. Götz M. (2002). Radial glia: multi-purpose cells for vertebrate brain development. *Trends Neurosci.* 25 235–238. 10.1016/S0166-2236(02)02156-2 11972958

[B64] Carelli V. Musumeci O. Caporali L. Zanna C. La Morgia C. Del Dotto V. (2015). Syndromic parkinsonism and dementia associated with OPA1 missense mutations. *Ann. Neurol.* 78 21–38. 10.1002/ana.24410 25820230 PMC5008165

[B65] Cartier L. Laforge T. Feki A. Arnaudeau S. Dubois-Dauphin M. Krause K. H. (2006). Pax6-induced alteration of cell fate: shape changes, expression of neuronal α tubulin, postmitotic phenotype, and cell migration. *J. Neurobiol.* 66 421–436. 10.1002/neu.20225 16425216

[B66] Casano A. M. Albert M. Peri F. (2016). Developmental apoptosis mediates entry and positioning of microglia in the zebrafish brain. *Cell Rep.* 16 897–906. 10.1016/j.celrep.2016.06.033 27425604

[B67] Cederquist G. Y. Asciolla J. J. Tchieu J. Walsh R. M. Cornacchia D. Resh M. D. (2019). Specification of positional identity in forebrain organoids. *Nat. Biotechnol.* 37 436–444. 10.1038/s41587-019-0085-3 30936566 PMC6447454

[B68] Cerutti R. Pirinen E. Lamperti C. Marchet S. Sauve A. A. Li W. (2014). NAD+-dependent activation of Sirt1 corrects the phenotype in a mouse model of mitochondrial disease. *Cell Metab.* 19 1042–1049. 10.1016/j.cmet.2014.04.001 24814483 PMC4051987

[B69] Chakrabarty R. P. Chandel N. S. (2021). Mitochondria as signaling organelles control mammalian stem cell fate. *Cell Stem Cell* 28 394–408. 10.1016/j.stem.2021.02.011 33667360 PMC7944920

[B70] Chambers S. M. Fasano C. A. Papapetrou E. P. Tomishima M. Sadelain M. Studer L. (2009). Highly efficient neural conversion of human ES and iPS cells by dual inhibition of SMAD signaling. *Nat. Biotechnol.* 27 275–280. 10.1038/nbt.1529 19252484 PMC2756723

[B71] Chan D. C. (2012). Fusion and fission: interlinked processes critical for mitochondrial health. *Annu. Rev. Genet.* 46 265–287. 10.1146/annurev-genet-110410-132529 22934639

[B72] Chanas-Sacre G. Rogister B. Moonen G. Leprince P. (2000). Radial glia phenotype: origin, regulation, and transdifferentiation. *J. Neurosci. Res.* 61 357–363. 10.1002/1097-4547(20000815)61:4<357::AID-JNR1>3.0.CO;2-7 10931521

[B73] Chandrasekaran A. Avci H. X. Ochalek A. Rösingh L. N. Molnár K. László L. (2017). Comparison of 2D and 3D neural induction methods for the generation of neural progenitor cells from human induced pluripotent stem cells. *Stem Cell Res.* 25 139–151. 10.1016/j.scr.2017.10.010 29128818

[B74] Chang C. R. Blackstone C. (2010). “Dynamic regulation of mitochondrial fission through modification of the dynamin-related protein Drp1,” in *Annals of the New York Academy of Sciences*, ed. Braaten D. (Hoboken, NJ: John Wiley & Sons Ltd), 34–39. 10.1111/j.1749-6632.2010.05629.x PMC5585781 20649536

[B75] Chatzi C. Brade T. Duester G. (2011). Retinoic acid functions as a Key GABAergic differentiation signal in the basal ganglia. *PLoS Biol.* 9:e1000609. 10.1371/journal.pbio.1000609 21532733 PMC3075211

[B76] Chau K. F. Shannon M. L. Fame R. M. Fonseca E. Mullan H. Johnson M. B. (2018). Downregulation of ribosome biogenesis during early forebrain development. *Elife* 7:e36998. 10.7554/eLife.36998 29745900 PMC5984036

[B77] Chen B. Schaevitz L. R. McConnell S. K. (2005). Fezl regulates the differentiation and axon targeting of layer 5 subcortical projection neurons in cerebral cortex. *Proc. Natl. Acad. Sci. U.S.A.* 102 17184–17189. 10.1073/pnas.0508732102 16284245 PMC1282569

[B78] Chen B. Wang S. S. Hattox A. M. Rayburn H. Nelson S. B. McConnell S. K. (2008). The Fezf2-Ctip2 genetic pathway regulates the fate choice of subcortical projection neurons in the developing cerebral cortex. *Proc. Natl. Acad. Sci. U.S.A.* 105 11382–11387. 10.1073/pnas.0804918105 18678899 PMC2495013

[B79] Chen H. Chan D. C. (2004). Mitochondrial dynamics in mammals. *Curr. Top. Dev. Biol.* 59 119–144. 10.1016/S0070-2153(04)59005-1 14975249

[B80] Chen H. Chan D. C. (2017). Mitochondrial dynamics in regulating the unique phenotypes of cancer and stem cells. *Cell Metab.* 26 39–48. 10.1016/j.cmet.2017.05.016 28648983 PMC5539982

[B81] Chen H. Detmer S. A. Ewald A. J. Griffin E. E. Fraser S. E. Chan D. C. (2003). Mitofusins Mfn1 and Mfn2 coordinately regulate mitochondrial fusion and are essential for embryonic development. *J. Cell Biol.* 160 189–200. 10.1083/jcb.200211046 12527753 PMC2172648

[B82] Chenn A. McConnell S. K. (1995). Cleavage orientation and the asymmetric inheritance of notchl immunoreactivity in mammalian neurogenesis. *Cell* 82 631–641. 10.1016/0092-8674(95)90035-7 7664342

[B83] Chenn A. Zhang Y. A. Chang B. T. McConnell S. K. (1998). Intrinsic polarity of mammalian neuroepithelial cells. *Mol. Cell. Neurosci.* 11 183–193. 10.1006/mcne.1998.0680 9675050

[B84] Choi B. H. (1981). Radial glia of developing human fetal spinal cord: Golgi, immunohistochemical and electron microscopic study. *Dev. Brain Res.* 1 249–267. 10.1016/0165-3806(81)90112-7 7013909

[B85] Chou C. H. Lin C. C. Yang M. C. Wei C. C. de Liao H. Lin R. C. (2012). GSK3beta-Mediated Drp1 phosphorylation induced elongated mitochondrial morphology against oxidative stress. *PLoS One* 7:e49112. 10.1371/journal.pone.0049112 23185298 PMC3502545

[B86] Chung S. Arrell D. K. Faustino R. S. Terzic A. Dzeja P. P. (2010). Glycolytic network restructuring integral to the energetics of embryonic stem cell cardiac differentiation. *J. Mol. Cell. Cardiol.* 48 725–734. 10.1016/j.yjmcc.2009.12.014 20045004 PMC2837789

[B87] Clancy B. Darlington R. B. Finlay B. L. (2001). Translating developmental time across mammalian species. *Neuroscience* 105 7–17. 10.1016/S0306-4522(01)00171-3 11483296

[B88] Cogliati S. Enriquez J. A. Scorrano L. (2016). Mitochondrial cristae: where beauty meets functionality. *Trends Biochem. Sci.* 41 261–273. 10.1016/j.tibs.2016.01.001 26857402

[B89] Cogliati S. Frezza C. Soriano M. E. Varanita T. Quintana-Cabrera R. Corrado M. (2013). Mitochondrial cristae shape determines respiratory chain supercomplexes assembly and respiratory efficiency. *Cell* 155 160–171. 10.1016/j.cell.2013.08.032 24055366 PMC3790458

[B90] Colas J. F. Schoenwolf G. C. (2001). Towards a cellular and molecular understanding of neurulation. *Dev. Dyn.* 221 117–145. 10.1002/dvdy.1144 11376482

[B91] Cook G. M. W. Lewis K. E. Keynes R. J. (2017). “Neural patterning: spinal cord segmentation and somite patterning✩,” in *Reference Module in Neuroscience and Biobehavioral Psychology*, ed. Stein J. (Amsterdam: Elsevier), 537–544. 10.1016/B978-0-12-809324-5.02586-4

[B92] Craven L. Alston C. L. Taylor R. W. Turnbull D. M. (2017). Recent advances in mitochondrial disease. *Annu. Rev. Genomics Hum. Genet.* 18 257–275. 10.1146/annurev-genom-091416-035426 28415858

[B93] D’Arcangelo G. Miao G. G. Chen S. C. Scares H. D. Morgan J. I. Curran T. (1995). A protein related to extracellular matrix proteins deleted in the mouse mutant reeler. *Nature* 374 719–723. 10.1038/374719a0 7715726

[B94] Das A. M. Steuerwald U. Illsinger S. (2010). Inborn errors of energy metabolism associated with myopathies. *J. Biomed. Biotechnol.* 2010:340849. 10.1155/2010/340849 20589068 PMC2877206

[B95] Daviaud N. Chevalier C. Friedel R. H. Zou H. (2019). Distinct vulnerability and resilience of human neuroprogenitor subtypes in cerebral organoid model of prenatal hypoxic injury. *Front. Cell. Neurosci.* 13:336. 10.3389/fncel.2019.00336 31417360 PMC6682705

[B96] Davies K. M. Strauss M. Daum B. Kief J. H. Osiewacz H. D. Rycovska A. (2011). Macromolecular organization of ATP synthase and complex I in whole mitochondria. *Proc. Natl. Acad. Sci. U.S.A.* 108 14121–14126. 10.1073/pnas.1103621108 21836051 PMC3161574

[B97] DeBerardinis R. J. Chandel N. S. (2020). We need to talk about the Warburg effect. *Nat. Metab.* 2 127–129. 10.1038/s42255-020-0172-2 32694689

[B98] Dehay C. Kennedy H. (2020). Evolution of the human brain. *Science* 369 506–507. 10.1126/science.abd1840 32732410

[B99] Del Bene F. Wehman A. M. Link B. A. Baier H. (2008). Regulation of neurogenesis by interkinetic nuclear migration through an apical-basal notch gradient. *Cell* 134 1055–1065. 10.1016/j.cell.2008.07.017 18805097 PMC2628487

[B100] Del Dotto V. Mishra P. Vidoni S. Fogazza M. Maresca A. Caporali L. (2017). OPA1 isoforms in the hierarchical organization of mitochondrial functions. *Cell Rep.* 19 2557–2571. 10.1016/j.celrep.2017.05.073 28636943

[B101] Delettre C. Lenaers G. Griffoin J. M. Gigarel N. Lorenzo C. Belenguer P. (2000). Nuclear gene OPA1, encoding a mitochondrial dynamin-related protein, is mutated in dominant optic atrophy. *Nat. Genet.* 26 207–210. 10.1038/79936 11017079

[B102] Delgado R. N. Allen D. E. Keefe M. G. Mancia Leon W. R. Ziffra R. S. Crouch E. E. (2022). Individual human cortical progenitors can produce excitatory and inhibitory neurons. *Nature* 601 397–403. 10.1038/s41586-021-04230-7 34912114 PMC8994470

[B103] Demarest T. G. Waite E. L. Kristian T. Puche A. C. Waddell J. McKenna M. C. (2016). Sex-dependent mitophagy and neuronal death following rat neonatal hypoxia–ischemia. *Neuroscience* 335 103–113. 10.1016/j.neuroscience.2016.08.026 27555552 PMC5580242

[B104] Deneen B. Ho R. Lukaszewicz A. Hochstim C. J. Gronostajski R. M. Anderson D. J. (2006). The transcription factor nfia controls the onset of gliogenesis in the developing spinal cord. *Neuron* 52 953–968. 10.1016/j.neuron.2006.11.019 17178400

[B105] Desai A. R. McConnell S. K. (2000). Progressive restriction in fate potential by neural progenitors during cerebral cortical development. *Development* 127 2863–2872. 10.1242/dev.127.13.2863 10851131

[B106] Dhara S. K. Stice S. L. (2008). Neural differentiation of human embryonic stem cells. *J. Cell. Biochem.* 105 633–640. 10.1002/jcb.21891 18759328 PMC2574851

[B107] Di Gregorio A. (2020). The notochord gene regulatory network in chordate evolution: conservation and divergence from ciona to vertebrates. *Curr. Topics Dev. Biol.* 139 325–374. 10.1016/bs.ctdb.2020.01.002 32450965 PMC13114679

[B108] Di Meo I. Marchet S. Lamperti C. Zeviani M. Viscomi C. (2017). AAV9-based gene therapy partially ameliorates the clinical phenotype of a mouse model of Leigh syndrome. *Gene Ther.* 24 661–667. 10.1038/gt.2017.53 28753212 PMC5658670

[B109] Di Pietro N. C. Whiteley L. Illes J. (2012). Treatments and services for neurodevelopmental disorders on advocacy websites: information or evaluation? *Neuroethics* 5 197–209. 10.1007/s12152-011-9102-z

[B110] Diez del Corral R. Storey K. G. (2004). Opposing FGF and retinoid pathways: a signalling switch that controls differentiation and patterning onset in the extending vertebrate body axis. *Bioessays* 26 857–869. 10.1002/bies.20080 15273988

[B111] Diez del Corral R. Breitkreuz D. N. Storey K. G. (2002). Onset of neuronal differentiation is regulated by paraxial mesoderm and requires attenuation of FGF signalling. *Development* 129 1681–1691. 10.1242/dev.129.7.1681 11923204

[B112] Dimou L. Simon C. Kirchhoff F. Takebayashi H. Götz M. (2008). Progeny of Olig2-expressing progenitors in the gray and white matter of the adult mouse cerebral cortex. *J. Neurosci.* 28 10434–10442. 10.1523/JNEUROSCI.2831-08.2008 18842903 PMC6671038

[B113] Dolmetsch R. Geschwind D. H. (2011). The human brain in a dish: the promise of iPSC-derived neurons. *Cell* 145 831–834. 10.1016/j.cell.2011.05.034 21663789 PMC3691069

[B114] Dominguez M. H. Ayoub A. E. Rakic P. (2013). POU-III transcription factors (Brn1, Brn2, and Oct6) influence neurogenesis, molecular identity, and migratory destination of upper-layer cells of the cerebral cortex. *Cereb. Cortex* 23 2632–2643. 10.1093/cercor/bhs252 22892427 PMC3792741

[B115] Douvaras P. Fossati V. (2015). Generation and isolation of oligodendrocyte progenitor cells from human pluripotent stem cells. *Nat. Protoc.* 10 1143–1154. 10.1038/nprot.2015.075 26134954

[B116] Douvaras P. Wang J. Zimmer M. Hanchuk S. O’Bara M. A. Sadiq S. (2014). Efficient generation of myelinating oligodendrocytes from primary progressive multiple sclerosis patients by induced pluripotent stem cells. *Stem Cell Reports* 3 250–259. 10.1016/j.stemcr.2014.06.012 25254339 PMC4176529

[B117] Dudkina N. V. Eubel H. Keegstra W. Boekema E. J. Braun H. P. (2005). Structure of a mitochondrial supercomplex formed by respiratory-chain complexes I and III. *Proc. Natl. Acad. Sci. U.S.A.* 102 3225–3229. 10.1073/pnas.0408870102 15713802 PMC552927

[B118] Dulabon L. Olson E. C. Taglienti M. G. Eisenhuth S. McGrath B. Walsh C. A. (2000). Reelin binds α3β1 integrin and inhibits neuronal migration. *Neuron* 27 33–44. 10.1016/S0896-6273(00)00007-6 10939329

[B119] Echelard Y. Epstein D. J. St-Jacques B. Shen L. Mohler J. McMahon J. A. (1993). Sonic hedgehog, a member of a family of putative signaling molecules, is implicated in the regulation of CNS polarity. *Cell* 75 1417–1430. 10.1016/0092-8674(93)90627-3 7916661

[B120] Edlow A. G. (2021). Maternal metabolic disease and offspring neurodevelopment—an evolving public health crisis. *JAMA Netw. Open* 4 e2129674. 10.1001/jamanetworkopen.2021.29674 34648016

[B121] Ehses S. Raschke I. Mancuso G. Bernacchia A. Geimer S. Tondera D. (2009). Regulation of OPA1 processing and mitochondrial fusion by m-AAA protease isoenzymes and OMA1. *J. Cell Biol.* 187 1023–1036. 10.1083/jcb.200906084 20038678 PMC2806285

[B122] Eiraku M. Watanabe K. Matsuo-Takasaki M. Kawada M. Yonemura S. Matsumura M. (2008). Self-organized formation of polarized cortical tissues from escs and its active manipulation by extrinsic signals. *Cell Stem Cell* 3 519–532. 10.1016/j.stem.2008.09.002 18983967

[B123] Elkabetz Y. Panagiotakos G. Al Shamy G. Socci N. D. Tabar V. Studer L. (2008). Human ES cell-derived neural rosettes reveal a functionally distinct early neural stem cell stage. *Genes Dev.* 22 152–165. 10.1101/gad.1616208 18198334 PMC2192751

[B124] Empie K. Rangarajan V. Juul S. E. (2015). Is the ferret a suitable species for studying perinatal brain injury? *Int. J. Dev. Neurosci.* 45 2–10. 10.1016/j.ijdevneu.2015.06.005 26102988 PMC4793918

[B125] Englund C. (2005). Pax6, Tbr2, and Tbr1 Are expressed sequentially by radial glia, intermediate progenitor cells, and postmitotic neurons in developing neocortex. *J. Neurosci.* 25 247–251. 10.1523/JNEUROSCI.2899-04.2005 15634788 PMC6725189

[B126] Eraso-Pichot A. Brasó-Vives M. Golbano A. Menacho C. Claro E. Galea E. (2018). GSEA of mouse and human mitochondriomes reveals fatty acid oxidation in astrocytes. *Glia* 66 1724–1735. 10.1002/glia.23330 29575211

[B127] Espuny-Camacho I. Michelsen K. A. Gall D. Linaro D. Hasche A. Bonnefont J. (2013). Pyramidal neurons derived from human pluripotent stem cells integrate efficiently into mouse brain circuits in vivo. *Neuron* 77 440–456. 10.1016/j.neuron.2012.12.011 23395372

[B128] Evans M. J. Kaufman M. H. (1981). Establishment in culture of pluripotential cells from mouse embryos. *Nature* 292 154–156. 10.1038/292154a0 7242681

[B129] Faitg J. Lacefield C. Davey T. White K. Laws R. Kosmidis S. (2021). 3D neuronal mitochondrial morphology in axons, dendrites, and somata of the aging mouse hippocampus. *Cell Rep.* 36:109509. 10.1016/j.celrep.2021.109509 34380033 PMC8423436

[B130] Fame R. M. Shannon M. L. Chau K. F. Head J. P. Lehtinen M. K. (2019). A concerted metabolic shift in early forebrain alters the CSF proteome and depends on MYC downregulation for mitochondrial maturation. *Development* 146:dev182857. 10.1242/dev.182857 31575649 PMC6826040

[B131] Fan X. Fu Y. Zhou X. Sun L. Yang M. Wang M. (2020). Single-cell transcriptome analysis reveals cell lineage specification in temporal-spatial patterns in human cortical development. *Sci. Adv.* 6:eaaz2978. 10.1126/sciadv.aaz2978 32923614 PMC7450478

[B132] Fang D. Yan S. Yu Q. Chen D. Yan S. S. (2016). Mfn2 is required for mitochondrial development and synapse formation in human induced pluripotent stem cells/hiPSC derived cortical neurons. *Sci. Rep.* 6 1–13. 10.1038/srep31462 27535796 PMC4989148

[B133] Farahany N. A. Greely H. T. Hyman S. Koch C. Grady C. Pasca S. P. (2018). The ethics of experimenting with human brain tissue comment. *Nature* 556 429–432. 10.1038/d41586-018-04813-x 29691509 PMC6010307

[B134] Fecher C. Trovò L. Müller S. A. Snaidero N. Wettmarshausen J. Heink S. (2019). Cell-type-specific profiling of brain mitochondria reveals functional and molecular diversity. *Nat. Neurosci.* 22 1731–1742. 10.1038/s41593-019-0479-z 31501572

[B135] Fedorova V. Vanova T. Elrefae L. Pospisil J. Petrasova M. Kolajova V. (2019). Differentiation of neural rosettes from human pluripotent stem cells in vitro is sequentially regulated on a molecular level and accomplished by the mechanism reminiscent of secondary neurulation. *Stem Cell Res.* 40 101563. 10.1016/j.scr.2019.101563 31494448

[B136] Feeney C. L. Lim A. Z. Fagan E. Blain A. Bright A. Maddison J. (2019). A case-comparison study of pregnant women with mitochondrial disease – what to expect? *BJOG Int. J. Obstet. Gynaecol.* 126 1380–1389. 10.1111/1471-0528.15667 30801962 PMC6767368

[B137] Ferlin T. Landrieu P. Rambaud C. Fernandez H. Dumoulin R. Rustin P. (1997). Segregation of the G8993 mutant mitochondrial DNA through generations and embryonic tissues family at risk of Leigh syndrome. *J. Pediatr.* 131 447–449. 10.1016/S0022-3476(97)80074-1 9329425

[B138] Ferrari M. Jain I. H. Goldberger O. Rezoagli E. Thoonen R. Chen K.-H. (2017). Hypoxia treatment reverses neurodegenerative disease in a mouse model of Leigh syndrome. *Proc. Natl. Acad. Sci. U.S.A.* 114 E4241–E4250. 10.1073/pnas.1621511114 28483998 PMC5448167

[B139] Fietz S. A. Kelava I. Vogt J. Wilsch-Bräuninger M. Stenzel D. Fish J. L. (2010). OSVZ progenitors of human and ferret neocortex are epithelial-like and expand by integrin signaling. *Nat. Neurosci.* 13 690–699. 10.1038/nn.2553 20436478

[B140] Finsterer J. (2008). Leigh and leigh-like syndrome in children and adults. *Pediatr. Neurol.* 39 223–235. 10.1016/j.pediatrneurol.2008.07.013 18805359

[B141] Fishell G. Kriegstein A. R. (2003). Neurons from radial glia: the consequences of asymmetric inheritance. *Curr. Opin. Neurobiol.* 13 34–41. 10.1016/S0959-4388(03)00013-8 12593980

[B142] Fogo G. M. Anzell A. R. Maheras K. J. Raghunayakula S. Wider J. M. Emaus K. J. (2021). Machine learning-based classification of mitochondrial morphology in primary neurons and brain. *Sci. Rep.* 11 1–12. 10.1038/s41598-021-84528-8 33664336 PMC7933342

[B143] Forbes C. E. Grafman J. (2010). The role of the human prefrontal cortex in social cognition and moral judgment. *Annu. Rev. Neurosci.* 33 299–324. 10.1146/annurev-neuro-060909-153230 20350167

[B144] Fort L. Gama V. Macara I. G. (2021). Apoptotic find-me signals are an essential driver of stem cell conversion to the cardiac lineage. *bioRxiv* [Preprint] bioRxiv: 2021.06.21.449262, 10.1101/2021.06.21.449262

[B145] Frade J. M. (2002). Interkinetic nuclear movement in the vertebrate neuroepithelium: encounters with an old acquaintance. *Prog. Brain Res.* 136 67–71. 10.1016/S0079-6123(02)36007-2 12143404

[B146] Franco P. G. Paganelli A. R. Löpez S. L. Carrasco A. E. (1999). Functional association of retinoic acid and hedgehog signaling in *Xenopus* primary neurogenesis. *Development* 126 4257–4265. 10.1242/dev.126.19.4257 10477294

[B147] Franco S. J. Gil-Sanz C. Martinez-Garay I. Espinosa A. Harkins-Perry S. R. Ramos C. (2012). Fate-restricted neural progenitors in the mammalian cerebral cortex. *Science* 337 746–749. 10.1126/science.1223616 22879516 PMC4287277

[B148] Frantz G. D. McConnell S. K. (1996). Restriction of late cerebral cortical progenitors to an upper-layer fate. *Neuron* 17 55–61. 10.1016/S0896-6273(00)80280-9 8755478

[B149] Frantz G. D. Weimann J. M. Levin M. E. McConnell S. K. (1994). Otx1 and Otx2 define layers and regions in developing cerebral cortex and cerebellum. *J. Neurosci.* 14 5725–5740. 10.1523/jneurosci.14-10-05725.1994 7931541 PMC6577005

[B150] Fünfschilling U. Supplie L. M. Mahad D. Boretius S. Saab A. S. Edgar J. (2012). Glycolytic oligodendrocytes maintain myelin and long-term axonal integrity. *Nature* 485 517–521. 10.1038/nature11007 22622581 PMC3613737

[B151] Fuster J. M. (2002). Frontal lobe and cognitive development. *J. Neurocytol.* 31 373–385. 10.1023/A:1024190429920 12815254

[B152] Gaiano N. Kohtz J. D. Turnbull D. H. Fishell G. (1999). A method for rapid gain-of-function studies in the mouse embryonic nervoussystem. *Nat. Neurosci.* 2 812–819. 10.1038/12186 10461220

[B153] Galera-Monge T. Zurita-Díaz F. González-Páramos C. Moreno-Izquierdo A. Fraga M. F. Fernández A. F. (2016). Generation of a human iPSC line from a patient with Leigh syndrome caused by a mutation in the MT-ATP6 gene. *Stem Cell Res.* 16 766–769. 10.1016/j.scr.2016.04.012 27346203

[B154] Gandre-Babbe S. Van Der Bliek A. M. (2008). The novel tail-anchored membrane protein Mff controls mitochondrial and peroxisomal fission in mammalian cells. *Mol. Biol. Cell* 19 2402–2412. 10.1091/mbc.E07-12-1287 18353969 PMC2397315

[B155] García-Marqués J. López-Mascaraque L. (2013). Clonal identity determines astrocyte cortical heterogeneity. *Cereb. Cortex* 23 1463–1472. 10.1093/cercor/bhs134 22617854

[B156] Garnett A. T. Square T. A. Medeiros D. M. (2012). BMP, WNT and FGF signals are integrated through evolutionarily conserved enhancers to achieve robust expression of Pax3 and Zic genes at the zebrafish neural plate border. *Development* 139 4220–4231. 10.1242/dev.081497 23034628 PMC4074300

[B157] Gaspard N. Bouschet T. Hourez R. Dimidschstein J. Naeije G. Van Den Ameele J. (2008). An intrinsic mechanism of corticogenesis from embryonic stem cells. *Nature* 455 351–357. 10.1038/nature07287 18716623

[B158] Ge W. P. Miyawaki A. Gage F. H. Jan Y. N. Jan L. Y. (2012). Local generation of glia is a major astrocyte source in postnatal cortex. *Nature* 484 376–380. 10.1038/nature10959 22456708 PMC3777276

[B159] Gerrard L. Rodgers L. Cui W. (2005). Differentiation of human embryonic stem cells to neural lineages in adherent culture by blocking bone morphogenetic protein signaling. *Stem Cells* 23 1234–1241. 10.1634/stemcells.2005-0110 16002783

[B160] Geschwind D. H. Flint J. (2015). Genetics and genomics of psychiatric disease. *Science* 349 1489–1494. 10.1126/science.aaa8954 26404826 PMC4694563

[B161] Giacomello M. Pyakurel A. Glytsou C. Scorrano L. (2020). The cell biology of mitochondrial membrane dynamics. *Nat. Rev. Mol. Cell Biol.* 21 204–224. 10.1038/s41580-020-0210-7 32071438

[B162] Giandomenico S. L. Mierau S. B. Gibbons G. M. Wenger L. M. D. Masullo L. Sit T. (2019). Cerebral organoids at the air–liquid interface generate diverse nerve tracts with functional output. *Nat. Neurosci.* 22 669–679. 10.1038/s41593-019-0350-2 30886407 PMC6436729

[B163] Goda N. Kanai M. (2012). Hypoxia-inducible factors and their roles in energy metabolism. *Int. J. Hematol.* 95 457–463. 10.1007/s12185-012-1069-y 22535382

[B164] Gonzalez F. F. Miller S. P. (2006). Does perinatal asphyxia impair cognitive function without cerebral palsy? *Arch. Dis. Child. Fetal Neonatal Ed.* 91 F454–F459. 10.1136/adc.2005.092445 17056843 PMC2672766

[B165] Gordon A. Yoon S.-J. Tran S. S. Makinson C. D. Park J. Y. Andersen J. (2021). Long-term maturation of human cortical organoids matches key early postnatal transitions. *Nat. Neurosci.* 24 331–342. 10.1038/s41593-021-00802-y 33619405 PMC8109149

[B166] Gorman G. S. Chinnery P. F. DiMauro S. Hirano M. Koga Y. McFarland R. (2016). Mitochondrial diseases. *Nat. Rev. Dis. Prim.* 2 1–22. 10.1038/nrdp.2016.80 27775730

[B167] Götz M. Barde Y. A. (2005). Radial glial cells: defined and major intermediates between embryonicstem cells and CNS neurons. *Neuron* 46 369–372. 10.1016/j.neuron.2005.04.012 15882633

[B168] Götz M. Huttner W. B. (2005). The cell biology of neurogenesis. *Nat. Rev. Mol. Cell Biol.* 6 777–788. 10.1038/nrm1739 16314867

[B169] Götz M. Stoykova A. Gruss P. (1998). Pax6 controls radial glia differentiation in the cerebral cortex. *Neuron* 21 1031–1044. 10.1016/S0896-6273(00)80621-2 9856459

[B170] Grabiec M. Høíbková H. Vaøecha M. Støítecká D. Hampl A. Dvoøák P. (2016). Stage-specific roles of FGF2 signaling in human neural development. *Stem Cell Res.* 17 330–341. 10.1016/j.scr.2016.08.012 27608170

[B171] Grace H. E. Galdun P. Lesnefsky E. J. West F. D. Iyer S. (2019). mRNA reprogramming of t8993g leigh’s syndrome fibroblast cells to create induced pluripotent stem cell models for mitochondrial disorders. *Stem Cells Dev.* 28 846–859. 10.1089/scd.2019.0045 31017045 PMC6602115

[B172] Greely H. T. Ramos K. M. Grady C. (2016). Neuroethics in the age of brain projects. *Neuron* 92 637–641. 10.1016/j.neuron.2016.10.048 27810008

[B173] Green D. R. (2000). Apoptotic pathways: paper wraps stone blunts scissors. *Cell* 102 1–4. 10.1016/S0092-8674(00)00003-9 10929706

[B174] Greig L. C. Woodworth M. B. Galazo M. J. Padmanabhan H. Macklis J. D. (2013). Molecular logic of neocortical projection neuron specification, development and diversity. *Nat. Rev. Neurosci.* 14 755–769. 10.1038/nrn3586 24105342 PMC3876965

[B175] Grier J. Hirano M. Karaa A. Shepard E. Thompson J. L. P. (2018). Diagnostic odyssey of patients with mitochondrial disease. *Neurol. Genet.* 4:e230. 10.1212/NXG.0000000000000230 29600276 PMC5873725

[B176] Griffin E. E. Graumann J. Chan D. C. (2005). The WD40 protein Caf4p is a component of the mitochondrial fission machinery and recruits Dnm1p to mitochondria. *J. Cell Biol.* 170 237–248. 10.1083/jcb.200503148 16009724 PMC2171414

[B177] Grivennikova V. G. Kareyeva A. V. Vinogradov A. D. (2010). What are the sources of hydrogen peroxide production by heart mitochondria? *Biochim. Biophys. Acta Bioenerg.* 1797 939–944. 10.1016/j.bbabio.2010.02.013 20170624 PMC2891298

[B178] Grohm J. Kim S. W. Mamrak U. Tobaben S. Cassidy-Stone A. Nunnari J. (2012). Inhibition of Drp1 provides neuroprotection in vitro and in vivo. *Cell Death Differ.* 19 1446–1458. 10.1038/cdd.2012.18 22388349 PMC3422469

[B179] Grow W. A. (2018). “Development of the nervous system,” in *Fundamental Neuroscience for Basic and Clinical Applications*, 5th Edn, eds Haines D. E. Mihailoff G. A. (Amsterdam: Elsevier Inc), 72–90.e1. 10.1016/B978-0-323-39632-5.00005-0.

[B180] Halfter W. Dong S. Yip Y. P. Willem M. Mayer U. (2002). A critical function of the pial basement membrane in cortical histogenesis. *J. Neurosci.* 22 6029–6040. 10.1523/jneurosci.22-14-06029.2002 12122064 PMC6757907

[B181] Hallin U. Kondo E. Ozaki Y. Hagberg H. Shibasaki F. Blomgren K. (2006). Bcl-2 phosphorylation in the BH4 domain precedes caspase-3 activation and cell death after neonatal cerebral hypoxic-ischemic injury. *Neurobiol. Dis.* 21 478–486. 10.1016/j.nbd.2005.08.013 16213739

[B182] Han W. Kwan K. Y. Shim S. Lam M. M. S. Shin Y. Xu M. (2011). TBR1 directly represses Fezf2 to control the laminar origin and development of the corticospinal tract. *Proc. Natl. Acad. Sci. U.S.A.* 108 3041–3046. 10.1073/pnas.1016723108 21285371 PMC3041103

[B183] Han X. J. Lu Y. F. Li S. A. Kaitsuka T. Sato Y. Tomizawa K. (2008). CaM kinase Iα-induced phosphorylation of Drp1 regulates mitochondrial morphology. *J. Cell Biol.* 182 573–585. 10.1083/jcb.200802164 18695047 PMC2500141

[B184] Hanashima C. Li S. C. Shen L. Lai E. Fishell G. (2004). Foxg1 suppresses early cortical cell fate. *Science* 303 56–59. 10.1126/science.1090674 14704420

[B185] Handel A. E. Chintawar S. Lalic T. Whiteley E. Vowles J. Giustacchini A. (2016). Assessing similarity to primary tissue and cortical layer identity in induced pluripotent stem cell-derived cortical neurons through single-cell transcriptomics. *Hum. Mol. Genet.* 25 989–1000. 10.1093/hmg/ddv637 26740550 PMC4754051

[B186] Hansen D. V. Lui J. H. Parker P. R. L. Kriegstein A. R. (2010). Neurogenic radial glia in the outer subventricular zone of human neocortex. *Nature* 464 554–561. 10.1038/nature08845 20154730

[B187] Hara Y. Yuk F. Puri R. Janssen W. G. M. M. Rapp P. R. Morrison J. H. (2014). Presynaptic mitochondrial morphology in monkey prefrontal cortex correlates with working memory and is improved with estrogen treatment. *Proc. Natl. Acad. Sci. U.S.A.* 111 486–491. 10.1073/pnas.1311310110 24297907 PMC3890848

[B188] Harding A. E. Holt I. J. Sweeney M. G. Brockington M. Davis M. B. (1992). Prenatal diagnosis of mitochondrial DNA8993 T→G disease. *Am. J. Hum. Genet.* 50 629–633. 1539598 PMC1684296

[B189] Haremaki T. Metzger J. J. Rito T. Ozair M. Z. Etoc F. Brivanlou A. H. (2019). Self-organizing neuruloids model developmental aspects of Huntington’s disease in the ectodermal compartment. *Nat. Biotechnol.* 37 1198–1208. 10.1038/s41587-019-0237-5 31501559

[B190] Hartfuss E. Galli R. Heins N. Götz M. (2001). Characterization of CNS precursor subtypes and radial glia. *Dev. Biol.* 229 15–30. 10.1006/dbio.2000.9962 11133151

[B191] Hattori T. Hamazaki T. Kudo S. Shintaku H. (2016). Metabolic signature of MELAS/LEIGH overlap syndrome in patient-specific induced pluripotent stem cells model. *Osaka City Med. J.* 62 69–76. 30721581

[B192] Haubensak W. Attardo A. Denk W. Huttner W. B. (2004). Neurons arise in the basal neuroepithelium of the early mammalian telencephalon: a major site of neurogenesis. *Proc. Natl. Acad. Sci. U.S.A.* 101 3196–3201. 10.1073/pnas.0308600100 14963232 PMC365766

[B193] Hay E. D. (2005). The mesenchymal cell, its role in the embryo, and the remarkable signaling mechanisms that create it. *Dev. Dyn.* 233 706–720. 10.1002/dvdy.20345 15937929

[B194] Head B. Griparic L. Amiri M. Gandre-Babbe S. Van Der Bliek A. M. (2009). Inducible proteolytic inactivation of OPA1 mediated by the OMA1 protease in mammalian cells. *J. Cell Biol.* 187 959–966. 10.1083/jcb.200906083 20038677 PMC2806274

[B195] Heide M. Haffner C. Murayama A. Kurotaki Y. Shinohara H. Okano H. (2020). Human-specific ARHGAP11B increases size and folding of primate neocortex in the fetal marmoset. *Science* 369 546–550. 10.1126/science.abb2401 32554627

[B196] Heins N. Malatesta P. Cecconi F. Nakafuku M. Tucker K. L. Hack M. A. (2002). Glial cells generate neurons: the role of the transcription factor Pax6. *Nat. Neurosci.* 5 308–315. 10.1038/nn828 11896398

[B197] Herculano-Houzel S. Catania K. Manger P. R. Kaas J. H. (2015). Mammalian brains are made of these: a dataset of the numbers and densities of neuronal and nonneuronal cells in the brain of glires, primates, scandentia, eulipotyphlans, afrotherians and artiodactyls, and their relationship with body mass. *Brain. Behav. Evol.* 86 145–163. 10.1159/000437413 26418466

[B198] Herculano-Houzel S. Kaas J. H. de Oliveira-Souza R. (2016). Corticalization of motor control in humans is a consequence of brain scaling in primate evolution. *J. Comp. Neurol.* 524 448–455. 10.1002/cne.23792 25891512

[B199] Hevner R. F. Shi L. Justice N. Hsueh Y. P. Sheng M. Smiga S. (2001). Tbr1 regulates differentiation of the preplate and layer 6. *Neuron* 29 353–366. 10.1016/S0896-6273(01)00211-2 11239428

[B200] Hiesberger T. Trommsdorff M. Howell B. W. Goffinet A. Mumby M. C. Cooper J. A. (1999). Direct binding of Reelin to VLDL receptor and ApoE receptor 2 induces tyrosine phosphorylation of Disabled-1 and modulates tau phosphorylation. *Neuron* 24 481–489. 10.1016/S0896-6273(00)80861-2 10571241

[B201] His W. (1889). Die Neuroblasten und deren entstehung im embryonal marke. *Abh. Math. Phys. Cl. Kgl. Sach. Ges. Wiss.* 15 313–372.

[B202] Hoerder-Suabedissen A. Molnár Z. (2015). Development, evolution and pathology of neocortical subplate neurons. *Nat. Rev. Neurosci.* 16 133–146. 10.1038/nrn3915 25697157

[B203] Hoffman G. E. Hartley B. J. Flaherty E. Ladran I. Gochman P. Ruderfer D. M. (2017). Transcriptional signatures of schizophrenia in hiPSC-derived NPCs and neurons are concordant with post-mortem adult brains. *Nat. Commun.* 8 1–15. 10.1038/s41467-017-02330-5 29263384 PMC5738408

[B204] Hoffman G. E. Schrode N. Flaherty E. Brennand K. J. (2019). New considerations for hiPSC-based models of neuropsychiatric disorders. *Mol. Psychiatry* 24 49–66. 10.1038/s41380-018-0029-1 29483625 PMC6109625

[B205] Homem C. C. F. Steinmann V. Burkard T. R. Jais A. Esterbauer H. Knoblich J. A. (2014). Ecdysone and mediator change energy metabolism to terminate proliferation in drosophila neural stem cells. *Cell* 158 874–888. 10.1016/j.cell.2014.06.024 25126791

[B206] Hoppins S. Lackner L. Nunnari J. (2007). The machines that divide and fuse mitochondria. *Annu. Rev. Biochem.* 76 751–780. 10.1146/annurev.biochem.76.071905.090048 17362197

[B207] Hříbková H. Grabiec M. Klemová D. Slaninová I. Sun Y.-M. (2018). Calcium signaling mediates five types of cell morphological changes to form neural rosettes. *J. Cell Sci.* 131:jcs206896. 10.1242/jcs.206896 29361526

[B208] Hsu S. Y. Kaipia A. McGee E. Lomeli M. Hsueh A. J. W. (1997). Bok is a pro-apoptotic Bcl-2 protein with restricted expression in reproductive tissues and heterodimerizes with selective anti-apoptotic Bcl-2 family members. *Proc. Natl. Acad. Sci. U.S.A.* 94 12401–12406. 10.1073/pnas.94.23.12401 9356461 PMC24966

[B209] Huang W. Bhaduri A. Velmeshev D. Wang S. Wang L. Rottkamp C. A. (2020). Origins and proliferative states of human oligodendrocyte precursor cells. *Cell* 182 594–608.e11. 10.1016/j.cell.2020.06.027 32679030 PMC7415734

[B210] Hutsler J. J. Lee D. G. Porter K. K. (2005). Comparative analysis of cortical layering and supragranular layer enlargement in rodent carnivore and primate species. *Brain Res.* 1052 71–81. 10.1016/j.brainres.2005.06.015 16018988

[B211] Huttenlocher P. R. (1979). Synaptic density in human frontal cortex – developmental changes and effects of aging. *Brain Res.* 163 195–205. 10.1016/0006-8993(79)90349-4 427544

[B212] Huttner W. B. Brand M. (1997). Asymmetric division and polarity of neuroepithelial cells. *Curr. Opin. Neurobiol.* 7 29–39. 10.1016/S0959-4388(97)80117-1 9039800

[B213] Inak G. Lorenz C. Lisowski P. Zink A. Mlody B. Prigione A. (2017). Concise review: induced pluripotent stem cell-based drug discovery for mitochondrial disease. *Stem Cells* 35 1655–1662. 10.1002/stem.2637 28544378

[B214] Inak G. Rybak-Wolf A. Lisowski P. Pentimalli T. M. Jüttner R. Glažar P. (2021). Defective metabolic programming impairs early neuronal morphogenesis in neural cultures and an organoid model of Leigh syndrome. *Nat. Commun.* 12 1–22. 10.1038/s41467-021-22117-z 33771987 PMC7997884

[B215] Ingerman E. Perkins E. M. Marino M. Mears J. A. McCaffery J. M. Hinshaw J. E. (2005). Dnm1 forms spirals that are structurally tailored to fit mitochondria. *J. Cell Biol.* 170 1021–1027. 10.1083/jcb.200506078 16186251 PMC2171542

[B216] Inohara N. Ekhterae D. Garcia I. Carrio R. Merino J. Merry A. (1998). Mtd, a novel Bcl-2 family member activates apoptosis in the absence of heterodimerization with Bcl-2 and Bcl-X(L). *J. Biol. Chem.* 273 8705–8710. 10.1074/jbc.273.15.8705 9535847

[B217] Inoue K. Terashima T. Nishikawa T. Takumi T. (2004). Fez1 is layer-specifically expressed in the adult mouse neocortex. *Eur. J. Neurosci.* 20 2909–2916. 10.1111/j.1460-9568.2004.03763.x 15579145

[B218] Ioannou M. S. Jackson J. Sheu S. H. Chang C. L. Weigel A. V. Liu H. (2019). Neuron-astrocyte metabolic coupling protects against activity-induced fatty acid toxicity. *Cell* 177 1522–1535.e14. 10.1016/j.cell.2019.04.001 31130380

[B219] Ishihara N. Nomura M. Jofuku A. Kato H. Suzuki S. O. Masuda K. (2009). Mitochondrial fission factor Drp1 is essential for embryonic development and synapse formation in mice. *Nat. Cell Biol.* 11 958–966. 10.1038/ncb1907 19578372

[B220] Iwata R. Casimir P. Vanderhaeghen P. (2020). Mitochondrial dynamics in postmitotic cells regulate neurogenesis. *Science* 369 858–862. 10.1126/science.aba9760 32792401

[B221] Jain I. H. Zazzeron L. Goldberger O. Marutani E. Wojtkiewicz G. R. Ast T. (2019). Leigh syndrome mouse model can be rescued by interventions that normalize brain hyperoxia, but not hif activation. *Cell Metab.* 30 824–832.e3. 10.1016/j.cmet.2019.07.006 31402314 PMC6903907

[B222] Jain I. H. Zazzeron L. Goli R. Alexa K. Schatzman-Bone S. Dhillon H. (2016). Hypoxia as a therapy for mitochondrial disease. *Science* 352 54–61. 10.1126/science.aad9642 26917594 PMC4860742

[B223] James D. I. Parone P. A. Mattenberger Y. Martinou J. C. (2003). hFis1, a novel component of the mammalian mitochondrial fission machinery. *J. Biol. Chem.* 278 36373–36379. 10.1074/jbc.M303758200 12783892

[B224] Jessell T. M. Dodd J. (1990). Floor plate-derived signals and the control of neural cell pattern in vertebrates. *Harvey Lect.* 86 87–128. 2152141

[B225] Ji F. Shen T. Zou W. Jiao J. (2017). UCP2 regulates embryonic neurogenesis via ros-mediated yap alternation in the developing neocortex. *Stem Cells* 35 1479–1492. 10.1002/stem.2605 28276603

[B226] Jones-Villeneuve E. M. Rudnicki M. A. Harris J. F. McBurney M. W. (1983). Retinoic acid-induced neural differentiation of embryonal carcinoma cells. *Mol. Cell. Biol.* 3 2271–2279. 10.1128/mcb.3.12.2271-2279.1983 6656766 PMC370098

[B227] Joshi P. Bodnya C. Rasmussen M. L. Romero-Morales A. I. Bright A. Gama V. (2020). Modeling the function of BAX and BAK in early human brain development using iPSC-derived systems. *Cell Death Dis.* 11:808. 10.1038/s41419-020-03002-x 32978370 PMC7519160

[B228] Journiac N. Gilabert-Juan J. Cipriani S. Benit P. Liu X. Jacquier S. (2020). Cell metabolic alterations due to mcph1 mutation in microcephaly. *Cell Rep.* 31:107506. 10.1016/j.celrep.2020.03.070 32294449

[B229] Jürgensmeier J. M. Xie Z. Deveraux Q. Ellerby L. Bredesen D. Reed J. C. (1998). Bax directly induces release of cytochrome c from isolated mitochondria. *Proc. Natl. Acad. Sci. U.S.A.* 95 4997–5002. 10.1073/pnas.95.9.4997 9560217 PMC20202

[B230] Kaas J. H. (2008). The evolution of the complex sensory and motor systems of the human brain. *Brain Res. Bull.* 75 384–390. 10.1016/j.brainresbull.2007.10.009 18331903 PMC2349093

[B231] Kadoshima T. Sakaguchi H. Nakano T. Soen M. Ando S. Eiraku M. (2013). Self-organization of axial polarity, inside-out layer pattern, and species-specific progenitor dynamics in human ES cell-derived neocortex. *Proc. Natl. Acad. Sci. U.S.A.* 110 20284–20289. 10.1073/pnas.1315710110 24277810 PMC3864329

[B232] Kalebic N. Huttner W. B. (2020). Basal progenitor morphology and neocortex evolution. *Trends Neurosci.* 43 843–853. 10.1016/j.tins.2020.07.009 32828546

[B233] Kan A. (2017). Machine learning applications in cell image analysis. *Immunol. Cell Biol.* 95 525–530. 10.1038/icb.2017.16 28294138

[B234] Kang P. Lee H. K. Glasgow S. M. Finley M. Donti T. Gaber Z. B. (2012). Sox9 and NFIA coordinate a transcriptional regulatory cascade during the initiation of gliogenesis. *Neuron* 74 79–94. 10.1016/j.neuron.2012.01.024 22500632 PMC3543821

[B235] Kanton S. Boyle M. J. He Z. Santel M. Weigert A. Sanchís-Calleja F. (2019). Organoid single-cell genomic atlas uncovers human-specific features of brain development. *Nature* 574 418–422. 10.1038/s41586-019-1654-9 31619793

[B236] Karbowski M. Lee Y. J. Gaume B. Jeong S. Y. Frank S. Nechushtan A. (2002). Spatial and temporal association of Bax with mitochondrial fission sites, Drp1, and Mfn2 during apoptosis. *J. Cell Biol.* 159 931–938. 10.1083/jcb.200209124 12499352 PMC2173996

[B237] Karbowski M. Norris K. L. Cleland M. M. Jeong S. Y. Youle R. J. (2006). Role of Bax and Bak in mitochondrial morphogenesis. *Nature* 443 658–662. 10.1038/nature05111 17035996

[B238] Ke F. F. S. Vanyai H. K. Cowan A. D. Delbridge A. R. D. Whitehead L. Grabow S. (2018). Embryogenesis and adult life in the absence of intrinsic apoptosis effectors BAX, BAK, and BOK. *Cell* 173 1217–1230.e17. 10.1016/j.cell.2018.04.036 29775594

[B239] Khacho M. Slack R. S. (2018). Mitochondrial dynamics in the regulation of neurogenesis: from development to the adult brain. *Dev. Dyn.* 247 47–53. 10.1002/dvdy.24538 28643345

[B240] Khacho M. Clark A. Svoboda D. S. Azzi J. MacLaurin J. G. Meghaizel C. (2016). Mitochondrial dynamics impacts stem cell identity and fate decisions by regulating a nuclear transcriptional program. *Cell Stem Cell.* 19 232–247. 10.1016/j.stem.2016.04.015 27237737

[B241] Khacho M. Harris R. Slack R. S. (2019). Mitochondria as central regulators of neural stem cell fate and cognitive function. *Nat. Rev. Neurosci.* 20 34–48. 10.1038/s41583-018-0091-3 30464208

[B242] Kiecker C. Niehrs C. (2001). A morphogen gradient of Wnt/β-catenin signalling regulates anteroposterior neural patterning in *Xenopus*. *Development* 128 4189–4201. 10.1242/dev.128.21.4189 11684656

[B243] Kim H. M. Qu T. Kriho V. Lacor P. Smalheiser N. Pappas G. D. (2002). Reelin function in neural stem cell biology. *Proc. Natl. Acad. Sci. U.S.A.* 99 4020–4025. 10.1073/pnas.062698299 11891343 PMC122641

[B244] Kitahara T. Sakaguchi H. Morizane A. Kikuchi T. Miyamoto S. Takahashi J. (2020). Axonal extensions along corticospinal tracts from transplanted human cerebral organoids. *Stem Cell Rep.* 15 467–481. 10.1016/j.stemcr.2020.06.016 32679062 PMC7419717

[B245] Klein Gunnewiek T. M. Van Hugte E. J. H. Frega M. Guardia G. S. Foreman K. Panneman D. (2020). m.3243A > G-induced mitochondrial dysfunction impairs human neuronal development and reduces neuronal network activity and synchronicity. *Cell Rep.* 31:107538. 10.1016/j.celrep.2020.107538 32320658

[B246] Kluck R. M. Bossy-Wetzel E. Green D. R. Newmeyer D. D. (1997). The release of cytochrome c from mitochondria: a primary site for Bcl- 2 regulation of apoptosis. *Science* 275 1132–1136. 10.1126/science.275.5303.1132 9027315

[B247] Kluck R. M. Degli Esposti M. Perkins G. Renken C. Kuwana T. Bossy-Wetzel E. (1999). The pro-apoptotic proteins, Bid and Bax, cause a limited permeabilization of the mitochondrial outer membrane that is enhanced by cytosol. *J. Cell Biol.* 147 809–822. 10.1083/jcb.147.4.809 10562282 PMC2156156

[B248] Knight G. T. Lundin B. F. Iyer N. Ashton L. M. T. Sethares W. A. Willett R. M. (2018). Engineering induction of singular neural rosette emergence within hPSC-derived tissues. *Elife* 7:e37549. 10.7554/eLife.37549 30371350 PMC6205811

[B249] Knudson C. M. Tung K. S. K. Tourtellotte W. G. Brown G. A. J. Korsmeyer S. J. (1995). Bax-deficient mice with lymphoid hyperplasia and male germ cell death. *Science* 270 96–99. 10.1126/science.270.5233.96 7569956

[B250] Koch J. Feichtinger R. G. Freisinger P. Pies M. Schrödl F. Iuso A. (2016). Disturbed mitochondrial and peroxisomal dynamics due to loss of MFF causes Leigh-like encephalopathy, optic atrophy and peripheral neuropathy. *J. Med. Genet.* 53 270–278. 10.1136/jmedgenet-2015-103500 26783368

[B251] Koch P. Opitz T. Steinbeck J. A. Ladewig J. Brüstle O. (2009). A rosette-type, self-renewing human ES cell-derived neural stem cell with potential for in vitro instruction and synaptic integration. *Proc. Natl. Acad. Sci. U.S.A.* 106 3225–3230. 10.1073/pnas.0808387106 19218428 PMC2651316

[B252] Koelliker A. (1896). Handbuch der Gewebelehre des Menschen. *J. Anat. Physiol.* 31 1–896. 10.1111/j.1460-9568.1997.tb01391.x 9058041

[B253] Kornack D. R. Rakic P. (1995). Radial and horizontal deployment of clonally related cells in the primate neocortex: relationship to distinct mitotic lineages. *Neuron* 15 311–321. 10.1016/0896-6273(95)90036-5 7646888

[B254] Kostovic I. Rakic P. (1990). Developmental history of the transient subplate zone in the visual and somatosensory cortex of the macaque monkey and human brain. *J. Comp. Neurol.* 297 441–470. 10.1002/cne.902970309 2398142

[B255] Kowalczyk T. Pontious A. Englund C. Daza R. A. M. Bedogni F. Hodge R. (2009). Intermediate neuronal progenitors (basal progenitors) produce pyramidal-projection neurons for all layers of cerebral cortex. *Cereb. Cortex* 19 2439–2450. 10.1093/cercor/bhn260 19168665 PMC2742596

[B256] Koyanagi-Aoi M. Ohnuki M. Takahashi K. Okita K. Noma H. Sawamura Y. (2013). Differentiation-defective phenotypes revealed by large-scale analyses of human pluripotent stem cells. *Proc. Natl. Acad. Sci. U.S.A.* 110 20569–20574. 10.1073/pnas.1319061110 24259714 PMC3870695

[B257] Kriegstein A. Alvarez-Buylla A. (2009). The glial nature of embryonic and adult neural stem cells. *Annu. Rev. Neurosci.* 32 149–184. 10.1146/annurev.neuro.051508.135600 19555289 PMC3086722

[B258] Kuan C.-Y. Y. Roth K. A. Flavell R. A. Rakic P. (2000). Mechanisms of programmed cell death in the developing brain. *Trends Neurosci.* 23 291–297. 10.1016/S0166-2236(00)01581-2 10856938

[B259] Kudin A. P. Bimpong-Buta N. Y. B. Vielhaber S. Elger C. E. Kunz W. S. (2004). Characterization of superoxide-producing sites in isolated brain mitochondria. *J. Biol. Chem.* 279 4127–4135. 10.1074/jbc.M310341200 14625276

[B260] Kumar R. Bukowski M. J. Wider J. M. Reynolds C. A. Calo L. Lepore B. (2016). Mitochondrial dynamics following global cerebral ischemia. *Mol. Cell. Neurosci.* 76 68–75. 10.1016/j.mcn.2016.08.010 27567688 PMC5056829

[B261] Kwan K. Y. Lam M. M. S. Krsnik Ž Kawasawa Y. I Lefebvre V. Šestan N. (2008). SOX5 postmitotically regulates migration, postmigratory differentiation, and projections of subplate and deep-layer neocortical neurons. *Proc. Natl. Acad. Sci. U.S.A.* 105 16021–16026. 10.1073/pnas.0806791105 18840685 PMC2572944

[B262] Labrousse A. M. Zappaterra M. D. Rube D. A. Van der Bliek A. M. (1999). C. elegans dynamin-related protein DRP-1 controls severing of the mitochondrial outer membrane. *Mol. Cell* 4 815–826. 10.1016/S1097-2765(00)80391-3 10619028

[B263] Lages Y. M. Nascimento J. M. Lemos G. A. Galina A. Castilho L. R. Rehen S. K. (2015). Low oxygen alters mitochondrial function and response to oxidative stress in human neural progenitor cells. *PeerJ* 2015:e1486. 10.7717/peerj.1486 26713239 PMC4690376

[B264] Lai T. Jabaudon D. Molyneaux B. J. Azim E. Arlotta P. Menezes J. R. L. (2008). SOX5 controls the sequential generation of distinct corticofugal neuron subtypes. *Neuron* 57 232–247. 10.1016/j.neuron.2007.12.023 18215621

[B265] Lake N. J. Compton A. G. Rahman S. Thorburn D. R. (2016). Leigh syndrome: One disorder, more than 75 monogenic causes. *Ann. Neurol.* 79 190–203. 10.1002/ana.24551 26506407

[B266] Lancaster M. A. Knoblich J. A. (2014). Generation of cerebral organoids from human pluripotent stem cells. *Nat. Protoc.* 9 2329–2340. 10.1038/nprot.2014.158 25188634 PMC4160653

[B267] Lancaster M. A. Corsini N. S. Wolfinger S. Gustafson E. H. Phillips A. W. Burkard T. R. (2017). Guided self-organization and cortical plate formation in human brain organoids. *Nat. Biotechnol.* 35 659–666. 10.1038/nbt.3906 28562594 PMC5824977

[B268] Lancaster M. A. Renner M. Martin C. A. Wenzel D. Bicknell L. S. Hurles M. E. (2013). Cerebral organoids model human brain development and microcephaly. *Nature* 501 373–379. 10.1038/nature12517 23995685 PMC3817409

[B269] Lee H. Al Shamy G. Elkabetz Y. Schofield C. M. Harrsion N. L. Panagiotakos G. (2007). Directed differentiation and transplantation of human embryonic stem cell-derived motoneurons. *Stem Cells* 25 1931–1939. 10.1634/stemcells.2007-0097 17478583

[B270] Lee K. J. Jessell T. M. (1999). The specification of dorsal cell fates in the vertebrate central nervous system. *Annu. Rev. Neurosci.* 22 261–294. 10.1146/annurev.neuro.22.1.261 10202540

[B271] Lee K. J. Mendelsohn M. Jessell T. M. (1998). Neuronal patterning by BMPs: a requirement for GDF7 in the generation of a discrete class of commissural interneurons in the mouse spinal cord. *Genes Dev.* 12 3394–3407. 10.1101/gad.12.21.3394 9808626 PMC317230

[B272] Leingärtner A. Richards L. J. Dyck R. H. Akazawa C. O’Leary D. D. M. (2003). Cloning and cortical expression of rat Emx2 and adenovirus-mediated overexpression to assess its regulation of area-specific targeting of thalamocortical axons. *Cereb. Cortex* 13 648–660. 10.1093/cercor/13.6.648 12764041

[B273] Leonard A. P. Cameron R. B. Speiser J. L. Wolf B. J. Peterson Y. K. Schnellmann R. G. (2015). Quantitative analysis of mitochondrial morphology and membrane potential in living cells using high-content imaging, machine learning, and morphological binning. *Biochim. Biophys. Acta Mol. Cell Res.* 1853 348–360. 10.1016/j.bbamcr.2014.11.002 25447550 PMC4289477

[B274] Leone D. P. Srinivasan K. Chen B. Alcamo E. McConnell S. K. (2008). The determination of projection neuron identity in the developing cerebral cortex. *Curr. Opin. Neurobiol.* 18 28–35. 10.1016/j.conb.2008.05.006 18508260 PMC2483251

[B275] Letts J. A. Sazanov L. A. (2017). Clarifying the supercomplex: the higher-order organization of the mitochondrial electron transport chain. *Nat. Struct. Mol. Biol.* 24 800–808. 10.1038/nsmb.3460 28981073

[B276] Levitt P. Rakic P. (1980). Immunoperoxidase localization of glial fibrillary acidic protein in radial glial cells and astrocytes of the developing rhesus monkey brain. *J. Comp. Neurol.* 193 815–840. 10.1002/cne.901930316 7002963

[B277] Lewis T. L. Kwon S. K. Lee A. Shaw R. Polleux F. (2018). MFF-dependent mitochondrial fission regulates presynaptic release and axon branching by limiting axonal mitochondria size. *Nat. Commun.* 9 1–15. 10.1038/s41467-018-07416-2 30479337 PMC6258764

[B278] Li H. Alavian K. N. Lazrove E. Mehta N. Jones A. Zhang P. (2013). A Bcl-x L -Drp1 complex regulates synaptic vesicle membrane dynamics during endocytosis. *Nat. Cell Biol.* 15 773–785. 10.1038/ncb2791 23792689 PMC3725990

[B279] Li H. Chen Y. Jones A. F. Sanger R. H. Collis L. P. Flannery R. (2008). Bcl-xL induces Drp1-dependent synapse formation in cultured hippocampal neurons. *Proc. Natl. Acad. Sci. U.S.A.* 105 2169–2174. 10.1073/pnas.0711647105 18250306 PMC2542873

[B280] Li X. J. Du Z. W. Zarnowska E. D. Pankratz M. Hansen L. O. Pearce R. A. (2005). Specification of motoneurons from human embryonic stem cells. *Nat. Biotechnol.* 23 215–221. 10.1038/nbt1063 15685164

[B281] Li X. J. Zhang X. Johnson M. A. Wang Z. B. LaVaute T. Zhang S. C. (2009). Coordination of sonic hedgehog and Wnt signaling determines ventral and dorsal telencephalic neuron types from human embryonic stem cells. *Development* 136 4055–4063. 10.1242/dev.036624 19906872 PMC2778748

[B282] Lieberman O. J. McGuirt A. F. Tang G. Sulzer D. (2019). Roles for neuronal and glial autophagy in synaptic pruning during development. *Neurobiol. Dis.* 122 49–63. 10.1016/j.nbd.2018.04.017 29709573 PMC6204314

[B283] Liem J. Jessell T. M. Briscoe J. (2000). Regulation of the neural patterning activity of sonic hedgehog by secreted BMP inhibitors expressed by notochord and somites. *Development* 127 4855–4866. 10.1242/dev.127.22.4855 11044400

[B284] Liesa M. Shirihai O. S. (2013). Mitochondrial dynamics in the regulation of nutrient utilization and energy expenditure. *Cell Metab.* 17 491–506. 10.1016/j.cmet.2013.03.002 23562075 PMC5967396

[B285] Lima J. P. M. Rayêe D. Silva-Rodrigues T. Pereira P. R. P. Mendonca A. P. M. Rodrigues-Ferreira C. (2018). Perinatal asphyxia and brain development: mitochondrial damage without anatomical or cellular losses. *Mol. Neurobiol.* 55 8668–8679. 10.1007/s12035-018-1019-7 29582399

[B286] Lindhurst M. J. Fiermonte G. Song S. Struys E. De Leonardis F. Schwartzberg P. L. (2006). Knockout of Slc25a19 causes mitochondrial thiamine pyrophosphate depletion, embryonic lethality, CNS malformations, and anemia. *Proc. Natl. Acad. Sci. U.S.A.* 103 15927–15932. 10.1073/pnas.0607661103 17035501 PMC1595310

[B287] Liu R. Chan D. C. (2015). The mitochondrial fssion receptor Mff selectively recruits oligomerized Drp1. *Mol. Biol. Cell* 26 4466–4477. 10.1091/mbc.E15-08-0591 26446846 PMC4666140

[B288] Liu Y. Liu H. Sauvey C. Yao L. Zarnowska E. D. Zhang S. C. (2013). Directed differentiation of forebrain GABA interneurons from human pluripotent stem cells. *Nat. Protoc.* 8 1670–1679. 10.1038/nprot.2013.106 23928500 PMC4121169

[B289] Lodato S. Molyneaux B. J. Zuccaro E. Goff L. A. Chen H. H. Yuan W. (2014). Gene co-regulation by Fezf2 selects neurotransmitter identity and connectivity of corticospinal neurons. *Nat. Neurosci.* 17 1046–1054. 10.1038/nn.3757 24997765 PMC4188416

[B290] LOEB Classical Library (2021). *Hippocrates HIPPOCRATES OF COS, Decorum.* Available online at: https://www.loebclassics.com/view/hippocrates_cos-sacred_disease/1923/pb_LCL148.141.xml?readMode=recto [Accessed June 28, 2021].

[B291] Lorenz C. Lesimple P. Bukowiecki R. Zink A. Inak G. Mlody B. (2017). Human iPSC-derived neural progenitors are an effective drug discovery model for neurological mtDNA disorders. *Cell Stem Cell* 20 659–674.e9. 10.1016/j.stem.2016.12.013 28132834

[B292] Lv X. Ren S.-Q. Zhang X.-J. Shen Z. Ghosh T. Xianyu A. (2019). TBR2 coordinates neurogenesis expansion and precise microcircuit organization via Protocadherin 19 in the mammalian cortex. *Nat. Commun.* 10:3946. 10.1038/s41467-019-11854-x 31477701 PMC6718393

[B293] Ma H. Folmes C. D. L. Wu J. Morey R. Mora-Castilla S. Ocampo A. (2015). Metabolic rescue in pluripotent cells from patients with mtDNA disease. *Nature* 524 234–238. 10.1038/nature14546 26176921

[B294] Magini G. (1888). Nouvelles recherches histologiques sur le cerveau du foetus. *Arch. Ital. Biol.* 10 384–387.

[B295] Malatesta P. Hack M. A. Hartfuss E. Kettenmann H. Klinkert W. Kirchhoff F. (2003). Neuronal or glial progeny: Regional differences in radial glia fate. *Neuron* 37 751–764. 10.1016/S0896-6273(03)00116-8 12628166

[B296] Malatesta P. Hartfuss E. Götz M. (2000). Isolation of radial glial cells by fluorescent-activated cell sorting reveals a neural lineage. *Development* 127 5253–5263. 10.1242/dev.127.24.5253 11076748

[B297] Manabe N. Hirai S. I. Imai F. Nakanishi H. Takai Y. Ohno S. (2002). Association of ASIP/mPAR-3 with adherens junctions of mouse neuroepithelial cells. *Dev. Dyn.* 225 61–69. 10.1002/dvdy.10139 12203721

[B298] Mariani J. Simonini M. V. Palejev D. Tomasini L. Coppola G. Szekely A. M. (2012). Modeling human cortical development in vitro using induced pluripotent stem cells. *Proc. Natl. Acad. Sci. U.S.A.* 109 12770–12775. 10.1073/pnas.1202944109 22761314 PMC3411972

[B299] Marin-Padilla M. (1978). Dual origin of the mammalian neocortex and evolution of the cortical plate. *Anat. Embryol. (Berl)* 152 109–126. 10.1007/BF00315920 637312

[B300] Marín-Padilla M. (2014). The mammalian neocortex new pyramidal neuron: a new conception. *Front. Neuroanat.* 7:51. 10.3389/fnana.2013.00051 24431992 PMC3880895

[B301] Maroof A. M. Keros S. Tyson J. A. Ying S. W. Ganat Y. M. Merkle F. T. (2013). Directed differentiation and functional maturation of cortical interneurons from human embryonic stem cells. *Cell Stem Cell* 12 559–572. 10.1016/j.stem.2013.04.008 23642365 PMC3681523

[B302] Martínez-Cerdeño V. Noctor S. C. Kriegstein A. R. (2006). The role of intermediate progenitor cells in the evolutionary expansion of the cerebral cortex. *Cereb. Cortex* 16 Suppl 1 i152–i161. 10.1093/cercor/bhk017 16766701

[B303] Martínez-Reyes I. Chandel N. S. (2020). Mitochondrial TCA cycle metabolites control physiology and disease. *Nat. Commun.* 11:102. 10.1038/s41467-019-13668-3 31900386 PMC6941980

[B304] Marton R. M. Miura Y. Sloan S. A. Li Q. Revah O. Levy R. J. (2019). Differentiation and maturation of oligodendrocytes in human three-dimensional neural cultures. *Nat. Neurosci.* 22 484–491. 10.1038/s41593-018-0316-9 30692691 PMC6788758

[B305] McEvilly R. J. Ortiz de Diaz M. Schonemann M. D. Hooshmand F. Rosenfeld M. G. (2002). Transcriptional regulation of cortical neuron migration by POU domain factors. *Science* 295 1528–1532. 10.1126/science.1067132 11859196

[B306] McGrew L. L. Lai C. J. Moon R. T. (1995). Specification of the anteroposterior neural axis through synergistic interaction of the wnt signaling cascade withnogginandfollistatin. *Dev. Biol.* 172 337–342. 10.1006/dbio.1995.0027 7589812

[B307] McKenna W. L. Betancourt J. Larkin K. A. Abrams B. Guo C. Rubenstein J. L. R. (2011). Tbr1 and Fezf2 regulate alternate corticofugal neuronal identities during neocortical development. *J. Neurosci.* 31 549–564. 10.1523/JNEUROSCI.4131-10.2011 21228164 PMC3276402

[B308] McTague A. Rossignoli G. Ferrini A. Barral S. Kurian M. A. (2021). Genome editing in IPSC-based neural systems: from disease models to future therapeutic strategies. *Front. Genome Ed.* 3:8. 10.3389/fgeed.2021.630600 34713254 PMC8525405

[B309] Mears J. A. Lackner L. L. Fang S. Ingerman E. Nunnari J. Hinshaw J. E. (2011). Conformational changes in Dnm1 support a contractile mechanism for mitochondrial fission. *Nat. Struct. Mol. Biol.* 18 20–27. 10.1038/nsmb.1949 21170049 PMC3059246

[B310] Meijer M. Rehbach K. Brunner J. W. Classen J. A. Lammertse H. C. A. van Linge L. A. (2019). A single-cell model for synaptic transmission and plasticity in human iPSC-derived neurons. *Cell Rep.* 27 2199–2211.e6. 10.1016/j.celrep.2019.04.058 31091456

[B311] Mekki-Dauriac S. Agius E. Kan P. Cochard P. (2002). Bone morphogenetic proteins negatively control oligodendrocyte precursor specification in the chick spinal cord. *Development* 129 5117–5130. 10.1242/dev.129.22.5117 12399304

[B312] Mertens J. Reid D. Lau S. Kim Y. Gage F. H. (2018). Aging in a dish: IPSC-derived and directly induced neurons for studying brain aging and age-related neurodegenerative diseases. *Annu. Rev. Genet.* 52 271–293. 10.1146/annurev-genet-120417-031534 30208291 PMC6415910

[B313] Meshrkey F. Cabrera Ayuso A. Rao R. R. Iyer S. (2021). Quantitative analysis of mitochondrial morphologies in human induced pluripotent stem cells for Leigh syndrome. *Stem Cell Res.* 57 102572. 10.1016/j.scr.2021.102572 34662843 PMC10332439

[B314] Miller D. J. Bhaduri A. Sestan N. Kriegstein A. (2019). Shared and derived features of cellular diversity in the human cerebral cortex. *Curr. Opin. Neurobiol.* 56 117–124. 10.1016/j.conb.2018.12.005 30677551 PMC6996583

[B315] Mione M. C. Cavanagh J. F. R. Harris B. Parnavelas J. G. (1997). Cell fate specification and symmetrical/asymmetrical divisions in the developing cerebral cortex. *J. Neurosci.* 17 2018–2929. 10.1523/jneurosci.17-06-02018.1997 9045730 PMC6793772

[B316] Mishra P. Carelli V. Manfredi G. Chan D. C. (2014). Proteolytic cleavage of Opa1 stimulates mitochondrial inner membrane fusion and couples fusion to oxidative phosphorylation. *Cell Metab.* 19 630–641. 10.1016/j.cmet.2014.03.011 24703695 PMC4018240

[B317] Miura Y. Li M. Y. Birey F. Ikeda K. Revah O. Thete M. V. (2020). Generation of human striatal organoids and cortico-striatal assembloids from human pluripotent stem cells. *Nat. Biotechnol.* 38 1421–1430. 10.1038/s41587-020-00763-w 33273741 PMC9042317

[B318] Miyata T. Kawaguchi A. Okano H. Ogawa M. (2001). Asymmetric inheritance of radial glial fibers by cortical neurons. *Neuron* 31 727–741. 10.1016/S0896-6273(01)00420-2 11567613

[B319] Molnár Z. Pollen A. (2014). How unique is the human neocortex? *Development* 141 11–16. 10.1242/dev.101279 24346696

[B320] Molnár Z. Clowry G. J. Šestan N. Alzu’bi A. Bakken T. Hevner R. F. (2019). New insights into the development of the human cerebral cortex. *J. Anat.* 235 432–451. 10.1111/joa.13055 31373394 PMC6704245

[B321] Molyneaux B. J. Arlotta P. Fame R. M. MacDonald J. L. MacQuarrie K. L. Macklis J. D. (2009). Novel subtype-specific genes identify distinct subpopulations of callosal projection neurons. *J. Neurosci.* 29 12343–12354. 10.1523/JNEUROSCI.6108-08.2009 19793993 PMC2776075

[B322] Molyneaux B. J. Arlotta P. Menezes J. R. L. Macklis J. D. (2007). Neuronal subtype specification in the cerebral cortex. *Nat. Rev. Neurosci.* 8 427–437. 10.1038/nrn2151 17514196

[B323] Monsoro-Burq A. H. Wang E. Harland R. (2005). Msx1 and Pax3 cooperate to mediate FGF8 and WNT signals during *Xenopus* neural crest induction. *Dev. Cell* 8 167–178. 10.1016/j.devcel.2004.12.017 15691759

[B324] Mozdy A. D. McCaffery J. M. Shaw J. M. (2000). Dnm1p GTPase-mediated mitochondrial fission is a multi-step process requiring the novel integral membrane component Fis1p. *J. Cell Biol.* 151 367–379. 10.1083/jcb.151.2.367 11038183 PMC2192649

[B325] Mujtaba T. Piper D. R. Kalyani A. Groves A. K. Lucero M. T. Rao M. S. (1999). Lineage-restricted neural precursors can be isolated from both the mouse neural tube and cultured ES cells. *Dev. Biol.* 214 113–127. 10.1006/dbio.1999.9418 10491261

[B326] Muñoz-Sanjuán I. Brivanlou A. H. (2002). Neural induction, the default model and embryonic stem cells. *Nat. Rev. Neurosci.* 3 271–280. 10.1038/nrn786 11967557

[B327] Murciano A. Zamora J. López-Sánchez J. Frade J. M. (2002). Interkinetic nuclear movement may provide spatial clues to the regulation of neurogenesis. *Mol. Cell. Neurosci.* 21 285–300. 10.1006/mcne.2002.1174 12401448

[B328] Namba T. Dóczi J. Pinson A. Xing L. Kalebic N. Wilsch-Bräuninger M. (2020). Human-specific ARHGAP11B acts in mitochondria to expand neocortical progenitors by glutaminolysis. *Neuron* 105 867–881.e9. 10.1016/j.neuron.2019.11.027 31883789

[B329] Namihira M. Kohyama J. Semi K. Sanosaka T. Deneen B. Taga T. (2009). Committed neuronal precursors confer astrocytic potential on residual neural precursor cells. *Dev. Cell* 16 245–255. 10.1016/j.devcel.2008.12.014 19217426

[B330] Nat R. Nilbratt M. Narkilahti S. Winblad B. Hovatta O. Nordberg A. (2007). Neurogenic neuroepithelial and radial glial cells generated from six human embryonic stem cell lines in serum-free suspension and adherent cultures. *Glia* 55 385–399. 10.1002/glia.20463 17152062

[B331] National Academies of Sciences Engineering and Medicine (2021). *The Emerging Field of Human Neural Organoids, Transplants, and Chimeras.* Washington, DC: National Academies Press, 10.17226/26078 33844487

[B332] Nesbitt V. Alston C. L. Blakely E. L. Fratter C. Feeney C. L. Poulton J. (2014). A national perspective on prenatal testing for mitochondrial disease. *Eur. J. Hum. Genet.* 22 1255–1259. 10.1038/ejhg.2014.35 24642831 PMC4200441

[B333] Nguyen V. H. Trout J. Connors S. A. Andermann P. Weinberg E. Mullins M. C. (2000). Dorsal and intermediate neuronal cell types of the spinal cord are established by a BMP signaling pathway. *Development* 127 1209–1220. 10.1242/dev.127.6.1209 10683174

[B334] Niatsetskaya Z. V. Sosunov S. A. Matsiukevich D. Utkina-Sosunova I. V. Ratner V. I. Starkov A. A. (2012). The oxygen free radicals originating from mitochondrial complex i contribute to oxidative brain injury following hypoxia-ischemia in neonatal mice. *J. Neurosci.* 32 3235–3244. 10.1523/jneurosci.6303-11.2012 22378894 PMC3296485

[B335] Nicholas C. R. Chen J. Tang Y. Southwell D. G. Chalmers N. Vogt D. (2013). Functional maturation of hPSC-derived forebrain interneurons requires an extended timeline and mimics human neural development. *Cell Stem Cell* 12 573–586. 10.1016/j.stem.2013.04.005 23642366 PMC3699205

[B336] Nieto M. Monuki E. S. Tang H. Imitola J. Haubst N. Khoury S. J. (2004). Expression of Cux-1 and Cux-2 in the subventricular zone and upper layers II-IV of the cerebral cortex. *J. Comp. Neurol.* 479 168–180. 10.1002/cne.20322 15452856

[B337] Noctor S. C. Flint A. C. Weissman T. A. Dammerman R. S. Kriegstein A. R. (2001). Neurons derived from radial glial cells establish radial units in neocortex. *Nature* 409 714–720. 10.1038/35055553 11217860

[B338] Noctor S. C. Martínez-Cerdeño V. Kriegstein A. R. (2008). Distinct behaviors of neural stem and progenitor cells underlie cortical neurogenesis. *J. Comp. Neurol.* 508 28–44. 10.1002/cne.21669 18288691 PMC2635107

[B339] Noguchi M. Kasahara A. (2017). Mitochondrial dynamics coordinate cell differentiation. *Biochem. Biophys. Res. Commun.* 500 59–64. 10.1016/j.bbrc.2017.06.094 28634072

[B340] Nonomura K. Yamaguchi Y. Hamachi M. Koike M. Uchiyama Y. Nakazato K. (2013). Local apoptosis modulates early mammalian brain development through the elimination of morphogen-producing cells. *Dev. Cell* 27 621–634. 10.1016/j.devcel.2013.11.015 24369835

[B341] Northington F. J. Flock D. L. Martin L. J. Ferriero D. M. (2001). Delayed neurodegeneration in neonatal rat thalamus after hypoxia-ischemia is apoptosis. *J. Neurosci.* 21 1931–1938. 10.1523/JNEUROSCI.21-06-01931.2001 11245678 PMC6762598

[B342] Nowakowski T. J. Bhaduri A. Pollen A. A. Alvarado B. Mostajo-Radji M. A. Di Lullo E. (2017). Spatiotemporal gene expression trajectories reveal developmental hierarchies of the human cortex. *Science* 358 1318–1323. 10.1126/science.aap8809 29217575 PMC5991609

[B343] O’Rahilly R. Müller F. (2005). *The Embryonic Human Brain: An Atlas of Developmental Stages*, 3rd Edn. Hoboken, NJ: John Wiley & Sons, Inc, 10.1002/0471973084

[B344] O’Rahilly R. Müller F. (2008). Significant features in the early prenatal development of the human brain. *Ann. Anat.* 190 105–118. 10.1016/j.aanat.2008.01.001 18356030

[B345] O’Rahilly R. Müller F. (2010). Developmental stages in human embryos: revised and new measurements. *Cells Tissues Organs* 192 73–84. 10.1159/000289817 20185898

[B346] Okabe S. Forsberg-Nilsson K. Spiro A. C. Segal M. McKay R. D. G. (1996). Development of neuronal precursor cells and functional postmitotic neurons from embryonic stem cells in vitro. *Mech. Dev.* 59 89–102. 10.1016/0925-4773(96)00572-2 8892235

[B347] Orentas D. M. Hayes J. E. Dyer K. L. Miller R. H. (1999). Sonic hedgehog signaling is required during the appearance of spinal cord oligodendrocyte precursors. *Development* 126 2419–2429. 10.1242/dev.126.11.2419 10226001

[B348] Ortiz-González X. R. (2021). Mitochondrial dysfunction: a common denominator in neurodevelopmental disorders? *Dev. Neurosci.* 43 222–229. 10.1159/000517870 34350863 PMC8440386

[B349] Osellame L. D. Singh A. P. Stroud D. A. Palmer C. S. Stojanovski D. Ramachandran R. (2016). Cooperative and independent roles of the Drp1 adaptors Mff, MiD49 and MiD51 in mitochondrial fission. *J. Cell Sci.* 129 2170–2181. 10.1242/jcs.185165 27076521 PMC6919635

[B350] Otera H. Wang C. Cleland M. M. Setoguchi K. Yokota S. Youle R. J. (2010). Mff is an essential factor for mitochondrial recruitment of Drp1 during mitochondrial fission in mammalian cells. *J. Cell Biol.* 191 1141–1158. 10.1083/jcb.201007152 21149567 PMC3002033

[B351] Otsuga D. Keegan B. R. Brisch E. Thatcher J. W. Hermann G. J. Bleazard W. (1998). The dynamin-related GTPase, Dnm1p, controls mitochondrial morphology in yeast. *J. Cell Biol.* 143 333–349. 10.1083/jcb.143.2.333 9786946 PMC2132834

[B352] Palmer C. S. Elgass K. D. Parton R. G. Osellame L. D. Stojanovski D. Ryan M. T. (2013). Adaptor proteins MiD49 and MiD51 can act independently of Mff and Fis1 in Drp1 recruitment and are specific for mitochondrial fission. *J. Biol. Chem.* 288 27584–27593. 10.1074/jbc.M113.479873 23921378 PMC3779755

[B353] Pang Z. P. Yang N. Vierbuchen T. Ostermeier A. Fuentes D. R. Yang T. Q. (2011). Induction of human neuronal cells by defined transcription factors. *Nature* 476 220–223. 10.1038/nature10202 21617644 PMC3159048

[B354] Pankratz M. T. Li X.-J. LaVaute T. M. Lyons E. A. Chen X. Zhang S.-C. (2007). Directed Neural differentiation of human embryonic stem cells via an obligated primitive anterior stage. *Stem Cells* 25 1511–1520. 10.1634/stemcells.2006-0707 17332508 PMC2743478

[B355] Parikh S. Goldstein A. Karaa A. Koenig M. K. Anselm I. Brunel-Guitton C. (2017). Patient care standards for primary mitochondrial disease: a consensus statement from the mitochondrial medicine society. *Genet. Med.* 19 1–18. 10.1038/gim.2017.107 28749475 PMC7804217

[B356] Paşca A. M. Park J. Y. Shin H. W. Qi Q. Revah O. Krasnoff R. (2019). Human 3D cellular model of hypoxic brain injury of prematurity. *Nat. Med.* 25 784–791. 10.1038/s41591-019-0436-0 31061540 PMC7020938

[B357] Paşca A. M. Sloan S. A. Clarke L. E. Tian Y. Makinson C. D. Huber N. (2015). Functional cortical neurons and astrocytes from human pluripotent stem cells in 3D culture. *Nat. Methods* 12 671–678. 10.1038/nmeth.3415 26005811 PMC4489980

[B358] Paşca S. P. (2018). The rise of three-dimensional human brain cultures. *Nature* 553 437–445. 10.1038/nature25032 29364288

[B359] Pereira S. L. Grãos M. Rodrigues A. S. Anjo S. I. Carvalho R. A. Oliveira P. J. (2013). Inhibition of mitochondrial complex iii blocks neuronal differentiation and maintains embryonic stem cell pluripotency. *PLoS One* 8:e82095. 10.1371/journal.pone.0082095 24312632 PMC3847032

[B360] Perrier A. L. Tabar V. Barberi T. Rubio M. E. Bruses J. Topf N. (2004). Derivation of midbrain dopamine neurons from human embryonic stem cells. *Proc. Natl. Acad. Sci. U.S.A.* 101 12543–12548. 10.1073/pnas.0404700101 15310843 PMC515094

[B361] Petanjek Z. Judaš M. Kostoviæ I. Uylings H. B. M. (2008). Lifespan alterations of basal dendritic trees of pyramidal neurons in the human prefrontal cortex: a layer-specific pattern. *Cereb. Cortex* 18 915–929. 10.1093/cercor/bhm124 17652464

[B362] Petanjek Z. Judaš M. Šimiæ G. Rašin M. R. Uylings H. B. M. Rakic P. (2011). Extraordinary neoteny of synaptic spines in the human prefrontal cortex. *Proc. Natl. Acad. Sci. U.S.A.* 108 13281–13286. 10.1073/pnas.1105108108 21788513 PMC3156171

[B363] Petryniak M. A. Potter G. B. Rowitch D. H. Rubenstein J. L. R. (2007). Dlx1 and Dlx2 control neuronal versus oligodendroglial cell fate acquisition in the developing forebrain. *Neuron* 55 417–433. 10.1016/j.neuron.2007.06.036 17678855 PMC2039927

[B364] Pfisterer U. Wood J. Nihlberg K. Hallgren O. Bjermer L. Westergren-Thorsson G. (2011b). Efficient induction of functional neurons from adult human fibroblasts. *Cell Cycle* 10 3311–3316. 10.4161/cc.10.19.17584 21934358

[B365] Pfisterer U. Kirkeby A. Torper O. Wood J. Nelander J. Dufour A. (2011a). Direct conversion of human fibroblasts to dopaminergic neurons. *Proc. Natl. Acad. Sci. U.S.A.* 108 10343–10348. 10.1073/pnas.1105135108 21646515 PMC3121829

[B366] Picard M. McEwen B. S. (2014). Mitochondria impact brain function and cognition. *Proc. Natl. Acad. Sci. U.S.A.* 111 7–8. 10.1073/pnas.1321881111 24367081 PMC3890847

[B367] Piccoli C. Ria R. Scrima R. Cela O. D’Aprile A. Boffoli D. (2005). Characterization of mitochondrial and extra-mitochondrial oxygen consuming reactions in human hematopoietic stem cells: Novel evidence of the occurrence of NAD(P)H oxidase activity. *J. Biol. Chem.* 280 26467–26476. 10.1074/jbc.M500047200 15883163

[B368] Pilaz L. J. Patti D. Marcy G. Ollier E. Pfister S. Douglas R. J. (2009). Forced G1-phase reduction alters mode of division, neuron number, and laminar phenotype in the cerebral cortex. *Proc. Natl. Acad. Sci. U.S.A.* 106 21924–21929. 10.1073/pnas.0909894106 19959663 PMC2788480

[B369] Pixley S. K. R. de Vellis J. (1984). Transition between immature radial glia and mature astrocytes studied with a monoclonal antibody to vimentin. *Dev. Brain Res.* 15 201–209. 10.1016/0165-3806(84)90097-X 6383523

[B370] Pliss L. Pentney R. J. Johnson M. T. Patel M. S. (2004). Biochemical and structural brain alterations in female mice with cerebral pyruvate dehydrogenase deficiency. *J. Neurochem.* 91 1082–1091. 10.1111/j.1471-4159.2004.02790.x 15569252

[B371] Pollen A. A. Nowakowski T. J. Chen J. Retallack H. Sandoval-Espinosa C. Nicholas C. R. (2015). Molecular identity of human outer radial glia during cortical development. *Cell* 163 55–67. 10.1016/j.cell.2015.09.004 26406371 PMC4583716

[B372] Povea-Cabello S. Villanueva-Paz M. Suárez-Rivero J. M. Álvarez-Córdoba M. Villalón-García I. Talaverón-Rey M. (2021). Advances in mt-tRNA mutation-caused mitochondrial disease modeling: patients’ brain in a dish. *Front. Genet.* 11:1642. 10.3389/fgene.2020.610764 33510772 PMC7835939

[B373] Praefcke G. J. K. McMahon H. T. (2004). The dynamin superfamily: universal membrane tubulation and fission molecules? *Nat. Rev. Mol. Cell Biol.* 5 133–147. 10.1038/nrm1313 15040446

[B374] Presidential Commission for the Study of Bioethical (2015). Gray Matters: Integrative Approaches for Neuroscience, Ethics, and Society. *Jahrbuch Wissenschaft Ethik* 19 305–326. 10.1515/jwiet-2015-0120

[B375] Pressler R. Auvin S. (2013). Comparison of brain maturation among species: an example in translational research suggesting the possible use of bumetanide in newborn. *Front. Neurol.* 4:36. 10.3389/fneur.2013.00036 23596438 PMC3625921

[B376] Prieto J. León M. Ponsoda X. Sendra R. Bort R. Ferrer-Lorente R. (2016). Early ERK1/2 activation promotes DRP1-dependent mitochondrial fission necessary for cell reprogramming. *Nat. Commun.* 7 1–13. 10.1038/ncomms11124 27030341 PMC4821885

[B377] Prigione A. Fauler B. Lurz R. Lehrach H. Adjaye J. (2010). The senescence-related mitochondrial/oxidative stress pathway is repressed in human induced pluripotent stem cells. *Stem Cells* 28 721–733. 10.1002/stem.404 20201066

[B378] Prozorovski T. Schulze-Topphoff U. Glumm R. Baumgart J. Schröter F. Ninnemann O. (2008). Sirt1 contributes critically to the redox-dependent fate of neural progenitors. *Nat. Cell Biol.* 10 385–394. 10.1038/ncb1700 18344989

[B379] Puka-Sundvall M. Gajkowska B. Cholewinski M. Blomgren K. Lazarewicz J. W. Hagberg H. (2000). Subcellular distribution of calcium and ultrastructural changes after cerebral hypoxia-ischemia in immature rats. *Dev. Brain Res.* 125 31–41. 10.1016/S0165-3806(00)00110-3 11154758

[B380] Qian L. Tcw J. (2021). Human iPSC-based modeling of central nerve system disorders for drug discovery. *Int. J. Mol. Sci.* 22 1–36. 10.3390/ijms22031203 33530458 PMC7865494

[B381] Qian X. Goderie S. K. Shen Q. Stern J. H. Temple S. (1998). Intrinsic programs of patterned cell lineages in isolated vertebrate CNS ventricular zone cells. *Development* 125 3143–3152. 10.1242/dev.125.16.3143 9671587

[B382] Qian X. Jacob F. Song M. M. Nguyen H. N. Song H. Ming G. L. (2018). Generation of human brain region–specific organoids using a miniaturized spinning bioreactor. *Nat. Protoc.* 13 565–580. 10.1038/nprot.2017.152 29470464 PMC6241211

[B383] Qian X. Nguyen H. N. Song M. M. Hadiono C. Ogden S. C. Hammack C. (2016). Brain-region-specific organoids using mini-bioreactors for modeling ZIKV exposure. *Cell* 165 1238–1254. 10.1016/j.cell.2016.04.032 27118425 PMC4900885

[B384] Qian X. Song H. Ming G. L. (2019). Brain organoids: advances, applications and challenges. *Development* 146:dev166074. 10.1242/dev.166074 30992274 PMC6503989

[B385] Quadrato G. Nguyen T. Macosko E. Z. Sherwood J. L. Yang S. M. Berger D. R. (2017). Cell diversity and network dynamics in photosensitive human brain organoids. *Nature* 545 48–53. 10.1038/nature22047 28445462 PMC5659341

[B386] Raedler E. Raedler A. (1978). Autoradiographic study of early neurogenesis in rat neocortex. *Anat. Embryol. (Berl)* 154 267–284. 10.1007/BF00345657 707818

[B387] Rafalski V. A. Mancini E. Brunet A. (2012). Energy metabolism and energy-sensing pathways in mammalian embryonic and adult stem cell fate. *J. Cell Sci.* 125 5597–5608. 10.1242/jcs.114827 23420198 PMC3575699

[B388] Rafelski S. M. (2013). Mitochondrial network morphology: building an integrative, geometrical view. *BMC Biol.* 11:71. 10.1186/1741-7007-11-71 23800141 PMC3691739

[B389] Raichle M. E. Gusnard D. A. (2002). Appraising the brain’s energy budget. *Proc. Natl. Acad. Sci. U.S.A.* 99 10237–10239. 10.1073/pnas.172399499 12149485 PMC124895

[B390] Rakic P. (1972). Mode of cell migration to the superficial layers of fetal monkey neocortex. *J. Comp. Neurol.* 145 61–83. 10.1002/cne.901450105 4624784

[B391] Rakic P. (1978). Neuronal migration and contact guidance in the primate telencephalon. *Postgrad. Med. J.* 54 Suppl 1 25–40. 364453

[B392] Rallu M. Machold R. Gaiano N. Corbin J. G. McMahon A. P. Fishell G. (2002). Dorsoventral patterning is established in the telencephalon of mutants lacking both Gli3 and Hedgehog signaling. *Development* 129 4963–4974. 10.1242/dev.129.21.4963 12397105

[B393] Ramonet D. Perier C. Recasens A. Dehay B. Bové J. Costa V. (2013). Optic atrophy 1 mediates mitochondria remodeling and dopaminergic neurodegeneration linked to complex i deficiency. *Cell Death Differ.* 20 77–85. 10.1038/cdd.2012.95 22858546 PMC3524632

[B394] Rao V. T. S. Khan D. Cui Q. L. Fuh S. C. Hossain S. Almazan G. (2017). Distinct age and differentiation-state dependent metabolic profiles of oligodendrocytes under optimal and stress conditions. *PLoS One* 12:e0182372. 10.1371/journal.pone.0182372 28792512 PMC5549710

[B395] Rash B. G. Duque A. Morozov Y. M. Arellano J. I. Micali N. Rakic P. (2019). Gliogenesis in the outer subventricular zone promotes enlargement and gyrification of the primate cerebrum. *Proc. Natl. Acad. Sci. U.S.A.* 116 7089–7094. 10.1073/pnas.1822169116 30894491 PMC6452694

[B396] Rasmussen M. L. Kline L. A. Park K. P. Ortolano N. A. Romero-Morales A. I. Anthony C. C. (2018). A non-apoptotic function of MCL-1 in promoting pluripotency and modulating mitochondrial dynamics in stem cells. *Stem Cell Rep.* 10 684–692. 10.1016/j.stemcr.2018.01.005 29429957 PMC5918190

[B397] Rasmussen M. L. Taneja N. Neininger A. C. Wang L. Robertson G. L. Riffle S. N. (2020). MCL-1 Inhibition by selective BH3 mimetics disrupts mitochondrial dynamics causing loss of viability and functionality of human cardiomyocytes. *iScience* 23:101015. 10.1016/j.isci.2020.101015 32283523 PMC7155208

[B398] Rastogi A. Joshi P. Contreras E. Gama V. (2019). Remodeling of mitochondrial morphology and function: an emerging hallmark of cellular reprogramming. *Cell Stress* 3 181–194. 10.15698/cst2019.06.189 31225513 PMC6558935

[B399] Reid C. B. Tavazoie S. F. Walsh C. A. (1997). Clonal dispersion and evidence for asymmetric cell division in ferret cortex. *Development* 124 2441–2450. 10.1242/dev.124.12.2441 9199370

[B400] Renner M. Lancaster M. A. Bian S. Choi H. Ku T. Peer A. (2017). Self-organized developmental patterning and differentiation in cerebral organoids. *EMBO J.* 36 1316–1329. 10.15252/embj.201694700 28283582 PMC5430225

[B401] Reubinoff B. E. Itsykson P. Turetsky T. Pera M. F. Reinhartz E. Itzik A. (2001). Neural progenitors from human embryonic stem cells. *Nat. Biotechnol.* 19 1134–1140. 10.1038/nbt1201-1134 11731782

[B402] Rhee H. J. Shaib A. H. Rehbach K. Lee C. K. Seif P. Thomas C. (2019). An autaptic culture system for standardized analyses of IPSC-derived human neurons. *Cell Rep.* 27 2212–2228.e7. 10.1016/j.celrep.2019.04.059 31091457

[B403] Ribes V. Briscoe J. (2009). Establishing and interpreting graded Sonic Hedgehog signaling during vertebrate neural tube patterning: the role of negative feedback. *Cold Spring Harb. Perspect. Biol.* 1:a002014. 10.1101/cshperspect.a002014 20066087 PMC2742090

[B404] Rice D. S. Curran T. (2001). Role of the Reelin signaling pathway in central nervous system development. *Annu. Rev. Neurosci.* 24 1005–1039. 10.1146/annurev.neuro.24.1.1005 11520926

[B405] Richardson W. D. Kessaris N. Pringle N. (2006). Oligodendrocyte wars. *Nat. Rev. Neurosci.* 7 11–18. 10.1038/nrn1826 16371946 PMC6328010

[B406] Rieger B. Junge W. Busch K. B. (2014). Lateral pH gradient between OXPHOS complex IV and F(0)F(1) ATP-synthase in folded mitochondrial membranes. *Nat. Commun.* 5:3103. 10.1038/ncomms4103 24476986

[B407] Ring K. L. Tong L. M. Balestra M. E. Javier R. Andrews-Zwilling Y. Li G. (2012). Direct reprogramming of mouse and human fibroblasts into multipotent neural stem cells with a single factor. *Cell Stem Cell* 11 100–109. 10.1016/j.stem.2012.05.018 22683203 PMC3399516

[B408] Rinholm J. E. Hamilton N. B. Kessaris N. Richardson W. D. Bergersen L. H. Attwell D. (2011). Regulation of oligodendrocyte development and myelination by glucose and lactate. *J. Neurosci.* 31 538–548. 10.1523/JNEUROSCI.3516-10.2011 21228163 PMC3044866

[B409] Rinholm J. E. Vervaeke K. Tadross M. R. Tkachuk A. N. Kopek B. G. Brown T. A. (2016). Movement and structure of mitochondria in oligodendrocytes and their myelin sheaths. *Glia* 64 810–825. 10.1002/glia.22965 26775288

[B410] Rodrigues D. C. Harvey E. M. Suraj R. Erickson S. L. Mohammad L. Ren M. (2020). Methylglyoxal couples metabolic and translational control of Notch signalling in mammalian neural stem cells. *Nat. Commun.* 11:2018. 10.1038/s41467-020-15941-2 32332750 PMC7181744

[B411] Rodrigues R. S. Lourenço D. M. Paulo S. L. Mateus J. M. Ferreira M. F. Mouro F. M. (2019). Cannabinoid actions on neural stem cells: implications for pathophysiology. *Molecules* 24:1350. 10.3390/molecules24071350 30959794 PMC6480122

[B412] Rojo M. Legros F. Chateau D. Lombès A. (2002). Membrane topology and mitochondrial targeting of mitofusins, ubiquitous mammalian homologs of the transmembrane GTPase Fzo. *J. Cell Sci.* 115 1663–1674. 10.1242/jcs.115.8.1663 11950885

[B413] Romero-Morales A. Rastogi A. Temuri H. Rasmussen M. McElroy G. S. Hsu L. (2020). Human iPSC-derived cerebral organoids model features of Leigh Syndrome and reveal abnormal corticogenesis. *bioRxiv* [Preprint] bioRxiv: 2020.04.21.054361, 10.1101/2020.04.21.054361 PMC9357378 35792828

[B414] Rosenberg M. J. Agarwala R. Bouffard G. Davis J. Fiermonte G. Hilliard M. S. (2002). Mutant deoxynucleotide carrier is associated with congenital microcephaly. *Nat. Genet.* 32 175–179. 10.1038/ng948 12185364

[B415] Ruiz I Altaba A. Jessell T. (1991). Retinoic acid modifies mesodermal patterning in early *Xenopus embryos*. *Genes Dev.* 5 175–187. 10.1101/gad.5.2.175 1671660

[B416] Saha K. Jaenisch R. (2009). Technical challenges in using human induced pluripotent stem cells to model disease. *Cell Stem Cell* 5 584–595. 10.1016/j.stem.2009.11.009 19951687 PMC2921621

[B417] Salisbury-Ruf C. T. Bertram C. C. Vergeade A. Lark D. S. Shi Q. Heberling M. L. (2018). Bid maintains mitochondrial cristae structure and function and protects against cardiac disease in an integrative genomics study. *Elife* 7:e40907. 10.7554/eLife.40907 30281024 PMC6234033

[B418] Samanta J. Kessler J. A. (2004). Interactions between ID and OLIG proteins mediate the inhibitory effects of BMP4 on oligodendroglial differentiation. *Development* 131 4131–4142. 10.1242/dev.01273 15280210

[B419] Sanderson T. H. Raghunayakula S. Kumar R. (2015). Neuronal hypoxia disrupts mitochondrial fusion. *Neuroscience* 301 71–78. 10.1016/j.neuroscience.2015.05.078 26049142 PMC4504771

[B420] Santel A. Fuller M. T. (2001). Control of mitochondrial morphology by a human mitofusin. *J. Cell Sci.* 114 867–874. 10.1242/jcs.114.5.867 11181170

[B421] Santore M. T. McClintock D. S. Lee V. Y. Budinger G. R. S. Chandel N. S. (2002). Anoxia-induced apoptosis occurs through a mitochondria-dependent pathway in lung epithelial cells. *Am. J. Physiol. Lung Cell. Mol. Physiol.* 282 L727–L734. 10.1152/ajplung.00281.2001 11880298

[B422] Sauer F. C. (1935). Mitosis in the neural tube. *J. Comp. Neurol.* 62 377–405. 10.1002/cne.900620207

[B423] Sauerland C. Menzies B. R. Glatzle M. Seeger J. Renfree M. B. Fietz S. A. (2018). The basal radial glia occurs in marsupials and underlies the evolution of an expanded neocortex in therian mammals. *Cereb. Cortex* 28 145–157. 10.1093/cercor/bhw360 29253253

[B424] Schaefer A. Lim A. Gorman G. (2019). “Epidemiology of mitochondrial disease,” in *Diagnosis and Management of Mitochondrial Disorders*, eds Mancuso M. Klopstock T. (Cham: Springer International Publishing), 63–79. 10.1007/978-3-030-05517-2_4

[B425] Schägger H. Pfeiffer K. (2001). The ratio of oxidative phosphorylation complexes i-v in bovine heart mitochondria and the composition of respiratory chain supercomplexes. *J. Biol. Chem.* 276 37861–37867. 10.1074/jbc.m106474200 11483615

[B426] Schaper A. (1897). Die frühesten differenzirungsvorgänge im centralnervensystem. *Arch. Entwickelungsmechanik Org.* 5 81–132. 10.1007/BF02153233

[B427] Schier A. F. (2003). Nodal signaling in vertebrate development. *Annu. Rev. Cell Dev. Biol.* 19 589–621. 10.1146/annurev.cellbio.19.041603.094522 14570583

[B428] Schubert M. B. Vilarinho L. (2020). Molecular basis of leigh syndrome: a current look. *Orphanet J. Rare Dis.* 15 1–14. 10.1186/s13023-020-1297-9 31996241 PMC6990539

[B429] Schwarz T. L. (2013). Mitochondrial trafficking in neurons. *Cold Spring Harb. Perspect. Med.* 5:a011304. 10.1101/cshperspect.a011304 23732472 PMC3660831

[B430] Seal S. Monsoro-Burq A. H. (2020). Insights into the early gene regulatory network controlling neural crest and placode fate choices at the neural border. *Front. Physiol.* 11:1528. 10.3389/fphys.2020.608812 33324244 PMC7726110

[B431] Sessa A. Mao C. A. Hadjantonakis A. K. Klein W. H. Broccoli V. (2008). Tbr2 directs conversion of radial glia into basal precursors and guides neuronal amplification by indirect neurogenesis in the developing neocortex. *Neuron* 60 56–69. 10.1016/j.neuron.2008.09.028 18940588 PMC2887762

[B432] Sessa A. Mao C.-A. Colasante G. Nini A. Klein W. H. Broccoli V. (2010). Tbr2-positive intermediate (basal) neuronal progenitors safeguard cerebral cortex expansion by controlling amplification of pallial glutamatergic neurons and attraction of subpallial GABAergic interneurons. *Genes Dev.* 24 1816–1826. 10.1101/gad.575410 20713522 PMC2922508

[B433] Shamseldin H. E. Alshammari M. Al-Sheddi T. Salih M. A. Alkhalidi H. Kentab A. (2012). Genomic analysis of mitochondrial diseases in a consanguineous population reveals novel candidate disease genes. *J. Med. Genet.* 49 234–241. 10.1136/jmedgenet-2012-100836 22499341

[B434] Shan Y. Zhang Y. Zhao Y. Wang T. Zhang J. Yao J. (2020). JMJD3 and UTX determine fidelity and lineage specification of human neural progenitor cells. *Nat. Commun.* 11:382. 10.1038/s41467-019-14028-x 31959746 PMC6971254

[B435] Sharpe C. R. (1991). Retinoic acid can mimic endogenous signals involved in transformation of the xenopus nervous system. *Neuron* 7 239–247. 10.1016/0896-6273(91)90262-X 1678613

[B436] Shen Q. Wang Y. Dimos J. T. Fasano C. A. Phoenix T. N. Lemischka I. R. (2006). The timing of cortical neurogenesis is encoded within lineages of individual progenitor cells. *Nat. Neurosci.* 9 743–751. 10.1038/nn1694 16680166

[B437] Shen Q. Yamano K. Head B. P. Kawajiri S. Cheung J. T. M. Wang C. (2014). Mutations in Fis1 disrupt orderly disposal of defective mitochondria. *Mol. Biol. Cell* 25 145–159. 10.1091/mbc.E13-09-0525 24196833 PMC3873885

[B438] Shi Y. Kirwan P. Smith J. Robinson H. P. C. C. Livesey F. J. (2012). Human cerebral cortex development from pluripotent stem cells to functional excitatory synapses. *Nat. Neurosci.* 15 477–486. 10.1038/nn.3041 22306606 PMC3882590

[B439] Shibata M. Gulden F. O. Sestan N. (2015). From trans to cis: transcriptional regulatory networks in neocortical development. *Trends Genet.* 31 77–87. 10.1016/j.tig.2014.12.004 25624274 PMC4382006

[B440] Shim S. Kwan K. Y. Li M. Lefebvre V. Šestan N. (2012). Cis-regulatory control of corticospinal system development and evolution. *Nature* 486 74–79. 10.1038/nature11094 22678282 PMC3375921

[B441] Shimizu T. Kagawa T. Wada T. Muroyama Y. Takada S. Ikenaka K. (2005). Wnt signaling controls the timing of oligodendrocyte development in the spinal cord. *Dev. Biol.* 282 397–410. 10.1016/j.ydbio.2005.03.020 15950605

[B442] Shook L. L. Kislal S. Edlow A. G. (2020). Fetal brain and placental programming in maternal obesity: a review of human and animal model studies. *Prenat. Diagn.* 40 1126–1137. 10.1002/pd.5724 32362000 PMC7606714

[B443] Siegenthaler J. A. Ashique A. M. Zarbalis K. Patterson K. P. Hecht J. H. Kane M. A. (2009). Retinoic acid from the meninges regulates cortical neuron generation. *Cell* 139 597–609. 10.1016/j.cell.2009.10.004 19879845 PMC2772834

[B444] Silbereis J. C. Nobuta H. Tsai H. H. Heine V. M. McKinsey G. L. Meijer D. H. (2014). Olig1 function is required to repress dlx1/2 and interneuron production in mammalian brain. *Neuron* 81 574–587. 10.1016/j.neuron.2013.11.024 24507192 PMC3979971

[B445] Sloan S. A. Andersen J. Paşca A. M. Birey F. Paşca S. P. (2018). Generation and assembly of human brain region–specific three-dimensional cultures. *Nat. Protoc.* 13 2062–2085. 10.1038/s41596-018-0032-7 30202107 PMC6597009

[B446] Sloan S. A. Darmanis S. Huber N. Khan T. A. Birey F. Caneda C. (2017). Human astrocyte maturation captured in 3d cerebral cortical spheroids derived from pluripotent stem cells. *Neuron* 95 779–790.e6. 10.1016/j.neuron.2017.07.035 28817799 PMC5890820

[B447] Smart I. H. M. Dehay C. Giroud P. Berland M. Kennedy H. (2002). Unique morphological features of the proliferative zones and postmitotic compartments of the neural epithelium giving rise to striate and extrastriate cortex in the monkey. *Cereb. Cortex* 12 37–53. 10.1093/cercor/12.1.37 11734531 PMC1931430

[B448] Smirnova E. Shurland D. L. Ryazantsev S. N. Van Der Bliek A. M. (1998). A human dynamin-related protein controls the distribution of mitochondria. *J. Cell Biol.* 143 351–358. 10.1083/jcb.143.2.351 9786947 PMC2132828

[B449] Smith J. R. Vallier L. Lupo G. Alexander M. Harris W. A. Pedersen R. A. (2008). Inhibition of Activin/Nodal signaling promotes specification of human embryonic stem cells into neuroectoderm. *Dev. Biol.* 313 107–117. 10.1016/j.ydbio.2007.10.003 18022151

[B450] Smith W. C. Harland R. M. (1992). Expression cloning of noggin, a new dorsalizing factor localized to the Spemann organizer in *Xenopus embryos*. *Cell* 70 829–840. 10.1016/0092-8674(92)90316-5 1339313

[B451] Sofou K. De Coo I. F. M. Isohanni P. Ostergaard E. Naess K. De Meirleir L. (2014). A multicenter study on Leigh syndrome: disease course and predictors of survival. *Orphanet J. Rare Dis.* 9:52. 10.1186/1750-1172-9-52 24731534 PMC4021638

[B452] Sofou K. De Coo I. F. M. Ostergaard E. Isohanni P. Naess K. De Meirleir L. (2018). Phenotype-genotype correlations in leigh syndrome: new insights from a multicentre study of 96 patients. *J. Med. Genet.* 55 21–27. 10.1136/jmedgenet-2017-104891 29101127

[B453] Son E. Y. Ichida J. K. Wainger B. J. Toma J. S. Rafuse V. F. Woolf C. J. (2011). Conversion of mouse and human fibroblasts into functional spinal motor neurons. *Cell Stem Cell* 9 205–218. 10.1016/j.stem.2011.07.014 21852222 PMC3188987

[B454] Soula C. Danesin C. Kan P. Grob M. Poncet C. Cochard P. (2001). Distinct sites of origin of oligodendrocytes and somatic motoneurons in the chick spinal cord: oligodendrocytes arise from Nkx2.2-expressing progenitors by a Shh-dependent mechanism. *Development* 128 1369–1379. 10.1242/dev.128.8.1369 11262237

[B455] Sousa A. M. M. Meyer K. A. Santpere G. Gulden F. O. Sestan N. (2017). Evolution of the human nervous system function. *Struct. Dev. Cell* 170 226–247. 10.1016/j.cell.2017.06.036 28708995 PMC5647789

[B456] Spear P. C. Erickson C. A. (2012). Interkinetic nuclear migration: a mysterious process in search of a function. *Dev. Growth Differ.* 54 306–316. 10.1111/j.1440-169X.2012.01342.x 22524603 PMC3357188

[B457] Spemann H. Mangold H. (1924). Über induktion von embryonalanlagen durch implantation artfremder organisatoren. *Arch. Mikroskopische Anat. Entwicklungsmechanik* 100 599–638. 10.1007/BF02108133

[B458] Spemann H. Mangold H. (2001). Induction of embryonic primordia by implantation of organizers from a different species. *Int. J. Dev. Biol.* 45 13–38. 10.1387/ijdb.11291841 11291841

[B459] Spiegel R. Saada A. Flannery P. J. Burté F. Soiferman D. Khayat M. (2016). Fatal infantile mitochondrial encephalomyopathy, hypertrophic cardiomyopathy and optic atrophy associated with a homozygous OPA1 mutation. *J. Med. Genet.* 53 127–131. 10.1136/jmedgenet-2015-103361 26561570 PMC4752660

[B460] Srinivasan K. Leone D. P. Bateson R. K. Dobreva G. Kohwi Y. Kohwi-Shigematsu T. (2012). A network of genetic repression and derepression specifies projection fates in the developing neocortex. *Proc. Natl. Acad. Sci.U.S. A.* 109 19071–19078. 10.1073/pnas.1216793109 23144223 PMC3511157

[B461] Steffann J. Gigarel N. Corcos J. Bonnière M. Encha-Razavi F. Sinico M. (2007). Stability of the m.8993T→G mtDNA mutation load during human embryofetal development has implications for the feasibility of prenatal diagnosis in NARP syndrome. *J. Med. Genet.* 44 664–669. 10.1136/jmg.2006.048553 17545557 PMC2597968

[B462] Steinman K. J. Gorno-Tempini M. L. Glidden D. V. Kramer J. H. Miller S. P. Barkovich A. J. (2009). Neonatal watershed brain injury on magnetic resonance imaging correlates with verbal IQ at 4 years. *Pediatrics* 123 1025–1030. 10.1542/peds.2008-1203 19255035 PMC2718837

[B463] Stolt C. C. Rehberg S. Ader M. Lommes P. Riethmacher D. Schachner M. (2002). Terminal differentiation of myelin-forming oligodendrocytes depends on the transcription factor Sox10. *Genes Dev.* 16 165–170. 10.1101/gad.215802 11799060 PMC155320

[B464] Stolt C. C. Schlierf A. Lommes P. Hillgärtner S. Werner T. Kosian T. (2006). SoxD proteins influence multiple stages of oligodendrocyte development and modulate soxe protein function. *Dev. Cell* 11 697–709. 10.1016/j.devcel.2006.08.011 17084361

[B465] Strauss M. Hofhaus G. Schröder R. R. Kühlbrandt W. (2008). Dimer ribbons of ATP synthase shape the inner mitochondrial membrane. *EMBO J.* 27 1154–1160. 10.1038/emboj.2008.35 18323778 PMC2323265

[B466] Sugimori M. Nagao M. Parras C. M. Nakatani H. Lebel M. Guillemot F. (2008). Ascl1 is required for oligodendrocyte development in the spinal cord. *Development* 135 1271–1281. 10.1242/dev.015370 18287202

[B467] Sugitani Y. Nakai S. Minowa O. Nishi M. Jishage K. Kawano H. (2002). Brn-1 and Brn-2 share crucial roles in the production and positioning of mouse neocortical neurons. *Genes Dev.* 16 1760–1765. 10.1101/gad.978002 12130536 PMC186401

[B468] Sun Y. Nadal-Vicens M. Misono S. Lin M. Z. Zubiaga A. Hua X. (2001). Neurogenin promotes neurogenesis and inhibits glial differentiation by independent mechanisms. *Cell* 104 365–376. 10.1016/S0092-8674(01)00224-0 11239394

[B469] Suter D. M. Tirefort D. Julien S. Krause K.-H. (2009). A Sox1 to Pax6 switch drives neuroectoderm to radial glia progression during differentiation of mouse embryonic stem cells. *Stem Cells* 27 49–58. 10.1634/stemcells.2008-0319 18832594

[B470] Taguchi N. Ishihara N. Jofuku A. Oka T. Mihara K. (2007). Mitotic phosphorylation of dynamin-related GTPase Drp1 participates in mitochondrial fission. *J. Biol. Chem.* 282 11521–11529. 10.1074/jbc.M607279200 17301055

[B471] Takahashi K. Yamanaka S. (2006). Induction of pluripotent stem cells from mouse embryonic and adult fibroblast cultures by defined factors. *Cell* 126 663–676. 10.1016/j.cell.2006.07.024 16904174

[B472] Takahashi K. Tanabe K. Ohnuki M. Narita M. Ichisaka T. Tomoda K. (2007). Induction of pluripotent stem cells from adult human fibroblasts by defined factors. *Cell* 131 861–872. 10.1016/j.cell.2007.11.019 18035408

[B473] Takizawa T. Nakashima K. Namihira M. Ochiai W. Uemura A. Yanagisawa M. (2001). DNA methylation is a critical cell-intrinsic determinant of astrocyte differentiation in the fetal brain. *Dev. Cell* 1 749–758. 10.1016/S1534-5807(01)00101-0 11740937

[B474] Tao W. Lai E. (1992). Telencephalon-restricted expression of BF-1, a new member of the HNF-3/fork head gene family, in the developing rat brain. *Neuron* 8 957–966. 10.1016/0896-6273(92)90210-5 1350202

[B475] Tao Y. Zhang S. C. (2016). Neural subtype specification from human pluripotent stem cells. *Cell Stem Cell* 19 573–586. 10.1016/j.stem.2016.10.015 27814479 PMC5127287

[B476] Tarabykin V. Stoykova A. Usman N. Gruss P. (2001). Cortical upper layer neurons derive from the subventricular zone as indicated by Svet1 gene expression. *Development* 128 1983–1993. 10.1242/dev.128.11.1983 11493521

[B477] Taverna E. Götz M. Huttner W. B. (2014). The cell biology of neurogenesis: toward an understanding of the development and evolution of the neocortex. *Annu. Rev. Cell Dev. Biol.* 30 465–502. 10.1146/annurev-cellbio-101011-155801 25000993

[B478] Tcw J. Wang M. Pimenova A. A. Bowles K. R. Hartley B. J. Lacin E. (2017). An efficient platform for astrocyte differentiation from human induced pluripotent stem cells. *Stem Cell Rep.* 9 600–614. 10.1016/j.stemcr.2017.06.018 28757165 PMC5550034

[B479] Ten V. S. Starkov A. (2012). Hypoxic-ischemic injury in the developing brain: the role of reactive oxygen species originating in mitochondria. *Neurol. Res. Int.* 2012 1–10. 10.1155/2012/542976 22548167 PMC3323863

[B480] Teslaa T. Teitell M. A. (2015). Pluripotent stem cell energy metabolism: an update. *EMBO J.* 34 138–153. 10.15252/embj.201490446 25476451 PMC4337063

[B481] Thakurela S. Tiwari N. Schick S. Garding A. Ivanek R. Berninger B. (2016). Mapping gene regulatory circuitry of Pax6 during neurogenesis. *Cell Discov.* 2 1–22. 10.1038/celldisc.2015.45 27462442 PMC4860964

[B482] Thomson J. A. Marshall V. S. (1998). Primate embryonic stem cells. *Curr. Top. Dev. Biol.* 38 133–165. 10.1016/s0070-2153(08)60246-x 9399078

[B483] Thomson J. A. Itskovitz-Eldor J. Shapiro S. S. Waknitz M. A. Swiergiel J. J. Marshall V. S. (1998). Embryonic stem cell lines derived from human blastocysts. *Science* 282 1145–1147. 10.1126/science.282.5391.1145 9804556

[B484] Thomson J. A. Kalishman J. Golos T. G. Durning M. Harris C. P. Becker R. A. (1995). Isolation of a primate embryonic stem cell line. *Proc. Natl. Acad. Sci. U.S.A.* 92 7844–7848. 10.1073/pnas.92.17.7844 7544005 PMC41242

[B485] Tiberi L. van den Ameele J. Dimidschstein J. Piccirilli J. Gall D. Herpoel A. (2012). BCL6 controls neurogenesis through Sirt1-dependent epigenetic repression of selective Notch targets. *Nat. Neurosci.* 15 1627–1635. 10.1038/nn.3264 23160044

[B486] Tieu Q. Okreglak V. Naylor K. Nunnari J. (2002). The WD repeat protein, Mdv1p, functions as a molecular adaptor by interacting with Dnm1p and Fis1p during mitochondrial fission. *J. Cell Biol.* 158 445–452. 10.1083/jcb.200205031 12163467 PMC2173813

[B487] Tissir F. Goffinet A. M. (2003). Reelin and brain development. *Nat. Rev. Neurosci.* 4 496–505. 10.1038/nrn1113 12778121

[B488] Toma K. Kumamoto T. Hanashima C. Toma K. Hanashima C. (2014). The timing of upper-layer neurogenesis is conferred by sequential derepression and negative feedback from deep-layer neurons. *J. Neurosci.* 34 13259–13276. 10.1523/JNEUROSCI.2334-14.2014 25253869 PMC6608336

[B489] Toresson H. De Urquiza A. M. Fagerström C. Perlmann T. Campbell K. (1999). Retinoids are produced by glia in the lateral ganglionic eminence and regulate striatal neuron differentiation. *Development* 126 1317–1326. 10.1242/dev.126.6.1317 10021349

[B490] Trommsdorff M. Gotthardt M. Hiesberger T. Shelton J. Stockinger W. Nimpf J. (1999). Reeler/disabled-like disruption of neuronal migration in knockout mice lacking the VLDL receptor and ApoE receptor 2. *Cell* 97 689–701. 10.1016/S0092-8674(00)80782-5 10380922

[B491] Valenzuela D. M. Economides A. N. Rojas E. Lamb T. M. Nuñez L. Jones P. (1995). Identification of mammalian noggin and its expression in the adult nervous system. *J. Neurosci.* 15 6077–6084. 10.1523/jneurosci.15-09-06077.1995 7666191 PMC6577675

[B492] van den Ameele J. Brand A. H. (2019). Neural stem cell temporal patterning and brain tumour growth rely on oxidative phosphorylation. *Elife* 8:e47887. 10.7554/eLife.47887 31513013 PMC6763261

[B493] Vander Heiden M. G. Chandel N. S. Williamson E. K. Schumacker P. T. Thompson C. B. (1997). Bcl-x(L) regulates the membrane potential and volume homeostasis of mitochondria. *Cell* 91 627–637. 10.1016/s0092-8674(00)80450-x 9393856

[B494] Vasistha N. A. García-Moreno F. Arora S. Cheung A. F. P. Arnold S. J. Robertson E. J. (2015). Cortical and clonal contribution of Tbr2 expressing progenitors in the developing mouse brain. *Cereb. Cortex* 25 3290–3302. 10.1093/cercor/bhu125 24927931 PMC4585488

[B495] Velasco S. Kedaigle A. J. Simmons S. K. Nash A. Rocha M. Quadrato G. (2019). Individual brain organoids reproducibly form cell diversity of the human cerebral cortex. *Nature* 570 523–527. 10.1038/s41586-019-1289-x 31168097 PMC6906116

[B496] Verny C. Loiseau D. Scherer C. Lejeune P. Chevrollier A. Gueguen N. (2008). Multiple sclerosis-like disorder in OPA1-related autosomal dominant optic atrophy. *Neurology* 70 1152–1153. 10.1212/01.wnl.0000289194.89359.a1 18287570

[B497] Vierbuchen T. Ostermeier A. Pang Z. P. Kokubu Y. Südhof T. C. Wernig M. (2010). Direct conversion of fibroblasts to functional neurons by defined factors. *Nature* 463 1035–1041. 10.1038/nature08797 20107439 PMC2829121

[B498] Villalón-García I. Álvarez-Córdoba M. Suárez-Rivero J. M. Povea-Cabello S. Talaverón-Rey M. Suárez-Carrillo A. (2020). Precision medicine in rare diseases. *Diseases* 8:42. 10.3390/diseases8040042 33202892 PMC7709101

[B499] Villanueva-Paz M. Povea-Cabello S. Villalón-García I. Suárez-Rivero J. M. Álvarez-Córdoba M. de la Mata M. (2019). Pathophysiological characterization of MERRF patient-specific induced neurons generated by direct reprogramming. *Biochim. Biophys. Acta Mol. Cell Res.* 1866 861–881. 10.1016/j.bbamcr.2019.02.010 30797798

[B500] Volpato V. Webber C. (2020). Addressing variability in iPSC-derived models of human disease: guidelines to promote reproducibility. *Dis. Model. Mech.* 13:dmm042317. 10.1242/dmm.042317 31953356 PMC6994963

[B501] Volpe J. J. (2012). Neonatal encephalopathy: an inadequate term for hypoxic-ischemic encephalopathy. *Ann. Neurol.* 72 156–166. 10.1002/ana.23647 22926849

[B502] Wakabayashi J. Zhang Z. Wakabayashi N. Tamura Y. Fukaya M. Kensler T. W. (2009). The dynamin-related GTPase Drp1 is required for embryonic and brain development in mice. *J. Cell Biol.* 186 805–816. 10.1083/jcb.200903065 19752021 PMC2753156

[B503] Walsh C. Cepko C. L. (1993). Clonal dispersion in proliferative layers of developing cerebral cortex. *Nature* 362 632–635. 10.1038/362632a0 8464513

[B504] Wang L. Bluske K. K. Dickel L. K. Nakagawa Y. (2011). Basal progenitor cells in the embryonic mouse thalamus – their molecular characterization and the role of neurogenins and Pax6. *Neural Dev.* 6 1–19. 10.1186/1749-8104-6-35 22077982 PMC3234181

[B505] Wang X. Tsai J. W. Lamonica B. Kriegstein A. R. (2011). A new subtype of progenitor cell in the mouse embryonic neocortex. *Nat. Neurosci.* 14 555–562. 10.1038/nn.2807 21478886 PMC3083489

[B506] Warren E. B. Aicher A. E. Fessel J. P. Konradi C. (2017). Mitochondrial DNA depletion by ethidium bromide decreases neuronal mitochondrial creatine kinase: Implications for striatal energy metabolism. *PLoS One* 12:e0190456. 10.1371/journal.pone.0190456 29287112 PMC5747477

[B507] Watanabe K. Kamiya D. Nishiyama A. Katayama T. Nozaki S. Kawasaki H. (2005). Directed differentiation of telencephalic precursors from embryonic stem cells. *Nat. Neurosci.* 8 288–296. 10.1038/nn1402 15696161

[B508] Watanabe K. Ueno M. Kamiya D. Nishiyama A. Matsumura M. Wataya T. (2007). A ROCK inhibitor permits survival of dissociated human embryonic stem cells. *Nat. Biotechnol.* 25 681–686. 10.1038/nbt1310 17529971

[B509] Waterham H. R. Koster J. van Roermund C. W. T. Mooyer P. A. W. Wanders R. J. A. Leonard J. V. (2007). A lethal defect of mitochondrial and peroxisomal fission. *N. Engl. J. Med.* 356 1736–1741. 10.1056/nejmoa064436 17460227

[B510] Wei M. C. Lindsten T. Mootha V. K. Weiler S. Gross A. Ashiya M. (2000). tBID, a membrane-targeted death ligand, oligomerizes BAK to release cytochrome c. *Genes Dev.* 14 2060–2071. 10.1101/gad.14.16.2060 10950869 PMC316859

[B511] Weidemann A. Johnson R. S. (2008). Biology of HIF-1α. *Cell Death Differ.* 15 621–627. 10.1038/cdd.2008.12 18259201

[B512] Weimann J. M. Zhang Y. A. Levin M. E. Devine W. P. Brûlet P. McConnell S. K. (1999). Cortical neurons require Otx1 for the refinement of exuberant axonal projections to subcortical targets. *Neuron* 24 819–831. 10.1016/S0896-6273(00)81030-2 10624946

[B513] White C. J. Lee J. Choi J. Chu T. Scafidi S. Wolfgang M. J. (2020). Determining the bioenergetic capacity for fatty acid oxidation in the mammalian nervous system. *Mol. Cell. Biol.* 40:e00037–20. 10.1128/mcb.00037-20 32123009 PMC7189099

[B514] White S. L. Collins V. R. Wolfe R. Cleary M. A. Shanske S. DiMauro S. (1999). Genetic counseling and prenatal diagnosis for the mitochondrial DNA mutations at nucleotide 8993. *Am. J. Hum. Genet.* 65 474–482. 10.1086/302488 10417290 PMC1377946

[B515] Wilkens V. Kohl W. Busch K. (2013). Restricted diffusion of OXPHOS complexes in dynamic mitochondria delays their exchange between cristae and engenders a transitory mosaic distribution. *J. Cell Sci.* 126 103–116. 10.1242/jcs.108852 23038773

[B516] Wodarz A. Huttner W. B. (2003). Asymmetric cell division during neurogenesis in *Drosophila* and vertebrates. *Mech. Dev.* 120 1297–1309. 10.1016/j.mod.2003.06.003 14623439

[B517] Wu S. X. Goebbels S. Nakamura K. Nakamura K. Kometani K. Minato N. (2005). Pyramidal neurons of upper cortical layers generated by NEX-positive progenitor cells in the subventricular zone. *Proc. Natl. Acad. Sci. U.S A.* 102 17172–17177. 10.1073/pnas.0508560102 16284248 PMC1288007

[B518] Xiang Y. Tanaka Y. Cakir B. Patterson B. Kim K.-Y. Sun P. (2019). hESC-derived thalamic organoids form reciprocal projections when fused with cortical organoids. *Cell Stem Cell* 24 487–497.e7. 10.1016/j.stem.2018.12.015 30799279 PMC6853597

[B519] Xiang Y. Tanaka Y. Patterson B. Kang Y. J. Govindaiah G. Roselaar N. (2017). Fusion of regionally specified hpsc-derived organoids models human brain development and interneuron migration. *Cell Stem Cell* 21 383–398.e7. 10.1016/j.stem.2017.07.007 28757360 PMC5720381

[B520] Xie Y. W. Wolin M. S. (1996). Role of nitric oxide and its interaction with superoxide in the suppression of cardiac muscle mitochondrial respiration: involvement in response to hypoxia/reoxygenation. *Circulation* 94 2580–2586. 10.1161/01.CIR.94.10.2580 8921804

[B521] Xie Z. Jones A. Deeney J. T. Hur S. K. Bankaitis V. A. (2016). Inborn errors of long-chain fatty acid β-oxidation link neural stem cell self-renewal to autism. *Cell Rep.* 14 991–999. 10.1016/j.celrep.2016.01.004 26832401 PMC4749429

[B522] Yamaguchi T. P. (2001). Heads or tails: Wnts and anterior-posterior patterning. *Curr. Biol.* 11 R713–R724. 10.1016/S0960-9822(01)00417-1 11553348

[B523] Yamaguchi Y. Miura M. (2015). Programmed cell death in neurodevelopment. *Dev. Cell* 32 478–490. 10.1016/j.devcel.2015.01.019 25710534

[B524] Yamanaka S. (2020). Pluripotent stem cell-based cell therapy—promise and challenges. *Cell Stem Cell* 27 523–531. 10.1016/j.stem.2020.09.014 33007237

[B525] Yang X. Xu B. Mulvey B. Evans M. Jordan S. Wang Y.-D. (2019). Differentiation of human pluripotent stem cells into neurons or cortical organoids requires transcriptional co-regulation by UTX and 53BP1. *Nat. Neurosci.* 22 362–373. 10.1038/s41593-018-0328-5 30718900 PMC6511450

[B526] Ybot-Gonzalez P. Gaston-Massuet C. Girdler G. Klingensmith J. Arkell R. Greene N. D. E. (2007). Neural plate morphogenesis during mouse neurulation is regulated by antagonism of Bmp signalling. *Development* 134 3203–3211. 10.1242/dev.008177 17693602

[B527] Yoo A. S. Sun A. X. Li L. Shcheglovitov A. Portmann T. Li Y. (2011). MicroRNA-mediated conversion of human fibroblasts to neurons. *Nature* 476 228–231. 10.1038/nature10323 21753754 PMC3348862

[B528] Yoon S. J. Elahi L. S. Paşca A. M. Marton R. M. Gordon A. Revah O. (2019). Reliability of human cortical organoid generation. *Nat. Methods* 16 75–78. 10.1038/s41592-018-0255-0 30573846 PMC6677388

[B529] Yu-Wai-Man P. Griffiths P. G. Gorman G. S. Lourenco C. M. Wright A. F. Auer-Grumbach M. (2010). Multi-system neurological disease is common in patients with OPA1 mutations. *Brain* 133 771–786. 10.1093/brain/awq007 20157015 PMC2842512

[B530] Zahedi A. On V. Phandthong R. Chaili A. Remark G. Bhanu B. (2018). Deep analysis of mitochondria and cell health using machine learning. *Sci. Rep.* 8:16354. 10.1038/s41598-018-34455-y 30397207 PMC6218515

[B531] Zecevic N. Chen Y. Filipovic R. (2005). Contributions of cortical subventricular zone to the development of the human cerebral cortex. *J. Comp. Neurol.* 491 109–122. 10.1002/cne.20714 16127688 PMC2628573

[B532] Zehnder T. Petrelli F. Romanos J. De Oliveira Figueiredo E. C. Lewis T. L. Déglon N. (2021). Mitochondrial biogenesis in developing astrocytes regulates astrocyte maturation and synapse formation. *Cell Rep.* 35:108952. 10.1016/j.celrep.2021.108952 33852851

[B533] Zeng H. Guo M. Martins-Taylor K. Wang X. Zhang Z. Park J. W. (2010). Specification of region-specific neurons including forebrain glutamatergic neurons from human induced pluripotent stem cells. *PLoS One* 5:e11853. 10.1371/journal.pone.0011853 20686615 PMC2912324

[B534] Zhadanov A. B. Provance D. W. Speer C. A. Coffin J. D. Goss D. Blixt J. A. (1999). Absence of the tight junctional protein AF-6 disrupts epithelial cell-cell junctions and cell polarity during mouse development. *Curr. Biol.* 9 880–888. 10.1016/S0960-9822(99)80392-3 10469590

[B535] Zhang H. Menzies K. J. Auwerx J. (2018). The role of mitochondria in stem cell fate and aging. *Development* 145:dev143420. 10.1242/dev.143420 29654217 PMC5964648

[B536] Zhang J. Khvorostov I. Hong J. S. Oktay Y. Vergnes L. Nuebel E. (2011). UCP2 regulates energy metabolism and differentiation potential of human pluripotent stem cells. *EMBO J.* 30 4860–4873. 10.1038/emboj.2011.401 22085932 PMC3243621

[B537] Zhang M. Ngo J. Pirozzi F. Sun Y.-P. Wynshaw-Boris A. (2018). Highly efficient methods to obtain homogeneous dorsal neural progenitor cells from human and mouse embryonic stem cells and induced pluripotent stem cells. *Stem Cell Res. Ther.* 9:67. 10.1186/s13287-018-0812-6 29544541 PMC5856210

[B538] Zhang S.-C. Wernig M. Duncan I. D. Brüstle O. Thomson J. A. (2001). In vitro differentiation of transplantable neural precursors from human embryonic stem cells. *Nat. Biotechnol.* 19 1129–1133. 10.1038/nbt1201-1129 11731781

[B539] Zhang Y. Pak C. H. Han Y. Ahlenius H. Zhang Z. Chanda S. (2013). Rapid single-step induction of functional neurons from human pluripotent stem cells. *Neuron* 78 785–798. 10.1016/j.neuron.2013.05.029 23764284 PMC3751803

[B540] Zheng X. Boyer L. Jin M. Mertens J. Kim Y. Ma L. (2016). Metabolic reprogramming during neuronal differentiation from aerobic glycolysis to neuronal oxidative phosphorylation. *Elife* 5:e13374. 10.7554/eLife.13374 27282387 PMC4963198

[B541] Zimmer C. Tiveron M. C. Bodmer R. Cremer H. (2004). Dynamics of Cux2 expression suggests that an early pool of SVZ precursors is fated to become upper cortical layer neurons. *Cereb. Cortex* 14 1408–1420. 10.1093/cercor/bhh102 15238450

[B542] Züchner S. Mersiyanova I. V. Muglia M. Bissar-Tadmouri N. Rochelle J. Dadali E. L. (2004). Mutations in the mitochondrial GTPase mitofusin 2 cause charcot-marie-tooth neuropathy type 2A. *Nat. Genet.* 36 449–451. 10.1038/ng1341 15064763

[B543] Zurita-Díaz F. Galera-Monge T. Moreno-Izquierdo A. Fraga M. F. Ayuso C. Fernández A. F. (2016). Generation of a human iPSC line from a patient with a mitochondrial encephalopathy due to mutations in the GFM1 gene. *Stem Cell Res.* 16 124–127. 10.1016/j.scr.2015.12.019 27345796

[B544] Zweifel S. Marcy G. Lo Guidice Q. Li D. Heinrich C. Azim K. (2018). HOPX defines heterogeneity of postnatal subventricular zone neural stem cells. *Stem Cell Rep.* 11 770–783. 10.1016/j.stemcr.2018.08.006 30174314 PMC6135899

